# Discovery of archaeal fusexins homologous to eukaryotic HAP2/GCS1 gamete fusion proteins

**DOI:** 10.1038/s41467-022-31564-1

**Published:** 2022-07-06

**Authors:** David Moi, Shunsuke Nishio, Xiaohui Li, Clari Valansi, Mauricio Langleib, Nicolas G. Brukman, Kateryna Flyak, Christophe Dessimoz, Daniele de Sanctis, Kathryn Tunyasuvunakool, John Jumper, Martin Graña, Héctor Romero, Pablo S. Aguilar, Luca Jovine, Benjamin Podbilewicz

**Affiliations:** 1 Instituto de Fisiología, Biología Molecular y Neurociencias (IFIBYNE-CONICET), Buenos Aires, Argentina; 2 Department of Computational Biology, University of Lausanne, Lausanne, Switzerland; 3 Swiss Institute of Bioinformatics, Lausanne, Switzerland; 4 Department of Biosciences and Nutrition, Karolinska Institutet, Huddinge, Sweden; 5 Department of Biology, Technion- Israel Institute of Technology, Haifa, Israel; 6 Unidad de Genómica Evolutiva, Facultad de Ciencias, Universidad de la República, Montevideo, Uruguay; 7 Unidad de Bioinformática, Institut Pasteur de Montevideo, Montevideo, Uruguay; 8 Department of Genetics, Evolution and Environment, Centre for Life’s Origins and Evolution, University College London, London, UK; 9 Department of Computer Science, University College London, London, UK; 10 ESRF—The European Synchrotron, Grenoble, France; 11 DeepMind, London, UK; 12 Centro Universitario Regional Este - CURE, Centro Interdisciplinario de Ciencia de Datos y Aprendizaje Automático - CICADA, Universidad de la República, Montevideo, Uruguay; 13 Instituto de Investigaciones Biotecnológicas Universidad Nacional de San Martín (IIB-CONICET), San Martín, Buenos Aires, Argentina

**Keywords:** Archaeal evolution, X-ray crystallography, Cell adhesion

## Abstract

Sexual reproduction consists of genome reduction by meiosis and subsequent gamete fusion. The presence of genes homologous to eukaryotic meiotic genes in archaea and bacteria suggests that DNA repair mechanisms evolved towards meiotic recombination. However, fusogenic proteins resembling those found in gamete fusion in eukaryotes have so far not been found in prokaryotes. Here, we identify archaeal proteins that are homologs of fusexins, a superfamily of fusogens that mediate eukaryotic gamete and somatic cell fusion, as well as virus entry. The crystal structure of a trimeric archaeal fusexin (Fusexin1 or Fsx1) reveals an archetypical fusexin architecture with unique features such as a six-helix bundle and an additional globular domain. Ectopically expressed Fusexin1 can fuse mammalian cells, and this process involves the additional globular domain and a conserved fusion loop. Furthermore, archaeal fusexin genes are found within integrated mobile elements, suggesting potential roles in cell-cell fusion and gene exchange in archaea, as well as different scenarios for the evolutionary history of fusexins.

## Abstract (web summary)

Sexual reproduction in eukaryotes involves gamete fusion, mediated by fusogenic proteins. Here, the authors identify fusogenic protein homologs encoded within mobile genetic elements in archaeal genomes, solve the crystal structure of one of the proteins, and show that its ectopic expression can fuse mammalian cells, suggesting potential roles in cell-cell fusion and gene exchange.

## Introduction

How early eukaryotes developed the capacity for gamete fusion is a central question entangled with the origin of the eukaryotic cell itself. The widespread presence of a conserved set of meiosis, gamete, and nuclear fusion proteins among extant eukaryotes indicates that meiotic sex emerged once, predating the last eukaryotic common ancestor (LECA)^1,2^. Two essential molecular events are required for meiotic sex: DNA recombination and plasma membrane fusion. Prokaryotic cells contain DNA repair machines that may have been precursors of the recombination machinery used in eukaryotic meiosis^1,2^. However, the genes encoding for proteins that are essential and sufficient to merge plasma membranes have not been identified in prokaryotes^3^.

In eukaryotes, different families of cellular and viral fusion proteins (fusogens) have been described^3^. For example, class I viral fusogens include the spike glycoproteins of Influenza, HIV, Ebola, and SARS-CoV that have similar structures that appear to have converged during evolution as a way to merge viral and eukaryotic membranes^4–6^. More recently it was shown that myoblast fusion requires two unrelated proteins to form muscles in vertebrates^7,8^. However, many fusogens have not been identified yet, and the molecular basis of gamete fusion in fungi and vertebrates remain unclear^9^.

The first eukaryotic cell–cell fusogen was discovered only two decades ago by genetic screens in *C. elegans*^10^. The fusogen EFF-1 and its paralog AFF-1 fuse one-third of all the somatic cells in the skin, excretory, reproductive, nervous and digestive systems of nematodes. The functions of these cellular fusions are to sculpt cells, tissues, and organs to restrict cellular fates for a robust development^11^. In the absence of sequence similarity between EFF-1, AFF-1, and other known proteins, predictions of the structure of AFF-1 suggested structural similarity to class II viral fusogens^12^. EFF-1 and AFF-1 can fuse cells in *C. elegans*, promote fusion between heterologous cells, and substitute a viral fusogen to mediate plasma membrane fusion only when these fusogenic proteins are expressed in both fusing membranes^13–15^. The crystal structure of the ectodomain of EFF-1 demonstrated structural similarity with class II viral fusogens such as the glycoproteins on the surface of Zika, dengue, and rubella viruses^16^. Thus, these eukaryotic and viral fusogens have remarkably similar functions and structures despite undetectable sequence similarity. However, their mechanisms of membrane fusion are different because EFF-1 and AFF-1 use a bilateral mechanism while class II viral fusogens use a unilateral mechanism^13–15^.

More recently, genetic screens uncovered a protein involved in gamete fusion, HAP2/GCS1, which is conserved in *Arabidopsis*, *Chlamydomonas*, *Plasmodium*, *Tetrahymena,* and *Dictyostelium*^17–21^. Later, structural bioinformatics, crystallographic structure elucidation and functional assays demonstrated that HAP2/GCS1 is a bona fide fusogen homologous to EFF-1, AFF-1, and class II viral fusogens^22–24^. The crystal structures of HAP2/GCS1 from *Chlamydomonas* and *Arabidopsis* showed remarkable structural conservation without sequence similarity^23,25,26^. This superfamily of fusion proteins was named fusexins: fusion proteins essential for sexual reproduction and exoplasmic merger of plasma membranes^22–24^. Thus, the fusexin superfamily encompasses class II viral fusogens (viral fusexins) that fuse the envelope of some animal viruses with the membranes of host cells during infection^5,6^; EFF-1 and AFF-1 (somatic fusexins) that promote cell fusion during syncytial organ development^10,13,15,16^; and HAP2/GCS1 (sexual fusexins; hereafter referred to as HAP2) that mediate gamete fusion^17–19^.

Although it is assumed that sexual fusexins were already present in the LECA^1,27^, their shared ancestry with viral fusexins posed a “the virus or the egg” evolutionary dilemma^22,24,28^. In one scenario, fusexins are proper eukaryal innovations that were captured by some viruses and used for host invasion. Alternatively, a viral fusexin gene was transferred to an early eukaryotic cell and then repurposed for gamete fusion. Solving this evolutionary conundrum is not a trivial task because sequence-based phylogenetics cannot be applied to the whole fusexin superfamily due to lack of sequence conservation.

Here we identify a family of fusexins in genomes of Archaea and prokaryotic fractions of metagenomes from very diverse environments. We provide crystallographic and functional evidence suggesting that these proteins are cellular fusogens. Genomic analyses show that archaeal fusexins are carried by integrated mobile genetic elements. Evolutionary analyses of the whole fusexin superfamily reveal alternative working models regarding the relationships between viral, eukaryotic and archaeal fusexins and the emergence of meiotic sex during eukaryogenesis.

## Results

### *Fusexin* genes in Archaea

**Fig. 1:**
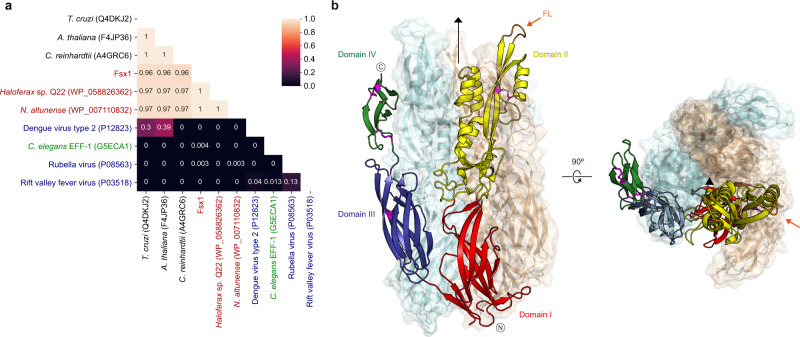
Fsx1 is a member of the fusexin protein superfamily. **a** HMM vs. HMM homology probabilities of a subset of eukaryotic, viral, and archaeal fusexin ectodomains. With exception of Fsx1, which derives from a metagenomic sequence, archaeal fusexins (red), viral fusexins (blue), EFF-1 (green) and HAP2s (black) are indicated by RefSeq/UniProt identifiers. **b** Crystal structure of the trimeric ectodomain of Fsx1. The three-fold non-crystallographic axis is indicated. Subunit A is shown as a cartoon colored by domain, with disulfides and the fusion loop (FL) colored magenta and orange, respectively; subunits B, C are in mixed cartoon/surface representation.

To search for fusexins we used the crystallographic structures of *C. reinhardtii*, *A. thaliana*, and *T. cruzi* HAP2 (Cr/At/TcHAP2)^23,25,26^ to build dedicated Hidden Markov Models (HMMs) for scanning the Uniclust30 database. We detected 24 high-confidence candidates in prokaryotes: 8 belong to isolated and cultivated archaea, and the remaining 16 to metagenome-assembled genomes (MAGs, Supplementary Table 1). We then built HMMs of the candidate ectodomains and compared them to HMMs of sexual, somatic, and viral fusexins. Figure 1a shows that the prokaryotic candidates have detectable sequence similarities with HAP2, with *E*-values below 0.001 and HHblits-derived probabilities higher than 0.95 (Supplementary Fig. 1a). We named these proteins Fusexin1 (Fsx1). *fsx1* genes found in cultivated and isolated prokaryotes are restricted to the Haloarchaea class (also called Halobacteria, Euryarchaeota superphylum) whereas MAGs containing Fsx1s include all major Archaea superphyla (Supplementary Table 1). Next, we used this Fsx1 sequence set to search the Metaclust database, which comprises 1.59 billion clustered proteins from over 2200 metagenomic/metatranscriptomic datasets. Performing a scan pipeline using PHMMER, PSI-BLAST, HMM–HMM comparisons and topology filtering we found 96 high-confidence *fsx1* genes. The identified *fsx1*s come from different environments (with preeminence of saline samples) and a wide temperature range (−35 to 80 °C, see Supplementary Data 1).

### Fsx1 is a structural homolog of HAP2/GCS1

To experimentally investigate the presence of fusexin-like proteins in Archaea, a selection of the candidate genes was expressed in mammalian cells (Supplementary Fig. 1b, c). High-level expression was observed for a metagenomic Fsx1 sequence from a hypersaline environment, predicted to encode a ~55 kDa ectodomain region (Fsx1_E_) followed by three transmembrane domains (TMs) (Supplementary Data 1). Fsx1_E_ is a monomer in solution but crystallized as a homotrimer in the presence of 2.5 M NaCl, 0.2 M CaCl_2_ (Supplementary Fig. 2). These conditions precluded experimental phasing, and attempts to phase the data by molecular replacement (MR) with different kinds of homology models also failed, due to insufficient sequence identity to known fusexin structures. However, we could determine the structure of Fsx1_E_ at 2.3 Å resolution by running MR with a combination of fragments from ab initio predictions generated by AlphaFold2^29^ (Fig. 1b, Supplementary Figs. 3, 4 and Supplementary Table 2).

**Fig. 2:**
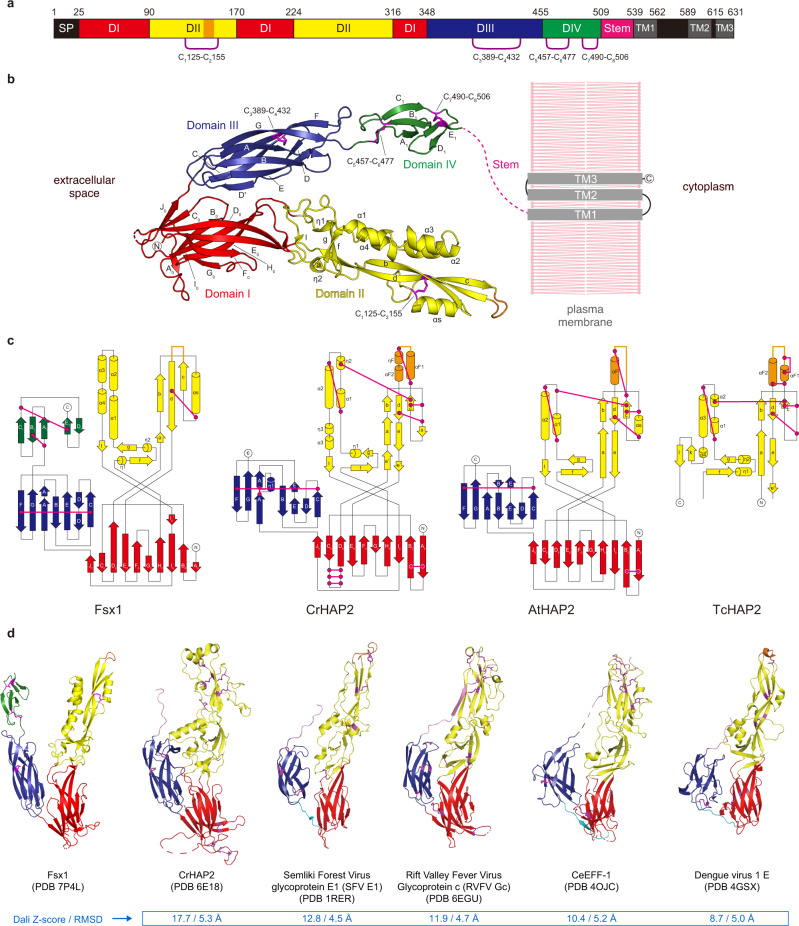
Domain architecture of Fsx1 and topological comparison with HAP2. **a** Schematic diagram of the domains of Fsx1. SP, signal peptide; TM, predicted transmembrane helices. Note that Fsx1 is predicted to contain three C-terminal TMs, with a cytoplasmic loop between TM1 and TM2 that lacks Cys residues; on the other hand, HAP2 homologs are characterized by having a single TM, followed by a cytoplasmic tail that often contains Cys implicated in fusion^64^. **b** Crystal structure of the ectodomain of Fsx1 and predicted topology of the full-length protein relative to the plasma membrane. Domains I–IV are shown in red, yellow, blue, and green, respectively; disulfide bonds are indicated and colored magenta. **c** Topology diagrams of the ectodomains of Fsx1, *C. reinhardtii* HAP2 (PDB 6E18 [https://www.rcsb.org/structure/6E18])^65^, *A. thaliana* HAP2 (PDB 5OW3 [https://www.rcsb.org/structure/5OW3])^25^, and *T. cruzi* HAP2 (PDB 5OW4 [https://www.rcsb.org/structure/5OW4])^25^. Domains and disulfide bonds are colored as in panel **b**. Note how, although domain II of Fsx1 has the same topology as the corresponding domain of HAP2, it contains only one of its conserved disulfide bonds (C_1_125–C_2_155, corresponding to C147-C210/disulfide bond 3 of CrHAP2^23^). **d** Side-by-side comparison of Fsx1_E_ and known class II fusogens. Elements are colored as in panel **b**; the stem region and the linker between domains I and III are shown in pink and cyan, respectively.

**Fig. 3:**
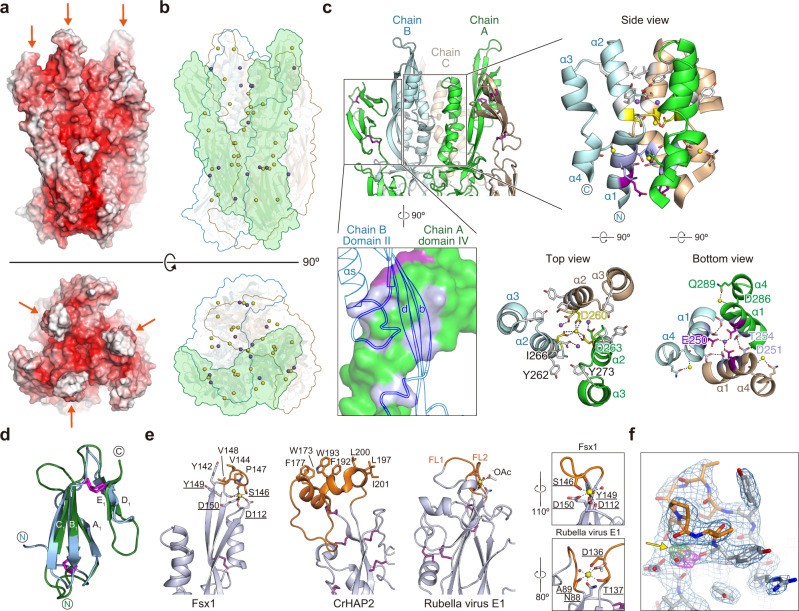
Distinct structural features of Fsx1. **a** Fsx1_E_ surface colored by electrostatic potential from red (−10 kT/e) to blue (+10 kT/e) through white (0 kT/e). Orange arrows indicate the FLs. **b** Location of the Ca^2+^ and Na^+^ ions (depicted as yellow and purple spheres, respectively; see also Supplementary Figs. 4 and 5) stabilizing the Fsx1_E_ trimer. The molecular surface of a protomer is shaded green and the outline of the other two subunits is colored cyan and wheat. **c** Details of interactions at the level of the six-helical bundle made by domain II of the Fsx1 subunits (right subpanels) and the domain IV/domain II interface (bottom left subpanel). Selected side chains are colored by the type of inter-chain contacts in which they are involved (gray: hydrophobic interaction; blue bell: hydrogen bonding; yellow: Ca^2+^ coordination; fuchsia: Na^+^ coordination), with dashed lines indicating hydrogen bonds. Note that the helical bundle of Fsx1 is not a leucine-zipper coiled-coil structure, such as those found in class I/III viral fusion proteins or in the SNARE four-helix bundles, and see also Supplementary Fig. 5b. **d** Superposition of Fsx1 domain IV (green) and Der p 23 (PDB 4ZCE [https://www.rcsb.org/structure/4ZCE]^31^, blue) (Dali *Z*-score 3.6, RMSD 2.2 Å). **e** Comparison of the Fsx1 region that includes the FL and the corresponding parts of CrHAP2 and Rubella virus E1 protein (PDB 4B3V [https://www.rcsb.org/structure/4B3V]^33^). Residues coordinating the Ca^2+^ ion that stabilizes the Fsx1 FL are underlined, and compared to the E1 protein Ca^2+^-binding region in the boxed panels on the far right. **f** The Fsx1 FL adopts a highly ordered conformation stabilized by a Ca^2+^ ion. Presence and identity of the latter, indicated by a yellow arrow, are supported by two other maps shown in addition to the *2mFo-DFc* map (blue mesh, contoured at 1.0 *σ*): a difference map calculated upon omitting all metal ions from the model (thick green(+)/red(−) mesh, 6.0 *σ*) and a phased anomalous difference map calculated from a 2.9 Å-resolution dataset collected at 7.1 keV (thick magenta mesh, 3.2 *σ*).

The Fsx1_E_ homotrimer has overall dimensions of 119 × 77 × 76 Å (Fig. 1b). Each protomer consists of four domains (Fig. 2a, b), the first three of which match the approximate dimensions and relative arrangement of domains I–III of known fusexins in their post-fusion conformation^30^; accordingly, fold and interface similarity searches identify HAP2 as the closest structural homolog of Fsx1_E_, followed by viral fusexins and *C. elegans* EFF-1 (Fig. 2c, d). Fsx1 domains I and III are relatively sequence-conserved among archaeal homologs (Supplementary Figs. 5a and 6) and closely resemble the corresponding domains of HAP2 (RMSD 2.1 Å over 218 Cα), including the invariant disulfide bond between domain III strands βC and βF^23^ (C_3_389–C_4_432; Fig. 2, Supplementary Fig. 4c). On the other hand, Fsx1 domain II shares the same topology as that of HAP2 but differs significantly in its secondary structure elements and their relative orientation, as well as disulfide bonds (Fig. 2c). In particular, Fsx1 domain II is characterized by a four-helix hairpin, whose N-terminal half interacts with the same region of the other two subunits to generate a six-helix bundle around the molecule’s three-fold axis (Figs. 1b and 3a–c and Supplementary Figs. 4a and 5b, c).

Notably, unlike previously characterized viral and eukaryotic fusexins, Fsx1 also contains a fourth globular domain conserved among archaeal homologs (Figs. 1b, 2 and Supplementary Figs. 4d and 6). Its antiparallel β-sandwich, which includes the two C-terminal disulfides of Fsx1, resembles the carbohydrate-binding fold of dust mite allergen Der p 23 and related chitin-binding proteins^31^ (Fig. 3d); accordingly, it is also structurally similar to a high-confidence AlphaFold2 model of the C-terminal domain of acidic mammalian chitinase^32^. In addition to being coaxially stacked with domain III as a result of a loop/loop interaction stabilized by the C_5_457–C_6_477 disulfide, domain IV contributes to the quaternary structure of the protein by interacting with domain II of the adjacent subunit to which domain III also binds (Figs. 1b and 3c).

The Fsx1_E_ monomer has a net charge of −67, and another feature stabilizing its homotrimeric assembly is a set of Ca^2+^ and Na^+^ ions that interacts with negatively charged residues at the interface between subunits (Fig. 3a–c and Supplementary Figs. 4a and 5b). Additional metal ions bind to sites located within individual subunits; in particular, a Ca^2+^ ion shapes the conformation of the domain II cd loop (S143-V148) so that its uncharged surface protrudes from the rest of the molecule (Fig. 3e, f and Supplementary Fig. 5d). Strikingly, the position of this element matches that of the fusion loops (FLs) of other fusexins, including the Ca^2+^-binding fusion surface of rubella virus E1 protein^33,34^ (Fig. 3e). Moreover, as previously observed in the case of CrHAP2^26^, the loops of each trimer interact with those of another trimer within the Fsx1 crystal lattice.

In summary, despite significant differences in the fold of domain II, the unprecedented presence of a domain IV and extreme electrostatic properties, the overall structural similarity between Fsx1 and viral or eukaryotic fusexins suggests that this prokaryotic molecule also functions to fuse membranes.

### Fsx1 can fuse eukaryotic cells

**Fig. 4:**
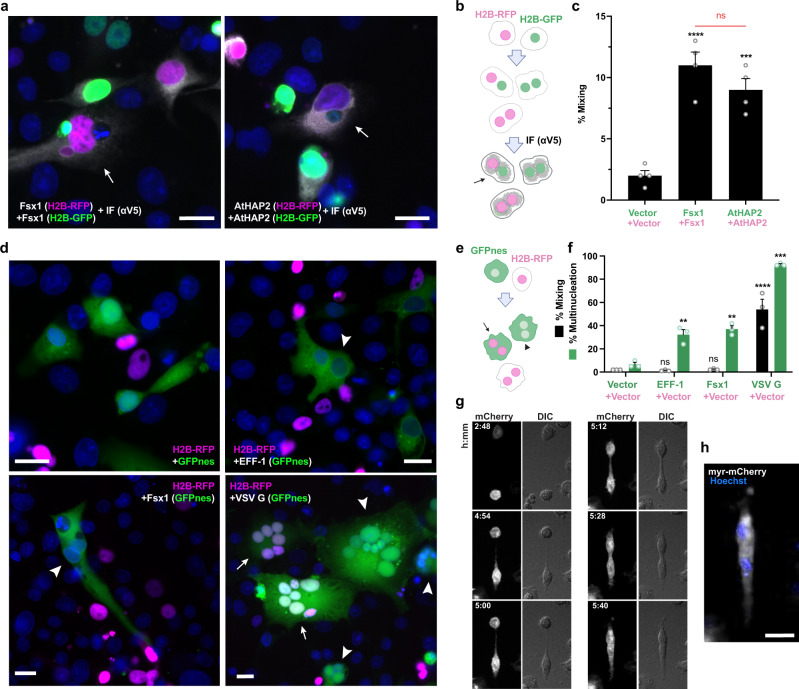
Fsx1 mediates bilateral cell–cell fusion. **a**–**c** Cell–cell fusion was measured by content-mixing, indicated by the appearance of multinucleated cells containing green nuclei (H2B-GFP) and magenta nuclei (H2B-RFP). Immunofluorescence against the V5 tag was also performed (gray). **a** Images of mixed cells. DAPI, blue. Scale bars, 20 µm. See also Supplementary Fig. 7a. **b** Scheme of experimental design. **c** Quantification of content-mixing. The mixing indices presented as means ± SEM of four independent experiments. Comparisons by one-way ANOVA followed by Bonferroni’s test. ns = non-significant, ***p < 0.001, ****p < 0.0001. Source data are provided as a Source Data file. **d**–**f** Unilateral fusion was evaluated by mixing control cells expressing nuclear H2B-RFP (magenta) with cells expressing GFP with a nuclear export signal (GFPnes, green cytoplasm) only or together with Fsx1, EFF-1 or VSV G. **d** Images of cells transfected with empty::GFPnes vector, Fsx1::GFPnes, EFF-1::GFPnes or VSV G::GFPnes. Fsx1 and EFF-1 show multinucleated GFPnes positive cells (arrowheads). VSV G multinucleated cells are found with GFP only (arrowheads) or with both markers (arrows). Scale bars, 20 µm. See also Supplementary Fig. 7e. **e** Scheme of experimental design. **f** Quantification of content-mixing experiments in which only the GFP population of cells express Fsx1, EFF-1, VSV G, or none of them (vector). Bar chart showing means ± SEM of three independent experiments. Comparisons by one-way ANOVA followed by Dunett’s test against the vector. ns = non-significant, **p < 0.01, ***p < 0.001, ****p < 0.0001. Source data are provided as a Source Data file. **g** Spinning disk microscopy time-lapse images indicating the merging of two cells expressing myr-mCherry and Fsx1. Time in hours:minutes. The red channel (mCherry, white) and the DIC are shown. Refer to Supplementary Movie 1. **h** For the last point a Z-projection showing the myr-mCherry fluorescence (white) and the nuclei Hoechst (blue; Supplementary Movie 2). Scale bar, 20 µm.

To test the fusogenic activities of the candidate archaeal fusexins we studied their fusion activity upon transfection in eukaryotic cells^15,16,22^. Cells with either red or green nuclei are mixed with each other and fusion is measured by the formation of hybrid cells with both red and green nuclei revealing merger of their cytoplasms. For this, we co-cultured two batches of Baby Hamster Kidney (BHK) cells independently transfected with Fsx1 and co-expressing either nuclear H2B-RFP or H2B-GFP^22^. We then performed immunofluorescence against a V5 tag fused to the cytoplasmic tail of Fsx1 (Fig. 4a, b, and Supplementary Fig. 7a, b). We observed a five-fold increase in the mixing of the nuclear H2B-GFP and H2B-RFP compared to vector control, showing that Fsx1 is a bona fide fusogen, as efficient as the fusexin AtHAP2 (Fig. 4c). To determine whether Fsx1 expression is required in both fusing cells or, alternatively, it suffices in one of the fusing partners, we mixed BHK-Fsx1 coexpressing cytoplasmic GFP with BHK cells expressing only nuclear RFP. We found increased multinucleation of GFP+ cells (revealing cell–cell fusion) but very low mixing with RFP+ cells not expressing Fsx1. In contrast, the vesicular stomatitis virus G-glycoprotein (VSVG) fusogen-induced efficient unilateral fusion^15^ (Fig. 4d–f). While VSVG requires acidic pH for maximum fusion activity Fsx1-mediated multinucleation was not stimulated by low pH (Supplementary Fig. 1e). Thus, Fsx1 acts in a bilateral way, similarly to EFF-1 and AFF-1 fusexins^14,15,35^. We then performed live imaging using spinning disk confocal microscopy and observed bilateral cell-cell fusion of BHK-Fsx1 cells (Fig. 4g, h).

### Structure–function analysis of Fsx1

To compare archaeal Fsx1 activity with fusexins from eukaryotes and viruses, we introduced mutations into three different structural domains of Fsx1 and tested surface expression and fusogenic activity in mammalian cells.

**Fig. 5:**
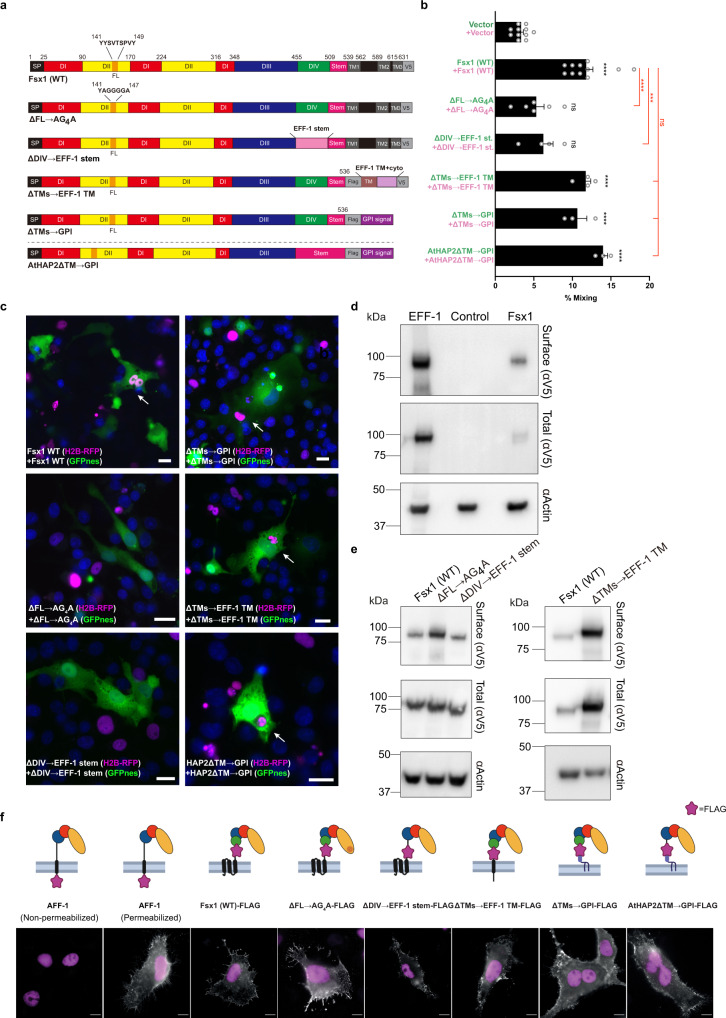
Structure–function analysis of Fsx1. **a** Schematic diagram of wild-type Fsx1, four mutants and AtHAP2ΔTM → GPI. SP signal peptide, FL fusion loop. For colors and abbreviations see legend of Fig. 2. **b** Quantification of content-mixing (cell–cell fusion) in populations of cells expressing vectors (*n* = 7), Fsx1 (wt) (*n* = 7), its mutants (ΔFL → AG_4_A (*n* = 6), ΔDIV → EFF-1 stem (*n* = 4), ΔTMs → EFF-1 TM (*n* = 4), ΔTMs → GPI (*n* = 3), or AtHAP2ΔTM → GPI (*n* = 3). Bar chart showing means ± SEM. Comparisons by one-way ANOVA followed by Bonferroni’s test against the vector (black) and against Fsx1 (red). ns = non-significant, ***p < 0.001, ****p < 0.0001. Source data are provided as a Source Data file. **c** Representative merged images from the experiments in (**b**): magenta (RFP); green (GFP) and blue (DAPI). Fused cells with RFP and GFP (arrows). Scale bars, 20 µm. See also Supplementary Fig. 7f. **d** Immunoblot of EFF-1-V5, control (untransfected cells) and Fsx1-V5 expressing cells. “Surface” indicates surface biotinylation followed by affinity purification using neutravidin agarose beads; “Total” indicates the expression in whole cell extracts. Actin is used as a loading control. The amount of initial cells for Fsx1 is 4 times higher than EFF-1. *n* = 3. **e** Surface biotinylation as explained in panel **d** for cells expressing Fsx1-V5 (WT), ΔFL → AG_4_A-V5, ΔDIV → EFF-1 stem-V5 or ΔTMs→EFF-1 TM-V5. *n* = 3. **f** Immunofluorescence images on non-permeabilized cells expressing Fsx1-FLAG (WT), AFF-1-FLAG (negative control, cytotail), Fsx1-ΔFL → AG_4_A-FLAG, Fsx1-ΔDIV → EFF-1 stem-FLAG, AFF-1-FLAG (permeabilized), Fsx1-ΔTMs → EFF-1 TM-FLAG, Fsx1-ΔTMs → GPI and AtHAP2-ΔTM → GPI. The FLAG tag was inserted before the first TM or the GPI signal of each construct except for *C. elegans* AFF-1 in which the FLAG is at C-terminal after the cytoplasmic tail. Transfected BHK cells were incubated with anti-FLAG antibody on ice before fixation. Non-permeabilized staining of FLAG antibody showed the surface expression of Fsx1 and the mutants. *C. elegans* AFF-1 tagged with a cytoplasmic FLAG is a negative control for non-permeabilized staining. Permeabilized staining of CeAFF-1-FLAG shows the localization on plasma membrane and internal compartments (see also Supplementary Fig. 7g). Scale bars, 10 µm.

First, to test whether the putative FL of Fsx1 (143-SVTSPV-148) is involved in fusion, we replaced it with a linker of 4G between Y142A and Y149A (Figs. 3e, 5a, and Supplementary Fig. 5d; ΔFL → AG_4_A). This replacement does not affect surface expression yet reduces cell–cell fusion to levels similar to those of the negative control (Fig. 5b–f).

Second, we asked whether domain IV, which is only present in archaeal fusexins, has a function in the fusion process. For this, we replaced the entire domain with the stem region of EFF-1 (Figs. 3d and 5a; ΔDIV → EFF-1 stem). While this mutant Fsx1 reaches the cell surface, suggesting that it folds normally, it shows a significantly reduced activity compared to wild-type Fsx1 (Fig. 5b–f).

Third, to test whether the three TMs of Fsx1 are required for fusion, we replaced them with the TM and cytoplasmic domains of EFF-1 (Fig. 5a; ΔTMs → EFF-1 TM) or a glycosylphosphatidylinositol (GPI) anchor signal (Fig. 5a; ΔTMs → GPI). We found that both Fsx1 mutants remained active (Fig. 5b), indicating that the Fsx1 TMs are not essential for fusion. Finally, we also replaced the TM and cytoplasmic domains of AtHAP2 with a signal for GPI and found that the protein also maintained its fusogenic activity (Figs. 4c, and 5a, b). Thus, contrary to some viral fusogens in which the GPI-anchored glycoproteins fail to drive complete fusion^36–38^, lipid-anchored Fsx1 or eukaryotic HAP2 promote syncytia formation when expressed on the surface of BHK cells.

### Fsx1s are ancient fusogens associated with integrated mobile elements

**Fig. 6:**
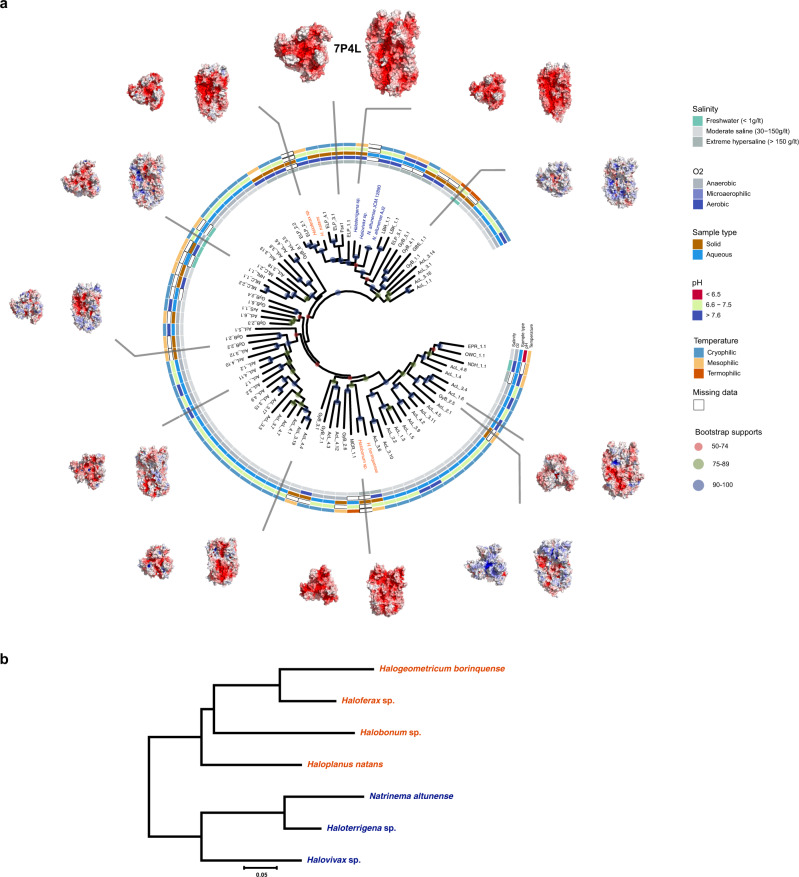
Environmental distribution of Fsx1s. **a** Archaeal fusexins unrooted phylogeny, environmental details, and trimeric models^66^ based on the Fsx1 X-ray structure (PDB 7P4L [https://www.rcsb.org/structure/7P4L], enlarged). Tree tip IDs coming from metagenomic data coded by sampling site, sample id and sequence (see Supplementary Data 1). Fsx1s from cultivated genomes are shown as Natrialbales and Haloferacales orders in blue and red, respectively. Surfaces colored and oriented as in Fig. 3a. Found in diverse environments and often differing in global surface properties, modeled trimers share hydrophobic tips. **b** Panel with phylogenomic tree for *fsx1*-containing cultured archaeal species, showing incongruences between Fsx1s in Haloferacales.

**Fig. 7:**
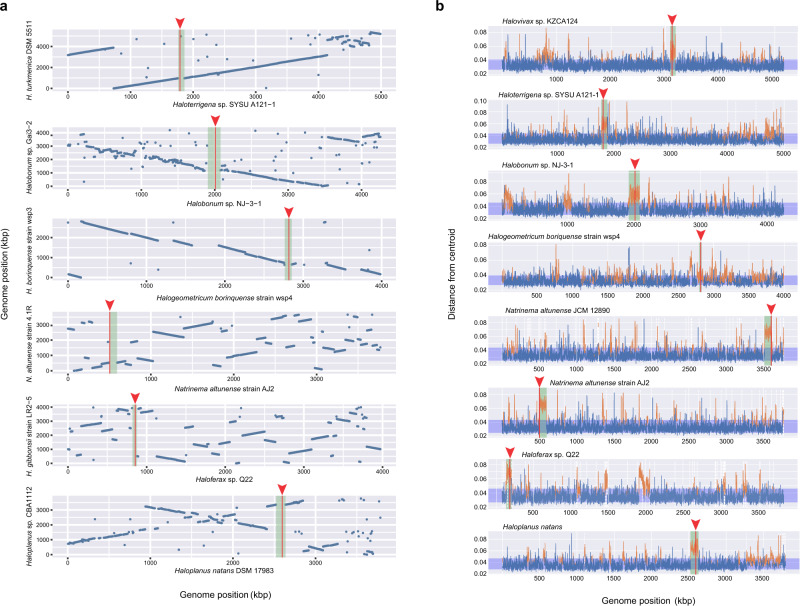
Genomic features of Fsx1s. **a** Whole genome comparison of species with and without *fsx1*. Each blue dot represents a segment of 500 bp with more than 80% identity between the species harboring *fsx1* (e.g., *Haloplanus natans* DSM 17083) and the species with no *fsx1* (e.g., *Haloplanus* sp. CBA1112, see Supplementary Table 4). Species with *fsx1* are in the *x*-axis, the base of the green rectangles represent the detected IME carrying the *fsx1* gene, locus of *fsx1* is in red vertical line and pointed with a red arrowhead. **b** K-mer spectra deviation of *fsx1*-containing IMEs. K-mer spectrum deviation from centroid is shown for each of the Pure Culture Genomes (PCGs) where *fsx1* was detected. Blue region shows the standard deviation. Locus of *fsx1* is in red vertical line and pointed with a red arrowhead, the mobile element containing *fsx1* is in green. Dashed vertical white lines indicate the end of a contig in the genome assembly. *fsx1* is consistently found within regions that deviate from the core genome’s spectrum, indicating they belong to a mobile element. Kbp, kilo base pairs.

**Fig. 8:**
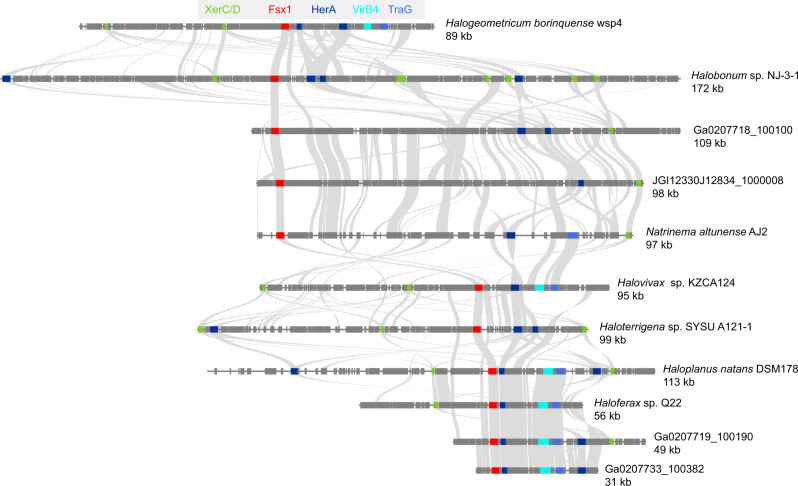
Fsx1s are embedded in integrated mobile elements (IMEs). Synteny plots for IMEs from PCGs and metagenomic data. Annotated regions plus inferred ORFs belonging to homologous clusters identified by the workflow depicted in Supplementary Fig. 8. Homology relationships are represented by gray links. *fsx1* genes are marked in red and selected ORFs homologous to IME signature genes are labeled and color-coded. XerC/XerD recombinases (green); HerA helicase (dark blue); VirB4, Type IV secretion system (T4SS) pathway (cyan); TraG/TraD/VirD4 family enzyme, ATPase, T4SS (see Supplementary Table 3 and Supplementary Data 2, 3 for details). The 11 segments analyzed correspond to the cluster marked in Supplementary Fig. 9.

The *fsx1* genes here identified are present in a wide physicochemical landscape (Fig. 6a). We observed that the branching pattern of Fsx1 sequences from complete genomes is incompatible with their species tree (Fig. 6a, b). This and the sparse pattern of Fsx1 presence in Archaea led us to perform genomic comparisons of related species with and without the *fsx1* gene. These revealed >50 kbp DNA insertions in the genomes of species with *fsx1* genes (Fig. 7a). To investigate them, we performed k-mer spectrum analysis on *fsx1*-containing Pure Culture Genomes (PCGs) and found divergent regions containing the *fsx1* ORF (Fig. 7b). Gene content analyses of *fsx1*-containing regions show that they share a portion of their genes (Supplementary Fig. 9) and display conserved synteny (Fig. 8), suggesting common ancestry. These regions are enriched in ORFs homologous to proteins involved in DNA mobilization and integration (Fig. 8 and Supplementary Table 3). Thus, our results indicate that *fsx1* genes are contained in integrated mobile elements (IMEs) that can be mobilized by a conjugative-like, cell fusion-dependent mechanism.

To describe Fsx1’s tempo and mode of evolution we first compared archaeal and sexual fusexins, which share enough sequence conservation to apply standard phylogenetic analyses, not possible for somatic and viral fusexins, as these methods are not able to cope with such amount of divergence (Fig. 1a and Supplementary Fig. 1a). We built maximum likelihood (ML) phylogenies for a set of Fsx1 sequences derived from isolated species and metagenomes, and a subset of HAP2s which capture the full phylogenetic diversity present in eukaryotic lineages (Supplementary Fig. 10a). A major finding comes from these phylogenies: eukaryal and archaeal fusexins cluster into strongly supported clades suggesting they diverged before LECA.

To place *fsx1* in the broader fusexin superfamily context, we performed structural phylogenetic analysis comparing crystal structures from viral, somatic, and eukaryotic gamete fusogens (Supplementary Fig. 10b). This structure-based tree supports a viral origin of somatic fusexins (EFF-1)^16^ and is also compatible with archaeal fusexins appearing before the radiation of eukaryotes.

## Discussion

All *fsx1* genes found in cultured and isolated genomes are restricted to the Haloarchaea clade. Although 83% of *fsx1* genes were found in saline environments, they are not restricted to a particular niche, neither geographically nor environmentally and, by extension, potentially not restricted to halophilic archaea (Supplementary Data 1 and Fig. 6a). Only 16 out of 88 *fsx1* metagenomic genes have taxonomy assigned. The 16 MAG-containing fusexins are distributed in other archaeal clades (including Asgard) and also in bacteria but we take those taxonomic assignments with caution. *fsx1*-containing MAGs are highly fragmented and were assembled using methods that include sequence composition (k-mer) criteria, allocating scaffolds with similar k-mer spectra into MAGs. As *fsx1* genes from PCGs are located within distinctive k-mer regions and the metagenomic contigs containing the *fsx1* gene are compositionally homogeneous, and plausibly are also IMEs, it is quite possible that MAG-fusexins are misassigned. The association of haloarchaeal *fsx1s* with IMEs (Fig. 8), genomic comparisons of close species (Fig. 7a) and the incompatibility between their sequence phylogeny and cognate species tree (Fig. 6), indicate lateral mobility within the Haloarchaea class. This evidence suggests that Fsx1s mediate a cell fusion-dependent genetic exchange in archaea. This hypothesis is consistent with the genetic structure and lifestyle of halophilic archaea which are notorious for being polyploid^39^ and undergoing massive genetic exchanges that overcome species and genera barriers^40,41^. Moreover, compelling evidence of archaeal cell fusion comes from studies showing bilateral DNA exchange that correlates with cytoplasmic bridges made up of fused lipid bilayers connecting haloarchaeal cells^42–44^. Thus, it is plausible that Haloarchaea evolved HGT mechanisms based on conjugative-like DNA mobilization and cell–cell fusion^45^.

**Fig. 9:**
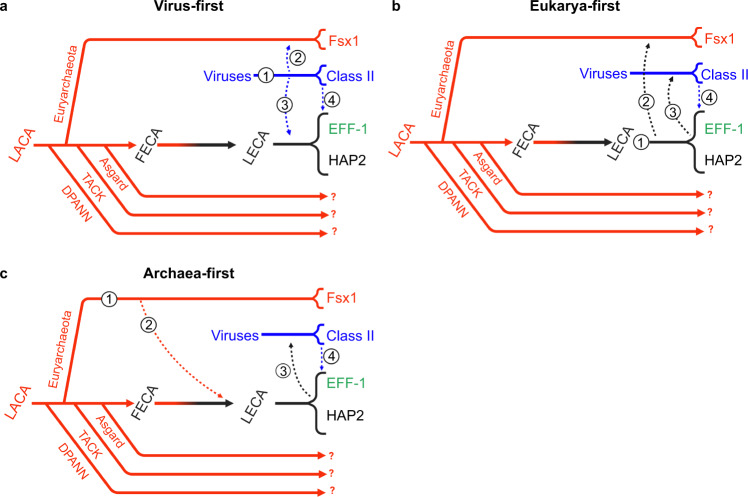
Possible evolutionary trajectories. **a** Virus-first. Originating in the virosphere (1), ancestral fusexins were transfered to an ancient eukariotic organism (2) before the eukaryal radiation. Also before the eukaryal radiation, either viral (3) or early eukaryal (not numbered) fusexins were horizontally transferred to Archaea where they became integrated mobile elements. **b** Eukarya-first. Originating before the eukaryal radiation (1), fusexins were horizontally transferred to Archaea (2) where they became integrated in mobile elements. Eukaryal fusexins were captured by eukaryal viruses (3) leading to extant viral (class II) fusexins. **c** Archaea-first. Originating in Euryarchaeota (1), fusexins were horizontally transfered (2) and became fixed during eukaryogenesis and the emergence of meiotic sex. Viral capture of fusexin genes from early eukaryotic cells and further evolution within the virosphere led to extant viral (class II) fusexins (3). Common to all models, viral fusexin genes were captured by different eukaryotic lineages (4), leading to phlebovirus-like integrated fusogens and EFF-1 somatic fusogens^48,67^. Solid lines represent evolutionary trajectories of Archaea (red), viruses (blue), Eukarya (black), and eukaryogenesis (red to black gradient). Dashed arrows represent HGT events. Question marks denote uncertainty regarding the presence of *fsx1*-related genes in the respective lineages. LACA Last Archaeal Common Ancestor.

The “virus or the egg” dilemma^28^ posits that fusexins may have been either viral innovations (class II fusogens), later acquired by eukaryotes, or vice versa. Archaeal fusexins expand this dilemma: gamete fusogens may have prokaryotic origins. Both structure- and sequence-based trees (Supplementary Fig. 10) do not solve but provide insights to address this conundrum, in which we distinguish three main hypotheses indicating alternative origins: Virus, Eukarya, and Archaea (Fig. 9). For all three scenarios we assume that sexual fusexins (HAP2) were present in the LECA^1,27^.

A Virus-first scenario has circumstantial evidence favoring it. Exaptation of viral genes is documented for all three domains of life^46^. To be consistent with the observed basal divergence between archaeal and eukaryal (HAP2) fusexins (Supplementary Fig. 10a) the Virus-first hypothesis must include two HGT events before the eukaryal radiation (Fig. 9a). Thus, an archaeon could have exapted a fusexin from an enveloped archaeal virus and then transferred it to an early eukaryote. The reciprocal is also formally possible: an eukaryal viral fusexin was exapted by a pre-LECA eukaryotic cell and then transferred to an archaeon, before the eukaryal radiation. Alternatively, the ancestral fusexin-containing virus was able to infect both archaea and eukaryal cells (Fig. 9a). These putative events are at odds with the distribution of fusexins in extant viruses. All currently known viral fusexins belong to RNA viruses that are confined to a few multicellular hosts: vertebrates, arthropods, and flowering plants^47,48^. This distribution favors a scenario where viral fusexins, like many other eukaryotic viral proteins, have eukaryotic cellular origins^49^. Unlike currently known fusexin-containing viruses, all isolated archaeal viruses to date have DNA genomes. The recently elucidated structure of VP5, a haloarchaeal virus envelope protein that mediates cell invasion shows a fold that differs from all previously known viral fusion proteins, including fusexins^50^. Although there is no evidence for the presence of fusexins in archaeal viruses, upcoming metagenomic and structural analyses may provide support to the Virus-first hypothesis.

The widespread presence of sexual fusexins in Eukarya indicates evolutionary success, in line with the Eukarya-first hypothesis (Fig. 9b). However, introduction of an eukaryal fusexin into the Archaeal domain is less supported by currently available evidence as interdomain gene transfers from eukaryotes to archaea are hardly documented and thought to be scarce^45,51^.

The presence of *fsx1* genes in Haloarchaea IMEs is consistent with gene transfer in the opposite direction. Eukaryogenesis, and by extension the emergence of sex, is marked by massive horizontal gene transfer events to the archaeal ancestor of eukaryotes^52^, disregarding if it belonged to the Asgard superphylum or to a sister group of Archaea^53^. During the First Eukaryotic Common Ancestor (FECA) to LECA transition, in addition to the alphaproteobacterial endosymbiont-related inherited genes, the pre-LECA received hundreds of archaeal genes from other lineages, including Euryarchaeota^54^. Thus, an Archaea-first hypothesis (Fig. 9c) considers horizontal transfer of archaeal fusexins into the ancestor of eukaryotes. A weakness of this hypothesis is the sparse distribution of fusexins in archaeal genomes. This distribution is not a product of a recent HGT from Eukarya, as phylogenetic analysis indicates basal divergence between eukaryotic and archaeal fusexins (Supplementary Fig. 10a). Lateral mobility of extant *fsx1* genes within Haloarchaea, their relative confinement to few archaeal lineages and their basal divergence from sexual fusexins suggests they are molecular relics, and that cell fusion-based HGT might have declined during archaeal evolution in favor of conjugation, transduction and transformation.

This third scenario, an Archaeal origin of fusexins, poses new challenges to both sex origins and eukaryogenesis models. Discovery of the Asgard superphylum^55^ and the recent cultivation of one of its members^56^ support eukaryogenesis scenarios where populations of bacteria and archaea lived in syntrophy, transferring metabolites and genes^57^. Acquisition of a *fsx1* gene during the FECA to LECA transition could have enabled pre-LECA cells to undergo genome expansion, explore syncytial forms^58^ and evolve into mononucleated cells fully equipped for meiosis and gamete fusion^59^. Our findings raise the possibility that gamete fusion is the product of over two billion years of evolution of this ancient archaeal cell fusion machine.

The archaeal proteins herein identified place fusexins in yet another domain of life, with different membrane chemistries and along a broad niche landscape, from cold hypersaline lakes to hot springs and hydrothermal vents (Fig. 6a). Our structural and functional analyses show that Fsx1 has both conserved and divergent properties when compared to eukaryotic and viral fusexins (Figs. 3 and 4). Like its viral counterparts, Fsx1 has an uncharged loop required for fusion. However, unlike previously known fusexins, Fsx1 harbors an additional domain (IV) involved in fusogenic activity that may bind sugars (Figs. 2d and 3d). Considering that cell surface glycosylation was found to be important for fusion-based mating of halophilic archaea^60^, this domain may actively promote fusion by interacting with carbohydrates attached to lipids or proteins such as S-layer glycoproteins^42^. Unlike HAP2s, Fsx1 homologs have 1–4 TMs and a variable Cys number (5–30, see Supplementary Data 1). Like eukaryotic fusexins, Fsx1 mediates BHK cell fusion in a bilateral fashion (Fig. 4f). However, in contrast to viral fusogens^36–38,61,62^, the fusion activity of Fsx1 is maintained following substitution of its three TMs with a single TM or a GPI anchor. The retention of fusion activity when the transmembrane domains are replaced by a GPI anchor supports the model in which Fsx1 mediates homotypic fusion with fusogens required on both target membranes. These findings also suggest that interaction between the TMs during fusion is not essential for trimerization and expansion of the fusion pores. Since GPI-anchored AtHAP2 is also fusogenic, other fusexins may also drive complete cell fusion without a specific involvement of TMs. Future studies will address the function of the six-helix bundle formed by Fsx1 domain II, which is unprecedented among fusexins and raises an unexpected structural connection with class I viral fusogens^5,6^.

What are the limitations of this study? First, the description of the cellular and molecular functions of the *fsx1* family presented in this work is reliant upon expression within a heterologous system designed to probe fusion activity. Despite membrane chemistry differing from the type that would be associated with haloarchaeal hosts of the IME, Fsx1 was able to promote membrane fusion reliably. Second, evolutionary analysis and comparative genomics confirm *fsx1*’s link to IMEs. These sequences appear to be spread across a wide variety of niches all across the globe despite a relatively sparse distribution in sequenced archaeal genomes. Third, in this study we focused on describing the Fsx1 family evolutionarily, structurally and functionally to place it within its context in the Fusexin superfamily, but future studies will be needed to elucidate which biological processes *fsx1* is involved in as well as its relationship to the rest of the archaeal mobilome and virome. Additionally, structural features, such as the presence of a fourth domain, were modified in order to observe their effect on fusion activity within our experimental system, but it remains to be seen what importance they have in their native context. Future work will focus on studying *fsx1* in archaeal experimental systems as well as leverage metagenomic sampling and assembly techniques to exhaustively detect possible *fsx1* homologs in environments where it may be facilitating horizontal gene transfer.

## Methods

### Initial fusexin search using structurally guided MSAs

HMMs were prepared using structurally guided multiple sequence alignments (MSAs) of known eukaryotic HAP2 sequences (ectodomains only). Structural MSAs were derived using I-TASSER^68^-generated models of HAP2 homologs for *Erythranthe guttata* (A0A022QRC8), *Phytomonas* sp. isolate EM1 (W6KUI1), *Plasmodium falciparum* (A0A1C3KGX6), *Chlorella variabilis* (E1Z455) and the HAP2 crystal structures for *Chlamydomonas reinhardtii* (PDB 6E18 [https://www.rcsb.org/structure/6E18]^65^, 6DBS [https://www.rcsb.org/structure/6DBS]^26^) and *Arabidopsis thaliana* (PDB 5OW3 [https://www.rcsb.org/structure/5OW3]^25^).

Searches for fusexin homologs using structurally guided MSAs were performed for 3 iterations on the Uniclust database^69^ using default HHblits^70^ parameters.

### HMM-based distance matrices

A taxonomically representative list of known viral and eukaryotic fusexin homologs, covering major lineages, was manually curated. A MSA was built for each homolog by using the sequence as a query on the Uniclust database with HHblits for three iterations. This set of MSAs was compiled into an HH-suite database and each MSA was used as a query against this database to establish a profile-based distance matrix using the probability of homology (Fig. 1a and Supplementary Fig. 1).

### Metaclust database search pipeline

We searched the Metaclust^71^ dataset (nr50) using an HMM made of Fsx1 sequences found in PCGs and MAGs (Supplementary Data 1; see also codes, notebooks and datasets available at Zenodo^63^). Fsx1 sequences were aligned using ClustalO^72^ with default settings for 3 iterations and the resulting MSA was used as a query with HMMER hmmsearch^73^ against the Metaclust50 dataset^71^. All returned sequences with an *E*-value < 0.0001 with a match length greater than 100 residues were selected for further analysis. PSI-BLAST^74^ was also used on the Metaclust (nr90) with Fsx1 sequences found in PCGs and MAGs with default parameters for 3 iterations. All returned sequences with an *E*-value < 0.0001 and an alignment length greater than 100 were added to the pool of candidates. Manual curation was performed using membrane protein topology predictor TOPCONS^75^ and distant homology searches using HHblits^76^ against PDB70.

### DNA constructs

Ten archaeal genes were synthesized (GenScript) and cloned into pGene/V5-His vectors (Supplementary Table 5). Details of nucleotides used for synthesis and protein sequences are described in Supplementary Data 4.

For structural studies, a synthetic gene fragment encoding the extracellular region of a metagenomic Fsx1 ORF (IMG genome 3300000868, scaffold JGI12330J12834_ 1000008, ORF 8; Supplementary Data 1) (GenScript) was subcloned by PCR in frame with the 5′ chicken Crypα signal peptide- and 3′ 8xHis-tag-encoding sequences of pLJ6, a mammalian expression vector derived from pHLsec3^77^. The protein construct that yielded the final high-resolution dataset included residues D25-S535 and contained a T369C substitution, introduced by PCR mutagenesis with the aim of facilitating heavy atom derivatization for experimental phasing. Oligonucleotides were from Sigma-Aldrich or IDT and all constructs were verified by DNA sequencing (Eurofins Genomics or Macrogen).

To generate pCI::GFPnes plasmid (see list of plasmids in Supplementary Table 6), an oligo DNA encoding for the nuclear export signal (LQKKLEELELD) was cloned downstream the region encoding EGFP of the pCAGIG plasmid using the enzyme BsrGI. Then, the GFPnes coding sequence was amplified, cut with BmgBI and BglII and used to replace the H2B-GFP coding sequence of the pCI::H2B-GFP plasmid (see list of primers in Supplementary Table 7). Fsx1-V5, AtHAP2-V5^22^, EFF-1-V5, VSV-G^15^ and other archaeal fusexins (NaFsx1, HQ22Fsx1, HnFsx1) were subcloned into corresponding pCI::H2B-RFP/H2B-GFP/GFPnes vectors separately. For mutagenesis of Fsx1, (i) Fsx1-ΔFL-AG_4_A: The mutation of Y142A, Y149A and four glycines inserted between them were achieved using PCR with overlapping primers. (ii) Fsx1-ΔDIV-EFF-1 stem: The stem region of EFF-1 (E510-D561) was amplified from pGene::EFF-1-V5 and fused to the upstream and downstream regions of Fsx1-DIV with overlapping primers. (iii) Fsx1ΔTMs → EFF-1 TM: The TM and cytoplasmic tail of EFF-1 (I562–I658) were amplified from pGene::EFF-1-V5 and fused to the ectodomain of Fsx1 to replace its TMs. (iv) Fsx1ΔTMs→GPI: The Fsx1 TMs were replaced with the carboxy-terminal 37 amino acids of decay accelerating factor (DAF) which confer the signal for GPI anchor^78^. Similarly, the TM and cytoplasmic tail of AtHAP2 were replaced with the GPI signal from DAF to get AtHAP2ΔTM → GPI. All mutants were ligated into pCI::H2B-RFP and pCI::GFPnes vectors for mixing assay. Additional details are found in Supplementary Tables 6 and 7.

### Protein expression and purification

HEK293T cells (ATCC CRL-3216)^79^ were transiently transfected using 25 kDa branched polyethyleneimine and cultured in DMEM media (Invitrogen) supplemented with 2% (v/v) fetal bovine serum (Biological Industries). 90–96 h after transfection, the conditioned media from HEK293T cells was harvested, 0.2 µm-filtered (Pall) and adjusted to 20 mM Na–HEPES pH 7.8, 2.5 M NaCl, 5 mM imidazole. 10 ml Ni Sepharose excel beads (GE Healthcare) pre-equilibrated with immobilized metal affinity chromatography (IMAC) buffer (20 mM Na–HEPES pH 7.8, 2.5 M NaCl, 10 mM imidazole) were added to 1 l adjusted conditioned media and incubated overnight at 4 °C. After washing the beads with 100 column volumes IMAC buffer, captured Fsx1_E_ was batch-eluted with 30 mL 20 mM Na–HEPES pH 7.8, 2.5 M NaCl, 500 mM imidazole, and concentrated with 30 kDa-cutoff centrifugal filtration devices (Amicon). The material was then further purified by SEC at 4 °C, using an ÄKTAfplc chromatography system (GE Healthcare) equipped with a Superdex 200 Increase 10/300 GL column (GE Healthcare) pre-equilibrated with 20 mM Na-HEPES pH 7.8, 2.5 M NaCl. Peak fractions were pooled and concentrated to 5 mg mL^−1^ (Supplementary Fig. 2a, b).

### Size-exclusion chromatography-multiangle light scattering (SEC-MALS)

Purified Fsx1_E_ samples (120–150 µg) were measured using an Ettan LC high-performance liquid chromatography system with a UV-900 detector (Amersham Pharmacia Biotech; *λ* = 280 nm), coupled with a miniDawn Treos MALS detector (Wyatt Technology; *λ* = 658 nm) and an Optilab T-rEX dRI detector (Wyatt Technology; *λ* = 660 nm). Separation was performed at 20 °C using a Superdex 200 Increase 10/300 GL column (GE Healthcare) with a flow rate of 0.5 mL min^−1^ and mobile phases consisting of 20 mM Tris–HCl pH 8.5, 150 mM NaCl (normal salt condition) or 20 mM Tris–HCl pH 8.5, 2.0 M NaCl and 0.2 M CaCl_2_ (high salt condition) (Supplementary Fig. 2c). Data processing and weight-averaged molecular mass calculations were performed using ASTRA (Wyatt Technology). BSA (150 µg) was used as a control.

### Small-angle X-ray scattering (SAXS)

SAXS experiments were performed at beamline BM29 of the European Synchrotron Radiation Facility (ESRF)^80^, using Fsx1_E_ (4.5 mg mL^−1^) in 20 mM Na-HEPES pH 7.8, 150 mM NaCl. Sample delivery and measurements were performed using a 1 mm-thick quartz capillary, which is part of the BM29 BioSAXS automated sample changer unit^81^. Data were collected at 1 Å wavelength in 10 frames of 1 s at 20 °C, using an estimated beam size of 1 mm × 100 µm; buffer blank measurements were carried out under the same conditions, both before and after sample measurement. Data were averaged and subtracted using PRIMUS^82^ from the ATSAS package^83^, which was also used to calculate the pair-distance distribution function, as well as the radius of gyration and the Porod volume. Theoretical scattering curves for monomeric and trimeric Fsx1_E_ were calculated and compared with the experimental data using CRYSOL^84^. Ab initio envelope reconstruction was performed with DAMMIF^85^, resulting in 20 models that were superimposed and averaged with DAMAVER^86^. Chain A of the refined Fsx1_E_ model was either rigidly fitted with UCSF ChimeraX^87^ into the envelope generated by averaging all 20 independent ab initio SAXS models (Supplementary Fig. 2d, top envelope), or flexibly fit with Namdinator^88^ into the average envelopes generated from the two most abundant clusters of SAXS models (accounting for 4 and 5 of the 20 SAXS models, respectively; Supplementary Fig. 2d, middle and bottom envelopes).

### Crystallization and X-ray diffraction data collection

Two similar initial hits obtained from extensive screening using a mosquito crystallization robot (TTP Labtech) were manually optimized by setting up vapor diffusion experiments at 20 °C in 24-well plates. To grow diffraction-quality crystals, 1 µl purified Fsx1_E_ was mixed with 1 µL 23% (w/v) PEG 4000, 0.1 M Tris–HCl pH 8.5, 0.2 M CaCl_2_ and equilibrated against 1 mL of the same solution. Rhomboidal plates of Fsx1_E_ grew in 1–3 months from protein precipitate that appeared after overnight equilibration of the crystallization drops (Supplementary Fig. 2e). For data collection, specimens were freed from the precipitate by micromanipulation with MicroMounts (MiTeGen) and flash frozen in liquid nitrogen. More than a hundred crystals were screened at beamlines ID23-1 of the ESRF^89^ and I04 of Diamond Light Source, yielding datasets of highly variable quality. The final X-ray diffraction dataset at 2.3 Å resolution was collected at ESRF ID23-1.

### Data reduction and non-crystallographic symmetry analysis

Datasets were processed in space group *C*2 with XDS^90^ (Supplementary Table 2). By revealing a strong non-origin peak at chi = 120 (Supplementary Fig. 2f), self rotation functions calculated with MOLREP^91^ or POLARRFN^92^ clearly indicated the presence of three-fold non-crystallographic symmetry (NCS) within the asymmetric unit of the centered monoclinic crystals. Combined with Matthews coefficient calculations^93,94^, this strongly suggested that Fsx1_E_ crystallized as a homotrimer.

### Structure determination by molecular replacement with AlphaFold2 models

Multiple attempts to experimentally determine the structure of Fsx1_E_ using a variety of heavy atoms failed, probably because the high-salt mother liquor composition hindered heavy atom binding. Because molecular replacement (MR) with HAP2-derived homology models also failed, we phased the data by taking advantage of the recent significant advances in protein 3D structure prediction using machine learning^95^ to phase the data by MR^96^ (Supplementary Fig. 3). To do so, we used AlphaFold2^29^ (with default monomer prediction parameters) to generate five independent models of Fsx1 ectodomain residues D25-S535, with per-residue pseudo-B factors corresponding to 100-(per-residue confidence (pLDDT^29^)). These models had relative root-mean-square deviations (RMSD) of 1.4–3.3, or 0.7–1.9 Å after excluding 26 C-terminal residues predicted with low-confidence. Initial attempts to solve the structure by MR with Phaser^97^, using an ensemble including these models (further truncated to Q453, the predicted C-terminal end of domain III), yielded 4 solutions (with top Log Likelihood Gain (LLG) 188, final translation function *Z* score (TFZ) 9.6) that were retrospectively correct in terms of domain I/II placement, but completely wrong in the positioning of domain III. Because of the latter, automatic refinement of these solutions did not progress beyond *R*_free_ ~ 0.53. On the other hand, a parallel consecutive search for three copies of a domain I/II ensemble (D25-A335; RMSD 0.3–0.9 Å) followed by three copies of domain III (P350-Q453; RMSD 0.1–0.3 Å), using a model RMSD variance of 1 Å, yielded a clear single solution (LLG 876, TFZ 23.1) that could be automatically refined to initial *R* 0.45, *R*_free_ 0.46.

Remarkably, although a single copy of domain 3 corresponds to only 7% of the total scattering mass in the asymmetric unit of the Fsx1_E_ crystal, the very high accuracy of its AlphaFold2 model (reflected by *a posteriori*-calculated global RMSD and Distance Test Total Score (GDT_TS) of 0.7 Å and 97.6, respectively) allowed Phaser to also find a correct MR solution using just this part of the structure. Specifically, a consecutive search for three copies of the domain resulted in a trimeric model with LLG 275 and TFZ 15.1, which could be refined to starting *R* 0.51, *R*_free_ 0.51.

Also worth mentioning is the observation that the same domain I/II + domain III MR strategy used to phase the 2.3 Å resolution data could also be successfully applied to an initial dataset at much lower resolution (3.5 Å, with outer shell mean *I*/*σ*I 0.6 and CC_1/2_ 0.31); in this case, the Phaser LLG and TFZ values for the solution were 361 and 13.5, respectively, and initial automatic refinement of the corresponding model yielded *R* 0.44, *R*_free_ 0.48.

### Model building, refinement, and validation

The initial model of Fsx1_E_ was first automatically rebuilt using PHENIX AutoBuild^98^ (1083 residues; *R* 0.34, *R*_free_ 0.38) and then significantly improved with the machine learning-based sequence-docking method of ARP/wARP^99^, as implemented in CCP4^92^ (1390 residues; REFMAC^100^ R 0.23). The resulting set of coordinates was subsequently subjected to alternating cycles of manual rebuilding with Coot^101^/ISOLDE^102^ and refinement with phenix.refine^103^, using torsion-based NCS restraints and three Translation-Libration-Screw-rotation groups per chain. Putative identities of the metal ions were assigned based on electron density level; difference Fourier maps generated using alternative atom types; correspondence with peaks in phased anomalous difference maps, calculated with PHENIX^104^ or ANODE^105^ from data collected at low energy; and coordination properties^106^. Protein geometry was validated using MolProbity^107^ (Supplementary Table 2).

### Sequence-structure analysis

Sequence alignments were rendered with ESPript^108^ and manually annotated. Transmembrane helices were predicted using TMHMM^109^. GDT_TS scores were calculated using LGA^110^ and structural similarities were assessed with Dali^111^ and PDBeFold^112^. Secondary structure was assigned using DSSP^113^. Subunit interfaces were analyzed using PDBsum^114^, PIC^115^ and PDBePISA^116^. Molecular charge was calculated using the YASARA2 force field of YASARA Structure^117^ and electrostatic surface potential calculations were performed with PDB2PQR^118^ and APBS^119^, via the APBS Tools plugin of PyMOL (Schrödinger, LLC). Mapping of amino acid conservation onto the 3D structure of Fsx1_E_ was carried out by analyzing a sequence alignment of archaeal homologs with ConSurf^120^. Structural figures were generated with PyMOL.

### Structural modeling of trimeric Fsx1

Models of homotrimeric Fsx1 were generated using a local copy of AlphaFold-Multimer^121^, installed using the open source code and instructions available at https://github.com/deepmind/alphafold.

### Cells and reagents

Baby Hamster Kidney (BHK-21) cells (kindly obtained from Judith White, University of Virginia) were maintained in DMEM supplemented with 10% FBS (Biological Industries), 100 U/ml penicillin, 100 µg/ml streptomycin (Biological Industries), 2 mM l-glutamine (Biological Industries), 1 mM sodium pyruvate (Gibco), and 30 mM HEPES buffer, pH 7.3, at 37 °C with 5% CO_2_. Transfections were performed using Fugene HD (Promega) or jetPRIME (Polyplus) according to the manufacturer’s instructions.

### Immunofluorescence

BHK cells were grown on 24-well tissue-culture plates with glass coverslips. Permeabilized cells were fixed with 4% paraformaldehyde (EM grade, Bar Naor, Israel) in PBS, followed by incubation in 40 mM NH_4_Cl to block free aldehydes, permeabilized in 0.1% Triton X-100 in PBS and blocked in 1% FBS in PBS. After fixation, the coverslips were incubated 1 h with mouse anti-V5 antibody (Invitrogen, 1:500) and 1 h with the secondary antibody which was donkey anti-mouse coupled to Alexa Fluor 488 (Invitrogen, 1:500). Alternatively, for immunofluorescence without permeabilization, cells were blocked on ice in PBS with 1% FBS for 20 min, and then stained with Monoclonal ANTI-FLAG M2 antibody (Sigma, 1:1000) on ice for 1 h. After anti-FLAG staining, cells were washed and fixed with 4% PFA in PBS. Cells were blocked again and stained with the secondary antibody (donkey anti-mouse coupled to Alexa Fluor 488; Invitrogen) diluted 1:500 in PBS for 1 h. In all cases, nuclei were stained with 1 µg/ml DAPI. Images were captured using a Nikon Eclipse E800 with a 60X/1.40 Plan Apochromat objective and an optical zoom lens (Nikon) using a Hamamatsu ORCA-ER camera controlled by Micro-Manager software^122^ (Fig. 5f).

### Western blots

24 h post-transfection, cells were treated with lysis buffer (50 mM Tris–HCl pH 8.0, 100 mM NaCl, 5 mM EDTA, 1% Triton X-100 supplemented with chymostatin, leupeptin, antipain and pepstatin) on ice for 10 min. After centrifugation at 21,000 × *g* for 10 min at 4 °C, supernatants of lysates were mixed with reducing sample buffer (+DTT) and incubated 5 min at 95 °C. Samples were loaded on a 10% SDS–PAGE gel and transferred to PVDF membrane. After blocking, membranes were incubated with primary antibody anti-V5 mouse monoclonal antibody (1:5000; Invitrogen) or anti-actin (1:2000; MP Biomedicals) at 4 °C overnight and HRP-conjugated goat anti-mouse secondary antibody 1 h at room temperature. Membranes were imaged by the ECL detection system using FUSION-PULSE.6 (VILBER).

### Content mixing assays with immunofluorescence

BHK-21 cells at 70% confluence were transfected (using JetPrime; Polyplus at a ratio of 1:2 DNA:transfection reagent) with 1 µg pCI::Fsx1-V5::H2B-eGFP, pCI::Fsx1-V5::H2B-RFP, pCI::AtHAP2-V5::H2B-eGFP, pCI::AtHAP2-V5::H2B-RFP, respectively. Control cells were co-transfected with pCI::H2B-eGFP and pRFPnes or pCI::H2B-RFP and pRFPnes. 4 h after transfection, the cells were washed 4 times with DMEM with 10% serum (Invitrogen), 4 times with PBS and detached using Trypsin (Biological Industries). The transfected cells were collected in Eppendorf tubes, resuspended in DMEM with 10% serum, and counted. Equal amounts of H2B-RFP and H2B-eGFP cells were mixed and seeded on glass-bottom plates (12-well black, glass-bottom #1.5H; Cellvis) and incubated at 37 °C and 5% CO_2_. 18 h after mixing, 20 µM 5-fluoro-2′-deoxyuridine (FdUrd) was added to the plates to arrest the cell cycle and 24 h later, the cells were fixed with 4% PFA in PBS and processed for immunofluorescence. To assay mixed cells and detect the transfected proteins (Fsx1-V5 or AtHAP2-V5), we stained cells with anti-V5 mAb (Life Science). The secondary antibody was Alexa Fluor 488 goat anti-mouse, with 1 µg/ml DAPI^22^. Micrographs were obtained using wide-field illumination using an ELYRA system S.1 microscope (Plan-Apochromat ×20 NA 0.8; Zeiss) and recorded with a iXon+ EMCCD camera (Andor). The GFP + RFP mixing index was calculated as the number of Red and Green nuclei in mixed cells out of the total number of nuclei of fluorescent cells in contact (Fig. 4b).

### Cell fusion assay by content mixing with nuclear and cytoplasmic markers

For the unilateral setup, BHK-21 cells were transfected (as explained above) with 1 µg pCI::GFPnes; pCI::Fsx1-V5::GFPnes; 0.25 µg pCI::EFF-1-V5::GFPnes; 1 µg pCI::VSV-G::GFPnes in respective 35 mm plates. The cells were incubated, washed, and mixed with pCI::H2B-RFP (empty vector) transfected cells (Fig. 4e). For evaluating the mutants, BHK-21 cells were transfected with 1 µg pCI::Fsx1-V5::GFPnes or pCI::Fsx1-V5::H2B-RFP or the plasmids encoding for each mutant: ΔFL → AG_4_A, ΔDIV → EFF-1 stem, ΔTMs → EFF-1 TM, Fsx1ΔTMs → GPI, or AtHAP2ΔTM → GPI (Fig. 5a). Empty pCI::GFPnes or pCI::H2B-RFP were used as negative controls. 4 h after transfection, the cells were washed, counted, mixed, and incubated as previously described. In all cases, 18 h after mixing, 20 µM FdUrd was added to the plates, and 24 h later, the cells were fixed with 4% paraformaldehyde diluted in PBS. Nuclei were stained with 1 µg/ml DAPI. Images were obtained using wide-field illumination with an ELYRA system S.1 microscope as described above.

The GFP + RFP mixing index was calculated as the number of nuclei in mixed cells, green cytoplasm (GFPnes) with red (H2B-RFP) and blue (DAPI) nuclei out of the total number of nuclei in fluorescent cells in contact (Figs. 4f, 5b). The multinucleation indexes were defined as the ratio between the number of nuclei in multinucleated cells (*N*_m_) and the total number of nuclei in multinucleated cells and expressing cells that were in contact (*N*_c_) but did not fuse, using the following equation: % multinucleation = *N*_m_/(*N*_c_ + *N*_m_) × 100. The percentage of multinucleation was calculated for GFPnes cells with RFP and DAPI nuclei. For the unilateral assay, multinucleation was determined as the ratio between the number of nuclei in multinucleated green cells and the total number of nuclei in green multinucleated cells and GFPnes expressing cells that were in contact but did not fuse (Fig. 4f).

### Live imaging of fusing cells

BHK cells were plated on 15 mm glass bottom plates (Wuxi NEST Biotechnology Co., Ltd.) and transfected with 1 µg pCI::Fsx1-V5::H2B-GFP together with 0.5 µg myristoylated-mCherry (myr-palm-mCherry; kindly provided by Valentin Dunsing and Salvatore Chiantia^123^). 18 h after transfection, the cells were incubated with 2 μg/ml Hoechst dye for 10 min at 37 °C and washed once with fresh medium. Time-lapse microscopy to identify fusing cells was performed using a spinning disc confocal microscope (CSU-X; Yokogawa Electric Corporation) with an Eclipse Ti and a Plan-Apochromat ×20 (NA, 0.75; Nikon) objective. Images in differential interference contrast and red channels were recorded every 4 min in different positions of the plate using high gain and minimum laser exposure. Time lapse images were captured with an iXon 3 EMCCD camera (Andor Technology). After 5 h, confocal z-series, including detection of the DAPI channel, were obtained to confirm the formation of multinucleated cells. Image analyses were performed in MetaMorph (Molecular Devices) and ImageJ^124^ (National Institutes of Health).

### Surface biotinylation

Proteins localizing on the surface were detected as previously described^22^. Briefly, BHK cells were transfected with 1 µg pCAGGS, pCAGGS::EFF-1-V5, pCAGGS::Fsx1-V5, pCAGGS::ΔFL → AG_4_A-V5, pCAGGS::ΔDIV → EFF-1 stem-V5 or pCAGGS::Fsx1ΔTMs → EFF-1 TM-V5. 24 h later, cells were washed twice with ice-cold PBS^2+^ (with Ca^2+^ and Mg^2+^) and incubated with 0.5 mg/ml EZ-Link Sulfo NHS-Biotin (Thermo Fisher Scientific) for 30 min on ice. The cells were washed four times with ice-cold PBS^2+^, once with DMEM with 10% FBS (to quench residual biotin), followed by two more washes with PBS^2+^. To each plate 300 µl of lysis buffer supplemented with 10 mM iodoacetamide were added and the cells detached using a scrapper. The insoluble debris was separated by centrifugation (10 min at 21,000 × *g*), and the lysate was mixed with NeutrAvidin Agarose Resin (Thermo Fisher Scientific) and 0.3% SDS. After an incubation of 12 h at 4 °C the resin was separated by centrifugation (2 min at 21,000 × *g*), washed three times with lysis buffer and then mixed with SDS–PAGE loading solution with freshly added 5% β-mercaptoethanol and incubated 5 min at 100 °C. After pelleting by centrifugation, the samples were separated by SDS–PAGE gel and analyzed by Western blotting as described above using anti-V5 mouse monoclonal antibody. Loading was controlled using anti-actin C4 monoclonal (1:2000; MP Biomedicals).

### Integrated mobile element (IME) identification by k-mer spectra analysis and comparative genomics

Comparison between close species with presence (*fsx1*+) or absence (*fsx1−*) of archaeal fusexins to detect insertion sites was done performing sequence similarity searches in complete genomes from the closest relatives available in the PATRIC database^125^ (Fig. 7a and Supplementary Table 4). Coordinates of *fsx1*-containing IMEs present in PCGs are annotated in Supplementary Table 4.

Among different methodologies that rely on DNA composition to identify horizontally transferred genomic regions^126^, k-mer spectrum analysis is a standard tool for this purpose^127,128^. Normalized k-mer spectra for DNA sequences of arbitrary length were generated by counting occurrences of all k-mers and normalizing by the total amount of words counted. k-mer sizes from 3 to 8 bp were tested with no effect on results. A length of 4 bp was selected. To detect possible horizontally transferred regions, an average spectrum for each genome was calculated. A spectrum was calculated for a sliding window of 1 kb using 500 bp steps and subtracted from the genomic average at each window position (Fig. 7b). The absolute value of the difference between the genomic average and window spectra is represented over the entire genome. Gaussian mixture models using two distributions were fitted^129^ to the k-mer content of all windows, to classify these as belonging to either the core genome or transferred elements. This deviation in k-mer spectra has been explored in the context of the archaeal mobilome and contains information on the ecological niche and evolutionary history of DNA sequences^130^.

### IME gene content and homology analyses

We followed the pipeline depicted in Supplementary Fig. 8. Briefly, PCGs’ IMEs were determined by a combination of k-mer spectra and genomic alignments (see Supplementary Table 4). We initially inspected *fsx1*-containing scaffolds and kept only sequences that were 20 kb or longer for downstream analyses. We generated an enriched annotation for each IME. Then, we obtained an initial set of groups of homologous sequences, and each of these groups was enriched by means of HMM searches. Subsequently, the enriched homology groups showing similarity between them, as judged by HMM–HMM comparisons, were collapsed into unique groups.

In detail, first, we re-annotated the identified mobile elements combining the corresponding segment of the PATRIC^125^ GFF annotation file with in-house ORF predictions (minimum ORF length of 30 nucleotides, option by default). ORF inference was done by means of getorf of the EMBOSS package^131^, specifying genetic code by Table 11 (Bacteria and Archaea) and other parameters running by default. The similarity of inferred ORFs and annotated features in these mobile elements (i.e., features in their GFF annotation file) was established by means of BLASTP reciprocal searches^74^. We kept all the predicted ORFs and homologs that were at least annotated in one genome, in this way we tried to recover missanotated conserved ORFs.

Initial sets of homologs were generated with get_homologs^132^. Sequence identity and query coverage thresholds were set to 35% and 70%, respectively. In-paralogues were not allowed within these groups (option ‘-e’), and remaining parameters were run by default.

HMM profiles were constructed for each homolog group. To this aim, homologous sequences were retrieved for members of each group from the UniRef50 database^133^ with jackhmmer from the HMMER package^73^ running with one iteration (‘-N 1’ parameter). MSAs were then generated for each group and its relevant hits with MAFFT^134^ running under ‘-auto’ parameter, and HMMs were created with HMMER hmmbuild. Homolog groups were enriched by means of HMM searches with HMMER hmmsearch, using each HMM as a query against a database comprising all predicted ORFs described above. Hits showing an e-value < 1e−10 and covering at least 50% of the HMM were added to the groups.

Enriched homology groups showing homology were collapsed. For this purpose, HMM-vs.-HMM comparisons were performed with HHalign from the HHsuite^135^. A graph was created with the networkx Python library (https://networkx.org), each node being an enriched group of homologs. An edge was established between nodes if their HMM-HMM alignment was significant (i.e., e-value < 1e−10, HMM coverage of longest HMM > = 50%). Groups of interconnected nodes were established with the ‘connected_components()’ routine, creating a collapsed homology group in each case.

Finally, we assessed the gene content similarity between mobile elements using a Jaccard Index based on the homology groups defined above. Usual Jaccard index of two sets is defined as (# of the intersection)/(# of the union). In this case:

$$J\,({{\rm {MEA},{MEB}}})=\frac{{N{ {{{{{\rm{homology}}}}}}\,{{{{{\rm{groups}}}}}}\,{{{{{\rm{shared}}}}}}\,{{{{{\rm{between}}}}}}\,{{{{{\rm{ME}}}}}}}}\,{\rm {A}}{\&}{{{{{\rm{ME}}}}}}\,{{{{{\rm{B}}}}}}}{N\,{{{{{{{\rm{homol}}}}}}.{{{{{\rm{groups}}}}}}\,{{{{{\rm{MEA}}}}}}}}+N\,{{{{{{{\rm{homol}}}}}}.{{{{{\rm{groups}}}}}}\,{{{{{\rm{MEB}}}}}}}}-{N{{{{{{\rm{homol}}}}}}.{{{{{\rm{groups}}}}}}\,{{{{{\rm{shared}}}}}}\,{{{{{\rm{between}}}}}}\,{{{{{\rm{ME}}}}}}}}\,{\rm {A}}{\&}{{{{{{\rm{ME}}}}}}\,{{{{{\rm{B}}}}}}}}$$

We performed a hierarchical clustering of the MEs based on a distance matrix obtained from the pairwise Jaccard Indexes (distance(A,B) = 1−*J*_A,B_). This was done in Python with seaborn^136^, employing the clustermap function. A subset of 11 mobile elements (red cluster in Supplementary Fig. 9), which included ME from PCGs and JGI12330J12834-1000008 (Supplementary Data 1, 3 and 4), was selected for synteny conservation analysis. Plots depicting synteny in gene content between homolog groups were generated employing the MCscan tool^137^.

HMMER and Pfam^138^ were used on default parameters to assign domains and their associated arCOG^139,140^ identifiers to ORFs (Supplementary Data 2).

These analyses, including collapsed clusters, can be found in Zenodo^63^.

### Sequence and structure phylogenies

Maximum-likelihood phylogenetic trees were generated with sequences aligned with MAFFT (L-INS-i option) as input for IQ-TREE2^141^ and selecting the best evolutionary model with ModelFinder^142^. Homology trimeric models of archaeal homologs of Fsx1_E_ were built with MODELLER using our crystal structure as template.

Protein folds preserve deeper evolutionary signals than sequences^143–145^. Fsx1 models and crystal structures of Fsx1_E_ and eukaryotic and viral fusexins were all-vs.-all compared with FATCAT^146^ to establish their structural distances between them. The following experimental crystal structures from other works were used: Flavivirus E: West Nile virus (2I69)^147^; Dengue virus serotype 1 (4GSX)^148^; Alphavirus E1: Semliki Forest virus (1RER)^149^; Chikungunya virus (3N43)^150^; *C. elegans* EFF-1 (4OJC)^16^; Bunyavirus Gc Rift Valley fever virus (6EGU)^151^; eukaryotic HAP2/GCS1 from *A. thaliana* (5OW3)^25^ and *C. reinhardtii* (6E18)^65^. The PDB files produced by flexible alignment with FATCAT were compared with TMalign^152^ to build a TM_score_^153^ distance matrix (distance = 1−TM_score_). This distance matrix was the basis to compute a minimum evolution tree with FastME^154^ on default parameters (Supplementary Fig. 10b).

### Reporting summary

Further information on research design is available in the Nature Research Reporting Summary linked to this article.

## Supplementary information

Supplementary Information

Description of Additional Supplementary Files

Reporting Summary

Peer Review File

Supplementary data 1

Supplementary Data 2

Supplementary Data 3

Supplementary Data 4

Supplementary Data 5

Supplementary Movie 1

Supplementary Movie 2

## Data Availability

Crystallographic structure factors and atomic coordinates have been deposited in the Protein Data Bank under accession code 7P4L. Source Data for this paperʼs structure-function analyses (Figs. 4 and 5, and Supplementary Fig. 1) are provided in the Source Data file; sequences of synthesized *fsx1* genes are in Supplementary Data 4. Source data are provided with this paper.

## Source data

Source Data

## Code availability

All relevant codes, notebooks and datasets necessary for: HHblits and HMMER searches and comparisons (Fig. 1a, Supplementary Fig. 1a); genomic comparisons and K-mer spectra analyses (Fig. 7); bioinformatic workflow for IMEs, IMEs clustering, content and synteny analyses (Supplementary Figs. 8 and 9, Fig. 8 and Supplementary Data 2 and 3); and structure- and sequence-based comparisons (Supplementary Fig. 10 and Supplementary Data 5), are provided on GitHub (https://github.com/DessimozLab/Archaeal-Fusexins) and Zenodo (10.5281/zenodo.6677729)^63^.
