# Supplementary material for: Discovery of archaeal fusexins homologous to eukaryotic HAP2/GCS1 gamete fusion proteins
Source: Nat Commun. 2022 Jul 6;13:3880. doi: 10.1038/s41467-022-31564-1 (PMC9259645; doi:10.1038/s41467-022-31564-1)
Supplement: Supplementary file 1 — Supplementary Information [file 41467_2022_31564_MOESM1_ESM.pdf]

Supplementary Information for

Discovery of archaeal fusexins homologous to eukaryotic HAP2/GCS1 gamete fusion proteins

David Moi[1,2,3]†, Shunsuke Nishio[4,]†, Xiaohui Li[5,]†, Clari Valansi[5], Mauricio Langleib[6,7], Nicolas G. Brukman[5], Kateryna Flyak[5], Christophe Dessimoz[2,3,8,9], Daniele de Sanctis[10], Kathryn Tunyasuvunakool[11], John Jumper[11], Martin Graña[7,]*, Héctor Romero[6,12,]*, Pablo S. Aguilar[1,13,]*, Luca Jovine[4,]*, Benjamin Podbilewicz[5,]*

[1]Instituto de Fisiología, Biología Molecular y Neurociencias (IFIBYNE-CONICET), Buenos Aires, Argentina.

[2]Department of Computational Biology, University of Lausanne, Lausanne, Switzerland.

[3]Swiss Institute of Bioinformatics, Lausanne, Switzerland.

[4]Department of Biosciences and Nutrition, Karolinska Institutet, Huddinge, Sweden.

[5]Department of Biology, Technion- Israel Institute of Technology, Haifa, Israel.

[6]Unidad de Genómica Evolutiva, Facultad de Ciencias, Universidad de la República, Uruguay.

[7]Unidad de Bioinformática, Institut Pasteur de Montevideo, Uruguay.

[8]Centre for Life's Origins and Evolution, Dept. of Genetics, Evolution and Environment, University College London, United Kingdom.

[9]Department of Computer Science, University College London, United Kingdom.

[10]The European Synchrotron, Grenoble, France

[11]DeepMind, London, UK

[12]Centro Universitario Regional Este - CURE, Centro Interdisciplinario de Ciencia de Datos y Aprendizaje Automático - CICADA, Universidad de la República, Uruguay.

[13]Instituto de Investigaciones Biotecnológicas Universidad Nacional de San Martín (IIB-CONICET), San Martín, Buenos Aires, Argentina.

***Corresponding authors. Email:** mgrana@pasteur.edu.uy, eletor@fcien.edu.uy, paguilar@iib.unsam.edu.ar, luca.jovine@ki.se, podbilew@technion.ac.il

†These authors contributed equally to this work.

This PDF file includes:

Supplementary Figure 1……page 3
Supplementary Figure 2……page 6
Supplementary Figure 3……page 8
Supplementary Figure 4……page 9
Supplementary Figure 5……page 10
Supplementary Figure 6……page 12
Supplementary Figure 7……page 15
Supplementary Figure 8……page 19
Supplementary Figure 9……page 21
Supplementary Figure 10……page 22
Supplementary Figure 11……page 23
Supplementary Figure 12……page 24

Supplementary Table 1……page 25
Supplementary Table 2……page 26
Supplementary Table 3……page 27
Supplementary Table 4……page 28
Supplementary Table 5……page 29
Supplementary Table 6……page 30
Supplementary Table 7……page 32

Supplementary References……page 34

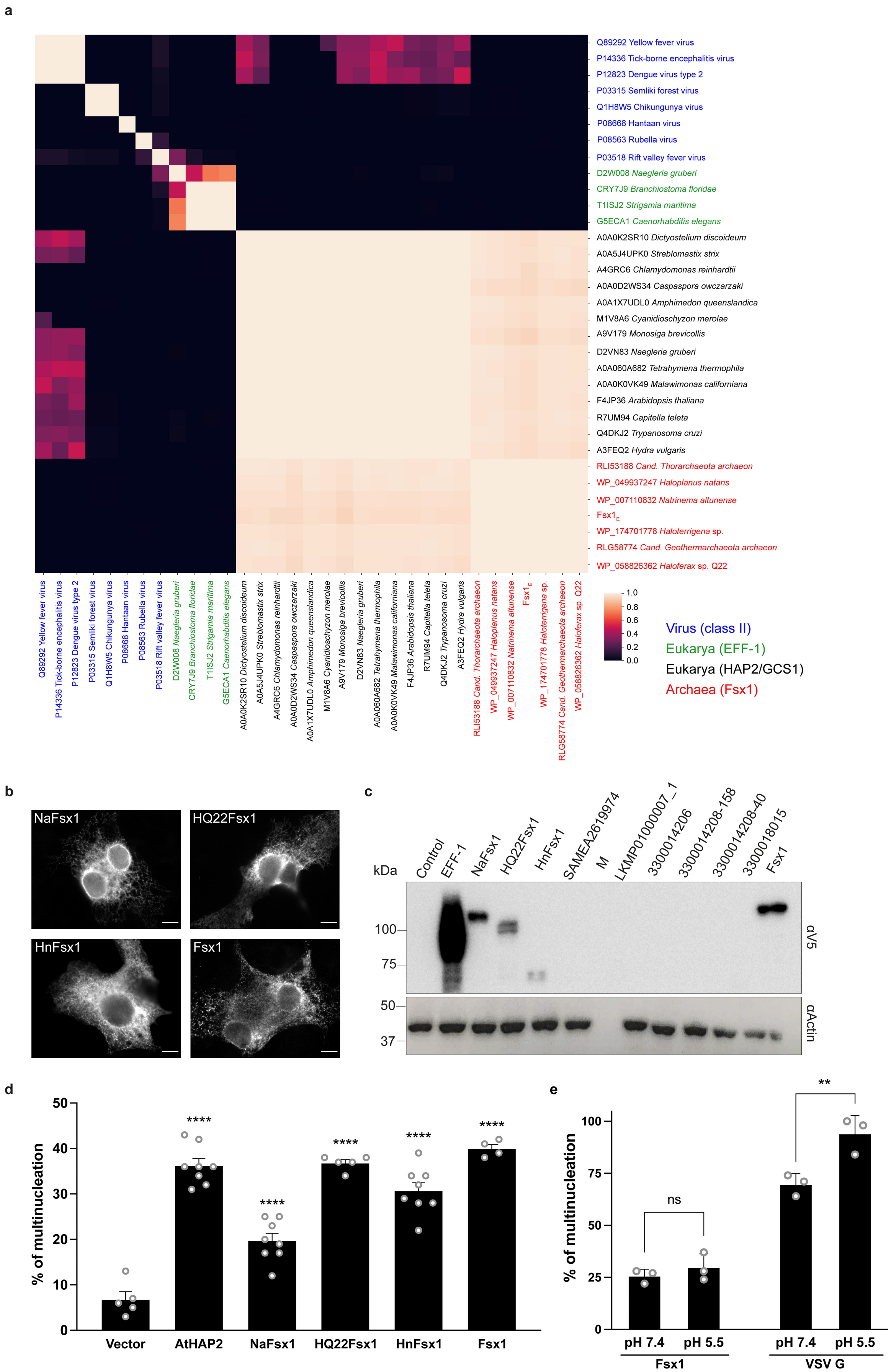
a
Q89292 Yellow fever virus
P14336 Tick-borne encephalitis virus
P12823 Dengue virus type 2
P03315 Semliki forest virus
Q1H8W5 Chikungunya virus
P08668 Hantaan virus
P08563 Rubella virus
P03518 Rift valley fever virus
D2W008 Naegleria gruberi
CRY7J9 Branchiostoma floridae
T1ISJ2 Strigamia maritima
G5ECA1 Caenorhabditis elegans
A0A0K2SR10 Dictyostelium discoideum
A0A5J4UPK0 Streblomastix strix
A4GRC6 Chlamydomonas reinhardtii
A0A0D2WS34 Caspaspora owczarzaki
A0A1X7UDL0 Amphimedon queenslandica
M1V8A6 Cyanidioschyzon merolae
A9V179 Monosiga brevicollis
D2VN83 Naegleria gruberi
A0A060A682 Tetrahymena thermophila
A0A0K0VK49 Malawimonas californiana
F4JP36 Arabidopsis thaliana
R7UM94 Capitella teleta
Q4DKJ2 Trypanosoma cruzi
A3FEQ2 Hydra vulgaris
RLI53188 Cand. Thorarchaeota archaeon
WP_049937247 Haloplanus natans
WP_007110832 Natrinema altunense
Fsx1E
WP_174701778 Haloterrigena sp.
RLG58774 Cand. Geothermarchaeota archaeon
WP_058826362 Haloferax sp. Q22
1.0
0.8
0.6
0.4
0.2
0.0
Virus (class II)
Eukarya (EFF-1)
Eukarya (HAP2/GCS1)
Archaea (Fsx1)
b
NaFsx1
HQ22Fsx1
HnFsx1
Fsx1
c
kDa
Control
EFF-1
NaFsx1
HQ22Fsx1
HnFsx1
SAMEA2619974
M
LKMP01000007_1
3300014206
3300014208-158
3300014208-40
3300018015
Fsx1
100
75
50
37
αV5
αActin
d
% of multinucleation
50
40
30
20
10
0
****
Vector
AtHAP2
NaFsx1
HQ22Fsx1
HnFsx1
Fsx1
e
% of multinucleation
100
75
50
25
0
ns
**
pH 7.4
pH 5.5
Fsx1
VSV G

Supplementary Fig. 1. Sequence similarities between fusexins and ectopic expression of archaeal fusexins in mammalian cells.

a Fsx1s are members of the fusexin superfamily. HMM homology probabilities of fusexins and archaeal candidates ectodomains evidence sequence similarity between archaeal candidates and sexual (HAP2/GCS1) fusexins. HMMs were constructed for each ectodomain sequence (UniProt and NCBI identifiers shown). HAP2/GCS1 and EFF-1 sequences were chosen from representative species of the major eukaryotic lineages where these fusexins are present. Flavi-, alpha-, rubi- and bunyaviruses encompass all currently known viral fusexins. All vs all probabilities of homology as determined by HHblits were clustered along rows and columns using UPGMA with Hamming distance. Several sequences selected for this analysis have corresponding crystal structures: Yellow fever virus (UniProt Q89292], PDB 6IW5[1]), Chikungunya virus (UniProt Q1H8W5, PDB 3N43[2]), Dengue virus (UniProt P12823, PDB 1OAN[3]), Semliki forest virus (UniProt P03315, PDB 1RER[4]), Tick-borne encephalitis virus (UniProt P14336, PDB 1SVB[5]), *Arabidopsis thaliana* (UniProt F4JP36, PDB 5OW3[6]), Rubella virus (UniProt P08563, PDB 4ADG[7]), *Chlamydomonas reinhardtii* (UniProt A4GRC6, PDB 5MF1[8]), Hantavirus (UniProt P08668, PDB 5LK1[9]) and Rift valley fever virus (UniProt P03518, PDB 4HJC[10]). Although all of the sequences used as input belong to the fusexin structural superfamily, HMM vs HMM comparisons can only detect homology within subsets of the superfamily.

b-d Ectopic expression of archaeal fusexins in BHK cells. **b-c** Ten archaeal genes were synthesized (Supplementary Table 5) and independently expressed in BHK cells using an inducible promoter. **b** Immunofluorescence (n=2) and **c** Western blot showing ectopic expression detected with anti-V5 antibody (n=2). EFF-1 from *C. elegans* was used as a positive control. NaFsx1, *Natrinema altunense* Fsx1. HQ22Fsx1, *Haloferax* sp. Q22 Fsx1. HnFsx1, *Haloplanus natans* Fsx1. LKMP01000007_1 was obtained from Nanohaloarchaea B1-Br10_U2g21 LB-BRINE-C121. Fsx1 (the protein subsequently characterized), SAMEA2619974 and sequences starting with "330" were obtained from metagenomic databases (see Supplementary Table 5 for complete accession numbers). M, marker. Scale bars, 10 µm.

d Quantification of multinucleation in cells expressing archaeal fusexins. Cells were transfected with archaeal fusexins cloned into pCI::H2B-RFP/GFP vectors

separately. 48 h post-transfection, immunofluorescence was performed with anti-V5 antibody. Empty vector pCI::H2B-RFP or pCI::H2B-GFP co-transfected with myr-EGFP were the negative controls. AtHAP2 was used as a positive control. Multinucleation was determined as the ratio between the number of nuclei in multinucleated cells and the total number of nuclei in multinucleated cells and expressing cells that were in contact but did not fuse. The percentage of multinucleation is presented as individual data and means ± SEM of independent experiments ($n \geq 4$). Total number of nuclei counted in multinucleated cells and in cells in contact $n \geq 1,000$ for each experimental condition. Comparisons were made with one-way ANOVA followed by Dunett's test against the empty vector. **** $p < 0.0001$. **e** VSV G activity, but not Fsx1, is enhanced at low pH. Quantification of multinucleation in cells expressing Fsx1 or VSV G. Cells were transfected with pCI::H2B-RFP bearing the coding sequence for Fsx1 or VSV G. 48 h post-transfection, a 5-minute incubation at pH 5.5 buffer was performed to some cells. After 2 h, cells were fixed and immunofluorescence was performed with anti-V5 antibody or anti-G for Fsx1 and VSV G, respectively. Multinucleation was determined as the ratio between the number of nuclei in multinucleated cells and the total number of nuclei in multinucleated cells and expressing cells that were in contact but did not fuse. The percentage of multinucleation is presented as individual data and means ± SEM of three independent experiments. Total number of nuclei counted in multinucleated cells and in cells in contact $n \geq 1,000$ for each experimental condition. Comparisons were made with two-way ANOVA. ns, non-significant, ** $p < 0.01$. Source data are provided as a Source Data file.

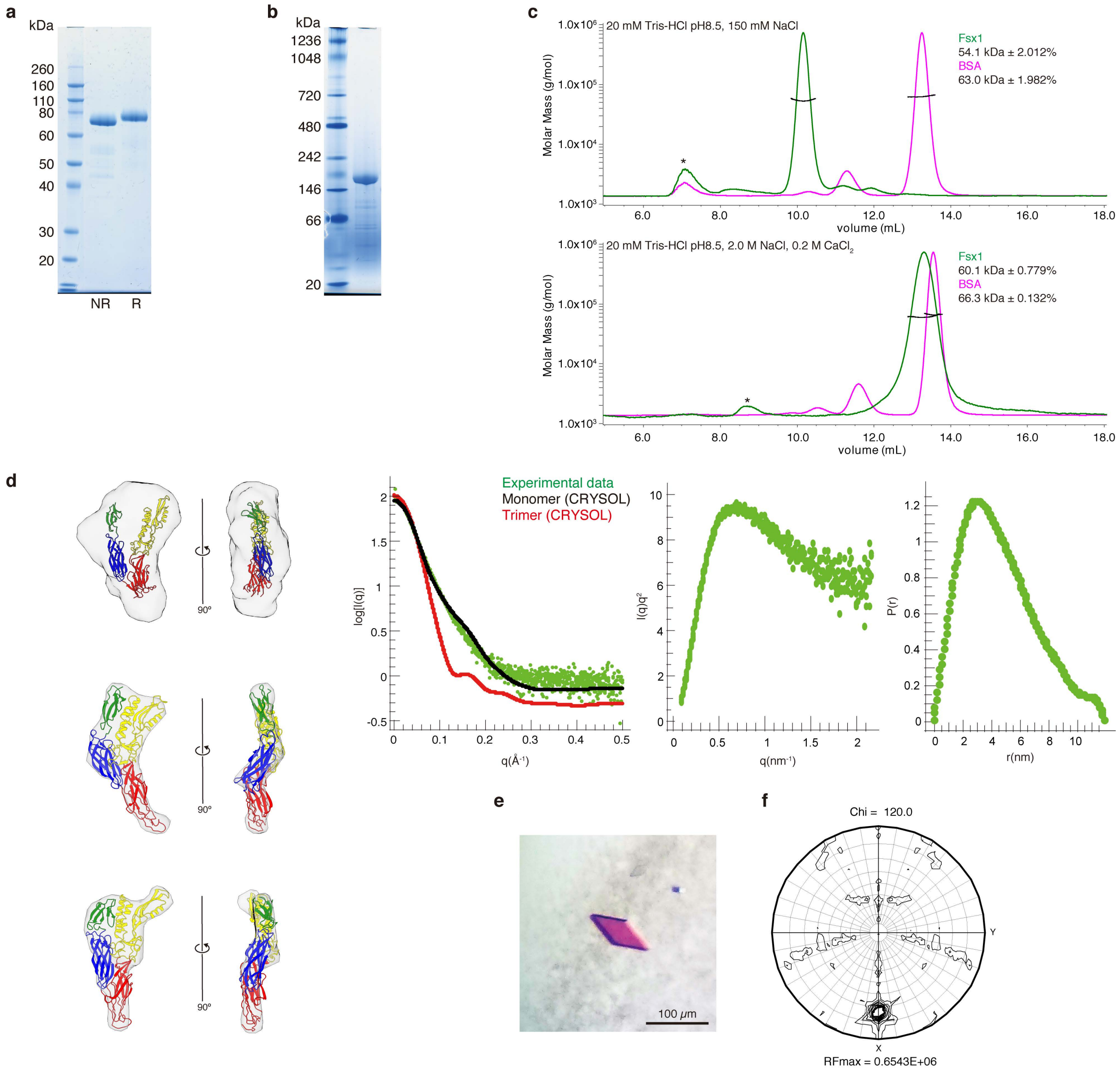

Supplementary Fig. 2 The Fsx1 ectodomain is a monomer in solution but crystallizes as a trimer.

a, b SDS-PAGE (a) and blue native PAGE (b) gels of purified Fsx1 ectodomain ($Fsx1_E$). NR; non-reducing conditions. R; reducing conditions (see Supplementary Fig. 11). n=2.

c Size exclusion chromatography-multiangle light scattering (SEC-MALS) shows that, although $Fsx1_E$ has a very different elution volume depending on the salt concentration, it is a monomer in solution in both normal and high salt conditions. BSA, whose elution volume does not change significantly at different salt concentrations, is used as a control. Asterisks indicate the high-molecular weight aggregate. n=2.

d SAXS analysis of $Fsx1_E$. Left panel, The SAXS envelope of $Fsx1_E$, obtained by averaging 20 *ab initio* shape reconstructions, is consistent with the crystallographic model of $Fsx1_E$ chain A (top). However, as also indicated by the corresponding Kratky plot (see below), its relatively broad profile suggests that there is some flexibility between the domains of Fsx1 in solution. Accordingly, improved agreements can be obtained by flexibly fitting the $Fsx1_E$ monomer model to envelopes generated by averaging the two most abundant clusters of SAXS models (middle and bottom). Center-left panel, Comparison of the experimental SAXS profile of $Fsx1_E$ (green dots) and theoretical scattering curves calculated from the refined coordinates of $Fsx1_E$ chain A (black dots) or the whole $Fsx1_E$ trimer (red dots). Center-right panel, the Kratky plot of $Fsx1_E$ suggests the presence of significant flexibility between the domains of the monomeric protein. Right panel, Pairwise interatomic distance distribution of $Fsx1_E$.

e Representative rhomboidal plate crystal of $Fsx1_E$.

f The Chi=120 section of the self-rotation function of $Fsx1_E$ (calculated using a 67.3-2.6 Å resolution range) shows a prominent peak with a height of 72% of the origin peak.

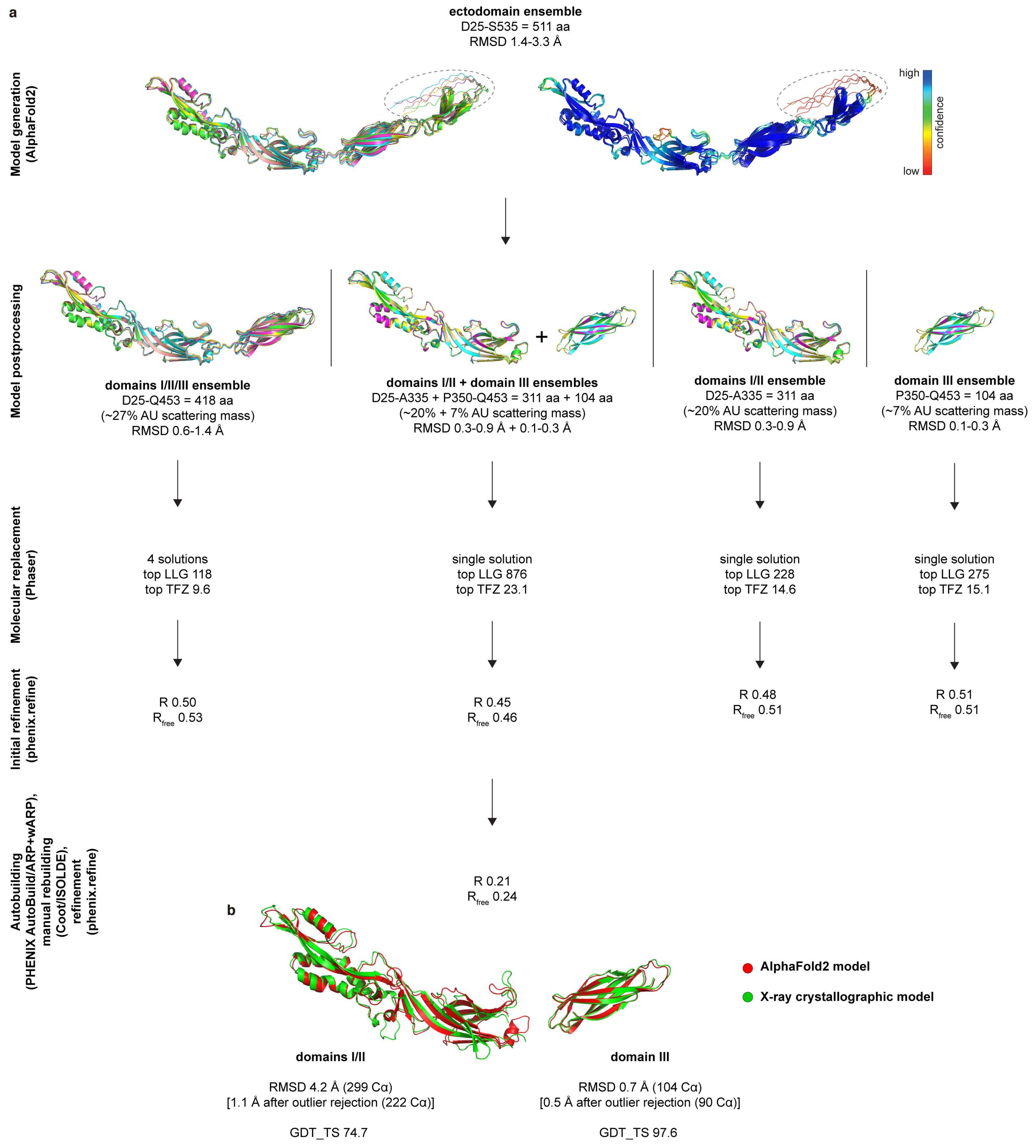

Supplementary Fig. 3 AlphaFold2-aided MR phasing of Fsx1$_E$.

a Flowchart of Fsx1$_E$ structure determination using different AlphaFold2 model fragments or a combination thereof. The top panel shows a superposition of the five initial predictions, colored by model (left) or by confidence (right); the dashed ovals indicate C-terminal residues D510-S535, which were predicted with low confidence by AlphaFold2. aa, amino acid; AU, asymmetric unit.

b Comparison of the top-ranked AlphaFold2 prediction and the refined experimental model of Fsx1 domains I/II and III.

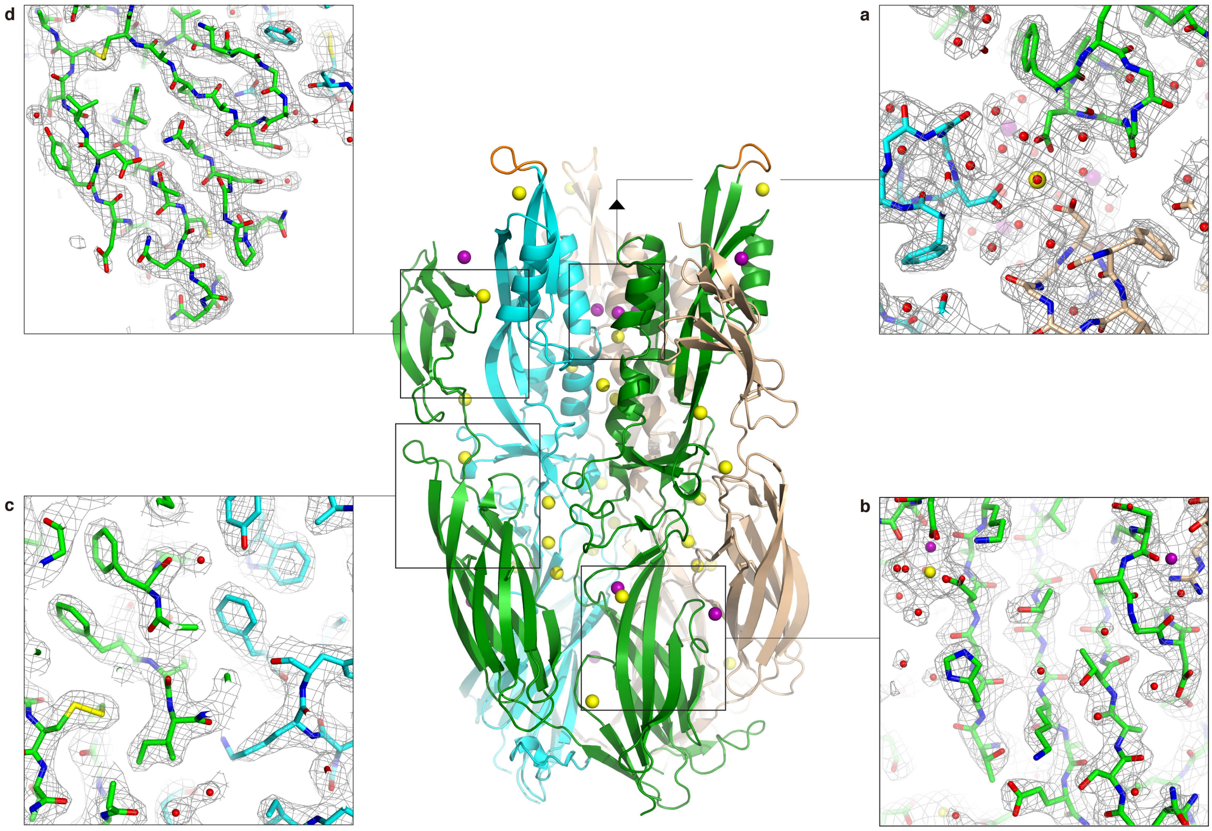

Supplementary Fig. 4 Details of the electron density map of Fsx1$_E$.

a View of the domain II helical bundle, looking down the molecular three-fold axis from the center of the structure towards the putative fusion loop end. Domain II α1 helix residues D260 and D263 coordinate a Ca^{2+} ion sitting on the NCS axis and three symmetrically positioned Na^+ ions, respectively. The refined *2mFo-DFc* electron density map, contoured at 1.0 σ, is shown as a gray mesh superimposed onto the protein model in stick representation. Fsx1 subunits and metal ions are coloured as in Fig. 3c.

b Section of the map centered around β-strands D_0, E_0 and F_0 of domain I.

c Closeup of the map region where domain III of one protein subunit interacts with domains I and II of another. Clear density for the $C_3$389-$C_4$432 disulfide is visible near the bottom left corner.

d Map of domain IV. The conserved $C_7$490-$C_8$506 disulfide is at the top left corner, whereas $C_6$477 of the $C_5$457-$C_6$477 disulfide is visible at the bottom and the domain II $C_1$125-$C_2$155 disulfide of the adjacent subunit can be seen on the top right corner.

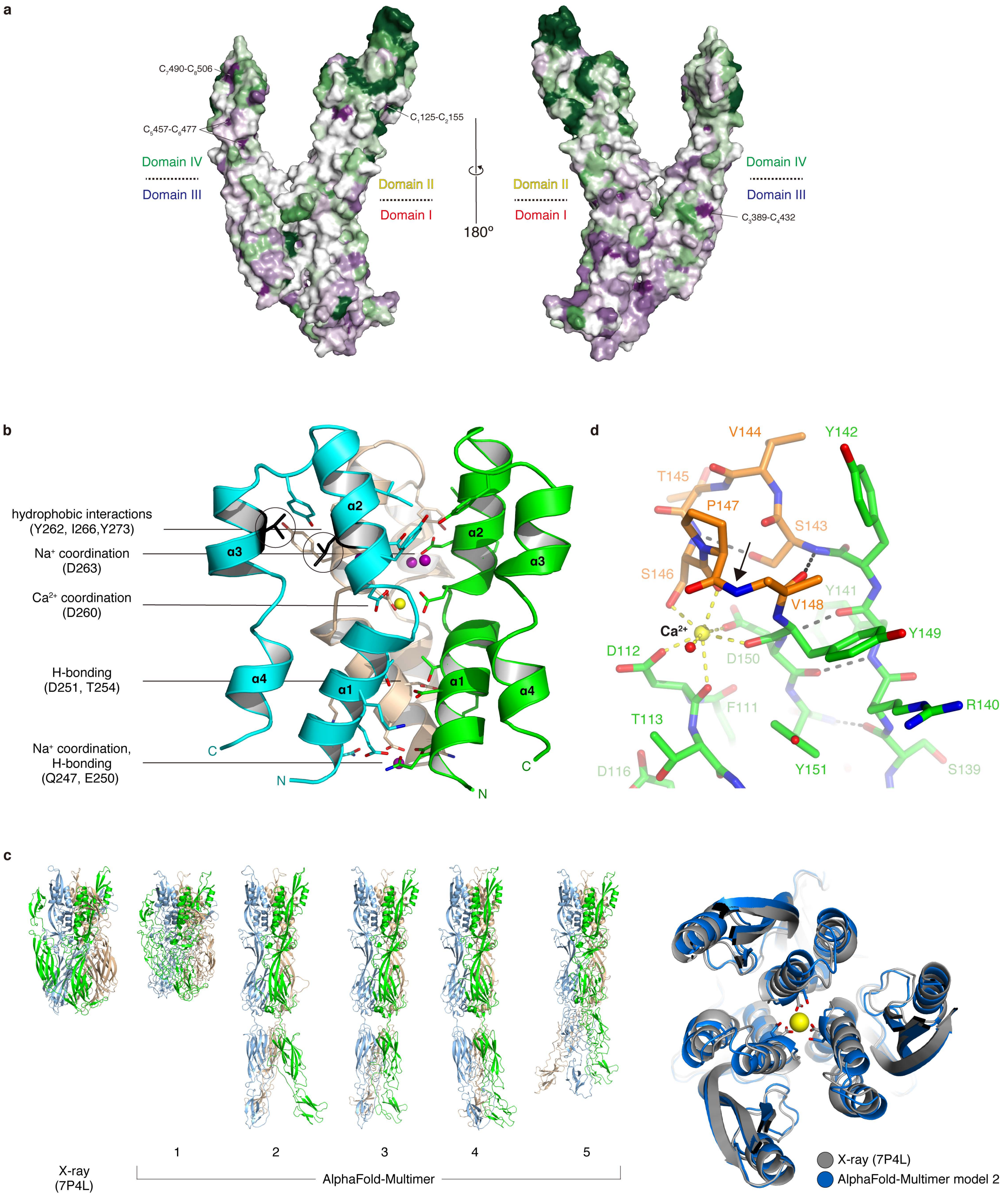

Supplementary Fig. 5 3D mapping of the evolutionary conservation of Fsx1 residues and key structural features of its domain II.

a Fsx1 domains I and III are more evolutionarily conserved than domains II and IV. Surface representation of the $Fsx1_E$ monomer, with residues coloured from green to violet by increasing conservation among archaeal homologs. Approximate domain

boundaries are marked, and the position of the four highly conserved disulfides of Fsx1 is indicated.

b Helices α1 and α2 of domain II form a six-helix bundle around the molecular three-fold axis. Subunits are shown in cartoon representation and coloured as in Fig. 3c, with residues mediating direct or ion-mediated interactions between chains depicted in stick representation (for clarity, water-mediated interactions are not shown). The side chains of residues α2 L264 and α3 V278 (black circles), which face each other in the $Fsx1_E$ structure, are both replaced by Cys in the sequences of *Halogeometricum borinquense* and *Halobonum* sp. (Supplementary Fig. 6), suggesting that an additional disulfide bond stabilizes the helical bundle of the Fsx1 homologs from these species.

c AlphaFold-Multimer prediction of the $Fsx1_E$ homotrimer (left panel) generates models that either approximate the overall post-fusion conformation of the crystal structure (albeit with significant interchain clashes; model 1) or adopt an extended conformation that resembles an intermediate state thought to exist before fusion[11] (models 2-5). In both cases, the models contain the experimentally observed six-helix bundle and, as shown for the Ca^{2+} coordinated by D260 (yellow sphere), in some instances even reproduce the orientation of side chains that bind ions in the crystal (right panel). Considering that these residues are poorly conserved in other homologs of Fsx1 (Supplementary Fig. 6) and taking into account that AlphaFold2 does not explicitly predict ions, this suggests that Ca^{2+} and Na^{+} stabilize the trimeric structure of Fsx1 rather than being required for its formation.

d Structure of the fusion loop of $Fsx1_E$. The domain II region encompassing the loop is shown in the same orientation as in Fig. 3f, with black and yellow dashes indicating protein hydrogen bonds and the coordination of the Ca^{2+} ion, respectively. Note how binding of the ion locally twists the protein main chain, with the peptide bond between P147 and V148 adopting a *cis* configuration (black arrow).

a

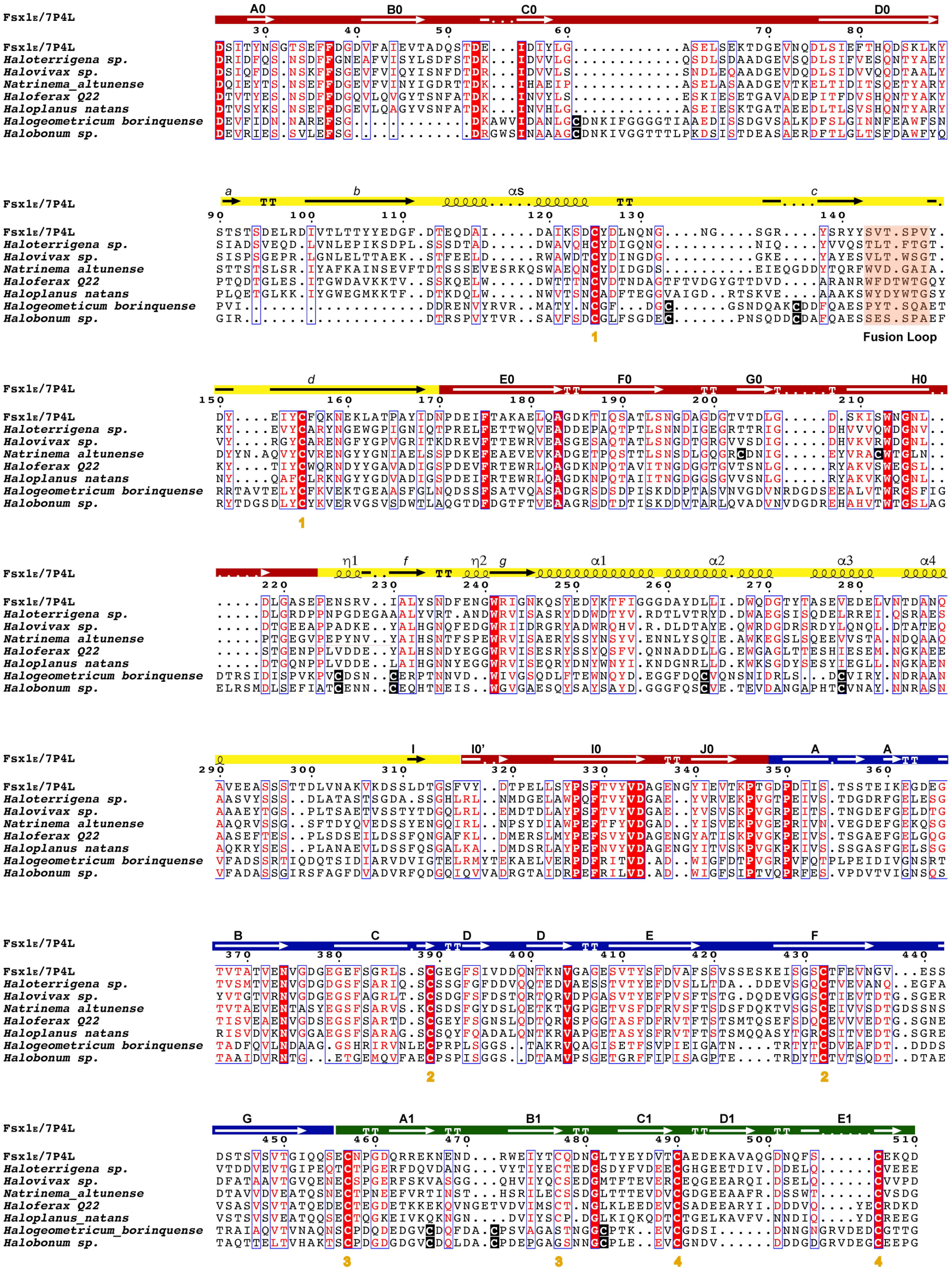
Fsx1E/7P4L
Haloterrigena sp.
Halovivax sp.
Natrinema altunense
Haloferax Q22
Haloplanus natans
Halogeometricum borinquense
Halobonum sp.
Fusion Loop

b

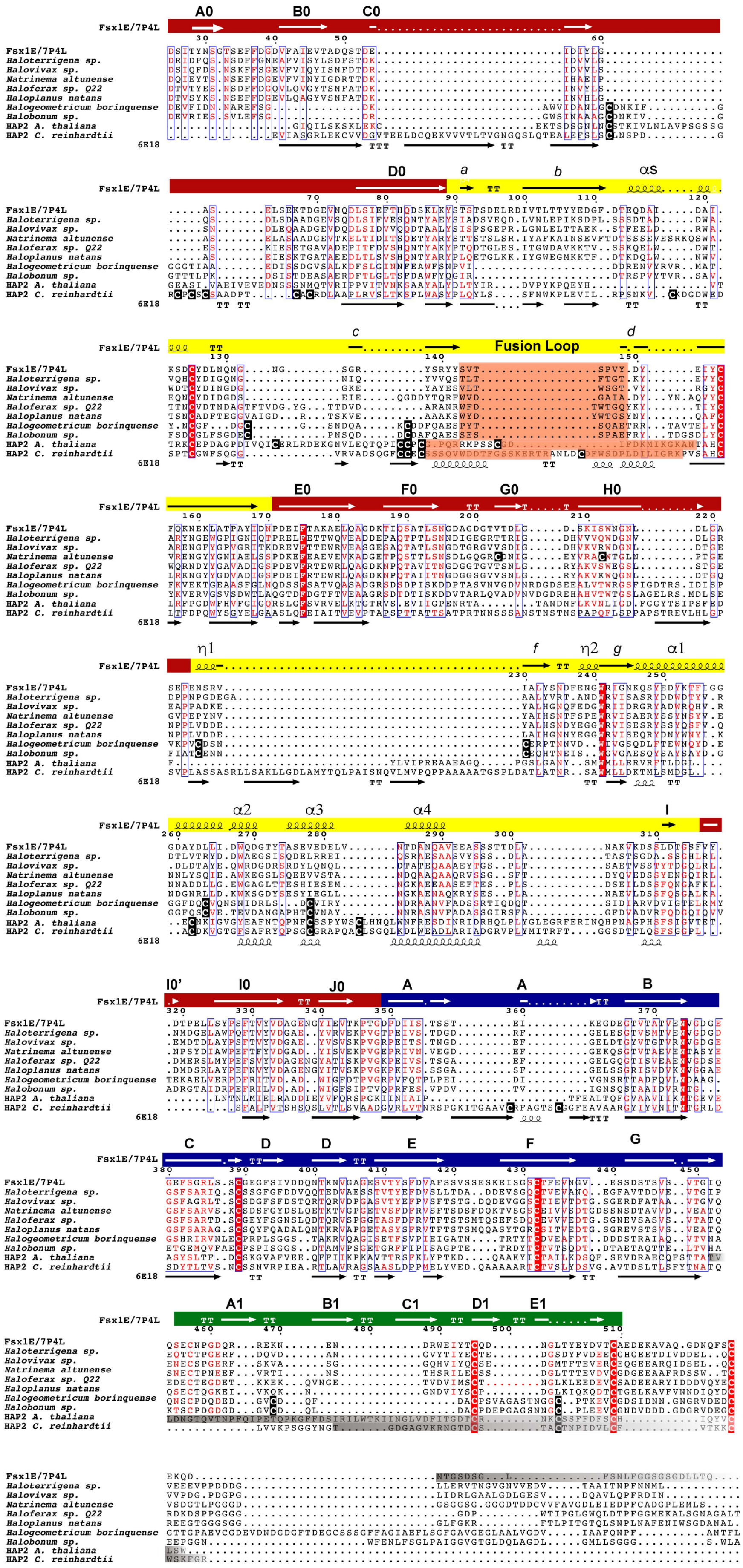

Fsx1E/7P4L
A0
B0
C0
D0
a
b
αS
c
Fusion Loop
d
E0
F0
G0
H0
η1
f
η2
g
α1
α2
α3
α4
I
I0'
I0
J0
A
B
C
D
E
F
G
A1
B1
C1
D1
E1
Haloterrigena sp.
Halovivax sp.
Natrinema altunense
Haloferax sp. Q22
Haloplanus natans
Halogeometricum borinquense
Halobonum sp.
HAP2 A. thaliana
HAP2 C. reinhardtii
6E18

Supplementary Fig. 6 Alignments of archaeal Fsx1s from cultivated species and with HAP2 fusexin ectodomains.

For reproducibility, no gap or block was altered from alignments. Identical column residues are depicted in bold white on a red background; conserved positions are boxed and labeled red. On top are displayed secondary structure elements and sequence numbering corresponding to $Fsx1_E$/7P4L.

a Archaeal sequences from pure culture genomes (PCGs), with N- and C-terminal regions cropped to match $Fsx1_E$ (PDB 7P4L). Secondary structure elements are shown within boxed domains, coloured and labeled following the previous nomenclature (domain I, red; domain II, yellow; domain III, blue). Disulfide bonds are indicated by orange numbers below the alignment. Additional, lineage-specific cysteines are black-boxed and depicted in bold white. The fusion (cd) loop is highlighted in light orange within the alignment; as in eukaryotic HAP2, it has poor sequence conservation (Supplementary Fig. 5a) but shows a high prevalence of hydrophobic residues (Fig. 6). Domain IV (green) has relatively poor sequence conservation within archaea (Supplementary Fig. 5a), yet preserves its disulfide bonds.

b Sequence alignment of HAP2s from *A. thaliana* and *C. reinhardtii* (PDB 6E18[12]) and archaeal fusexins from cultivated genomes. Of note are the inserts of HAP2 relative to Fsx1s, as well as the absence of domain IV, which seems confined to Archaea. The conserved fusion loop region is orange shaded; lineage-specific cysteines black boxed. C-terminal regions shaded with a gradient from gray to white indicate portions absent from $Fsx1_E$ (PDB 7P4L) and HAP2 (PDB 5OW3 and 6E18) crystal structures, either with no electron density or absent from the expression construct. HAP2s introduce gaps; for clarity, disulfide bonds shown in panel a are omitted and replaced by secondary structure elements from PDB 6E18.

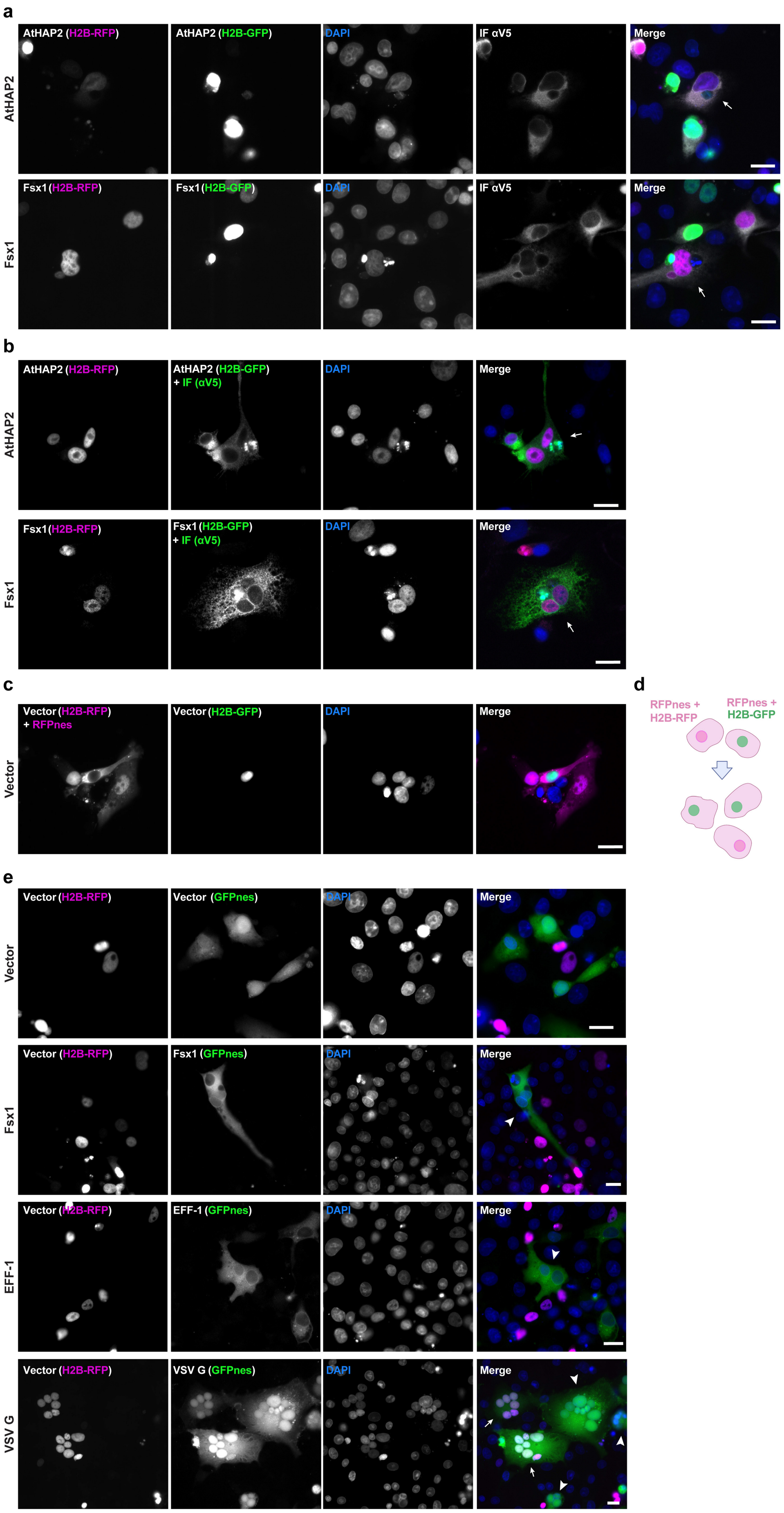
a
AtHAP2
AtHAP2 (H2B-RFP)
AtHAP2 (H2B-GFP)
DAPI
IF αV5
Merge
Fsx1
Fsx1 (H2B-RFP)
Fsx1 (H2B-GFP)
DAPI
IF αV5
Merge
b
AtHAP2
AtHAP2 (H2B-RFP)
AtHAP2 (H2B-GFP) + IF (αV5)
DAPI
Merge
Fsx1
Fsx1 (H2B-RFP)
Fsx1 (H2B-GFP) + IF (αV5)
DAPI
Merge
c
Vector
Vector (H2B-RFP) + RFPnes
Vector (H2B-GFP)
DAPI
Merge
d
RFPnes + H2B-RFP
RFPnes + H2B-GFP
e
Vector
Vector (H2B-RFP)
Vector (GFPnes)
DAPI
Merge
Fsx1
Vector (H2B-RFP)
Fsx1 (GFPnes)
DAPI
Merge
EFF-1
Vector (H2B-RFP)
EFF-1 (GFPnes)
DAPI
Merge
VSV G
Vector (H2B-RFP)
VSV G (GFPnes)
DAPI
Merge

f

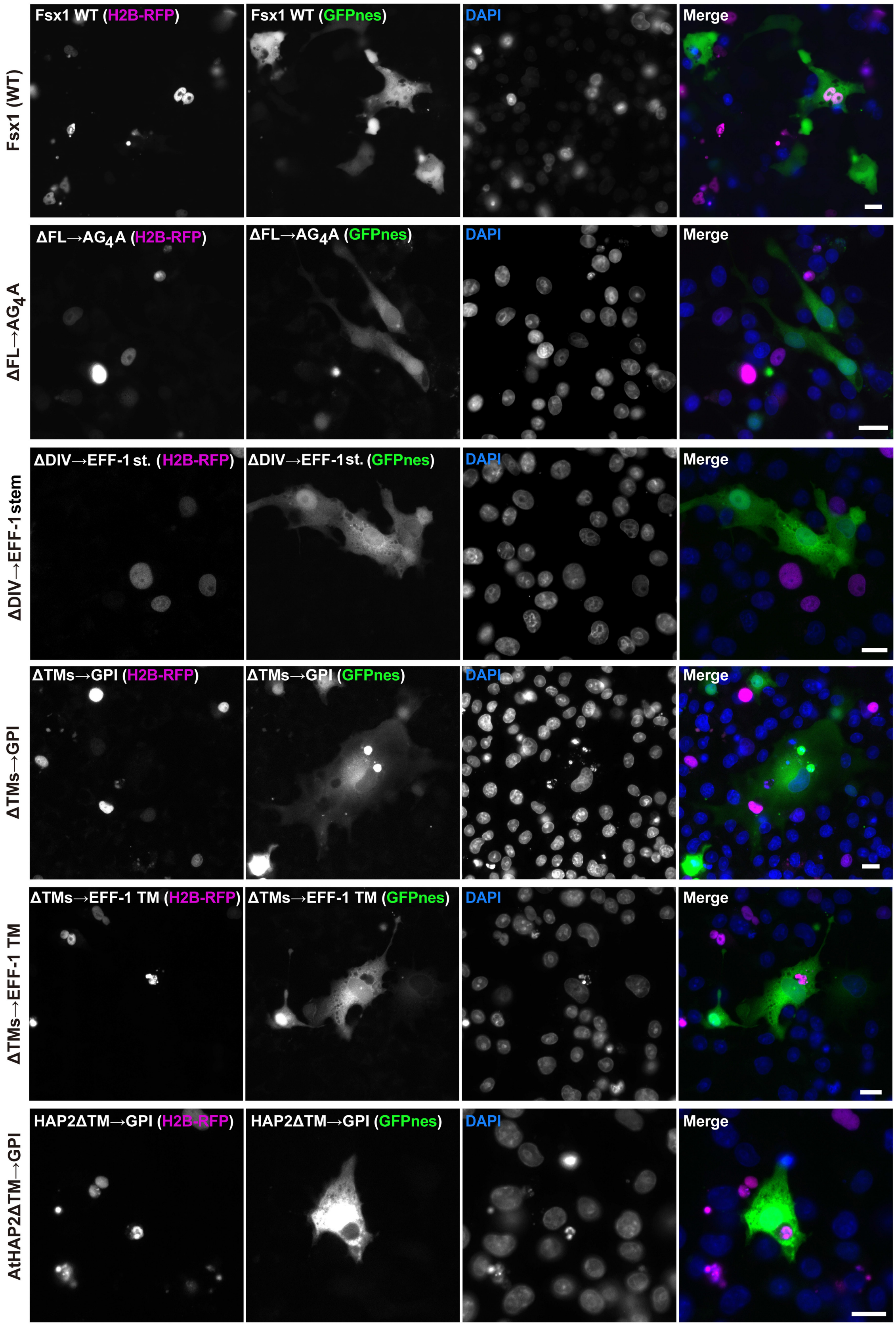

Fsx1 WT (H2B-RFP)
Fsx1 WT (GFPnes)
DAPI
Merge
Fsx1 (WT)
ΔFL→AG4A (H2B-RFP)
ΔFL→AG4A (GFPnes)
ΔFL→AG4A
ΔDIV→EFF-1 st. (H2B-RFP)
ΔDIV→EFF-1 st. (GFPnes)
ΔDIV→EFF-1 stem
ΔTMs→GPI (H2B-RFP)
ΔTMs→GPI (GFPnes)
ΔTMs→GPI
ΔTMs→EFF-1 TM (H2B-RFP)
ΔTMs→EFF-1 TM (GFPnes)
ΔTMs→EFF-1 TM
HAP2ΔTM→GPI (H2B-RFP)
HAP2ΔTM→GPI (GFPnes)
AtHAP2ΔTM→GPI

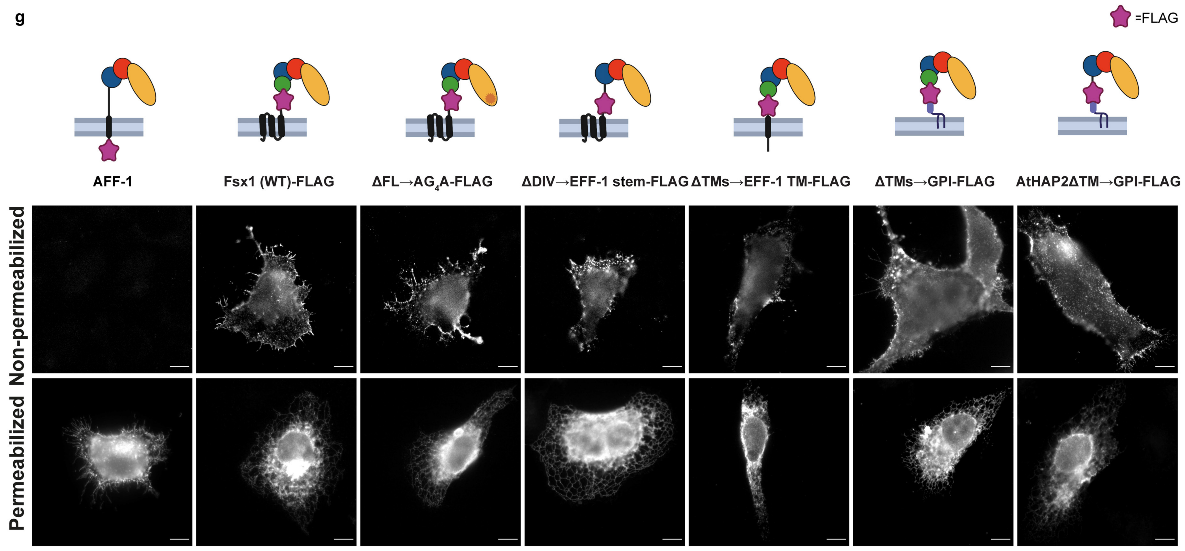

Supplementary Fig. 7 Fsx1 mediates bilateral cell-cell fusion and structure-function analysis.

a Images from Fig. 4a in each separate channel (red, green, DAPI and far red) and merge. Scale bars, 20 µm. n=4.

b Multinucleated cells containing green nuclei (H2B-GFP) and magenta nuclei (H2B-RFP) (arrows). Immunofluorescence against the V5 tag was performed in green to facilitate counting. n=4.

c, d in the negative control, cell-cell fusion was measured by content-mixing, indicated by the appearance of multinucleated cells containing green nuclei (H2B-GFP) and magenta nuclei (H2B-RFP). To reveal the cytoplasm of the transfected cells, a plasmid encoding for cytoplasmic RFP (RFPnes) was co-transfected. n=3. **c** Representative images of mononucleated cells with a green or red nucleus. DAPI staining is shown in blue. **d** Cartoon showing the experimental design for negative control.

e Images from Fig. 4d in each separate channel (red, green and DAPI) and merge. Multinucleated GFPnes only (arrowheads) or mixed cells (arrows). Scale bars, 20 µm. n=3.

f Images from Fig. 5c in each separate channel: red (RFP); green (GFP) and blue (DAPI) and the merged images. Scale bars, 20 µm. n>3 as specified in Fig. 5b for each condition.

g Surface expression of Fsx1, mutants and eukaryotic fusexins. For colors and abbreviations see legend of Fig. 2. BHK cells were transfected with FLAG-tagged

Fsx1 (WT) and the indicated mutants; the FLAG tag was inserted before the membrane anchor. Non-permeabilized staining using anti-FLAG antibody showed surface expression of Fsx1 and the various mutants as detected by immunofluorescence. The proportion of non-permeabilized cells showing surface expression was: AFF-1-FLAG (negative control; 0%, n=2), Fsx1-FLAG (3.9%, n=1), Fsx1-ΔFL→AG_4A-FLAG (4.4%, n=1), Fsx1-ΔDIV→EFF-1 stem-FLAG (2.6%, n=1), Fsx1ΔTMs→EFF-1 TM-FLAG (3.3%, n=2), Fsx1ΔTMs→GPI-FLAG (32.6%, n=3), AtHAP2ΔTM→GPI-FLAG (22.8%, n=2). Total number of cells with red nuclei counted n ≥850 for each experimental condition; the percentage of surface expression was counted by at least two different observers. Another group of transfected BHK cells in parallel were fixed, permeabilized and stained with anti-FLAG antibody. Permeabilized staining showed the main distribution in the cytoplasm (endoplasmic reticulum) of Fsx1 WT, Fsx1 mutants and AtHAP2ΔTM→GPI mutant. *C. elegans* AFF-1 tagged with FLAG at the C terminus (cytoplasmic tail) worked as a negative control for non-permeabilized staining. Scale bars, 10 μm.

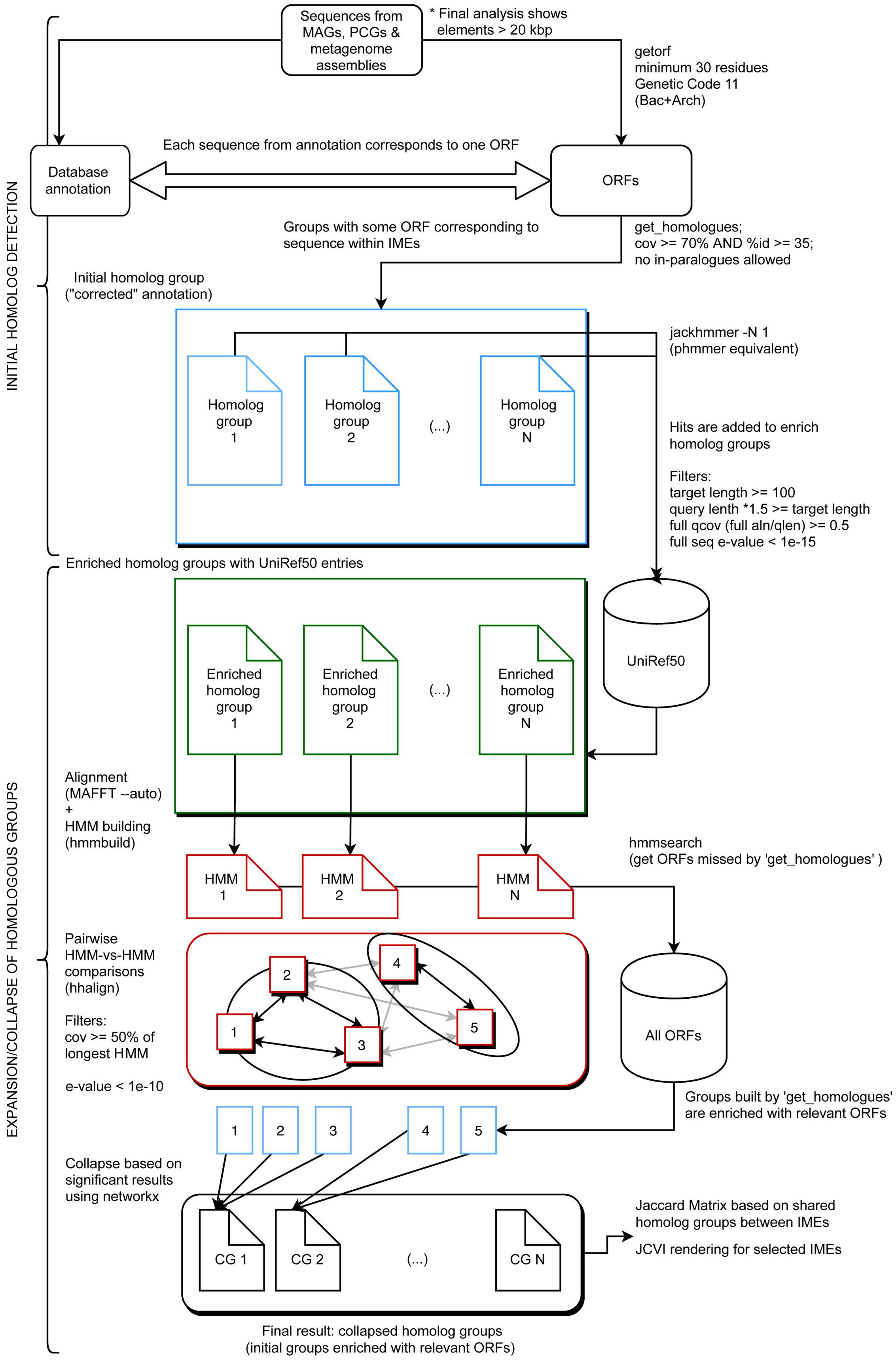
Sequences from MAGs, PCGs & metagenome assemblies
* Final analysis shows elements > 20 kbp
getorf
minimum 30 residues
Genetic Code 11
(Bac+Arch)
Database annotation
Each sequence from annotation corresponds to one ORF
ORFs
INITIAL HOMOLOG DETECTION
Groups with some ORF corresponding to sequence within IMEs
get_homologues;
cov >= 70% AND %id >= 35;
no in-paralogues allowed
Initial homolog group
("corrected" annotation)
jackhmmer -N 1
(phmmer equivalent)
Homolog group 1
Homolog group 2
(...)
Homolog group N
Hits are added to enrich homolog groups
Filters:
target length >= 100
query lenth *1.5 >= target length
full qcov (full aln/qlen) >= 0.5
full seq e-value < 1e-15
Enriched homolog groups with UniRef50 entries
UniRef50
Enriched homolog group 1
Enriched homolog group 2
(...)
Enriched homolog group N
EXPANSION/COLLAPSE OF HOMOLOGOUS GROUPS
Alignment
(MAFFT --auto)
+
HMM building
(hmmbuild)
hmmsearch
(get ORFs missed by 'get_homologues')
HMM 1
HMM 2
HMM N
Pairwise
HMM-vs-HMM
comparisons
(hhalign)
Filters:
cov >= 50% of longest HMM
e-value < 1e-10
All ORFs
Groups built by 'get_homologues' are enriched with relevant ORFs
Collapse based on significant results using networkx
CG 1
CG 2
(...)
CG N
Jaccard Matrix based on shared homolog groups between IMEs
JCVI rendering for selected IMEs
Final result: collapsed homolog groups
(initial groups enriched with relevant ORFs)

Supplementary Fig. 8 Bioinformatics workflow for IMEs.

The general workflow is divided into two parts. First, we re-annotated the ORFs of each IME and searched for potential homologs between them. Then we enriched these initial groups by searching the UniRef50 database and generated new HMM profiles. With these new HMMs we searched again within each IME to capture any potentially missing homolog. Finally, we performed HMM vs HMM[13] search to collapse similar groups into one. CG1, CG2...CGN are final collapsed homologous groups which are the basis for IME clustering, synteny conservation and gene content analyses shown in Supplementary Fig. 9, Fig. 8 and Supplementary Table 3, respectively.

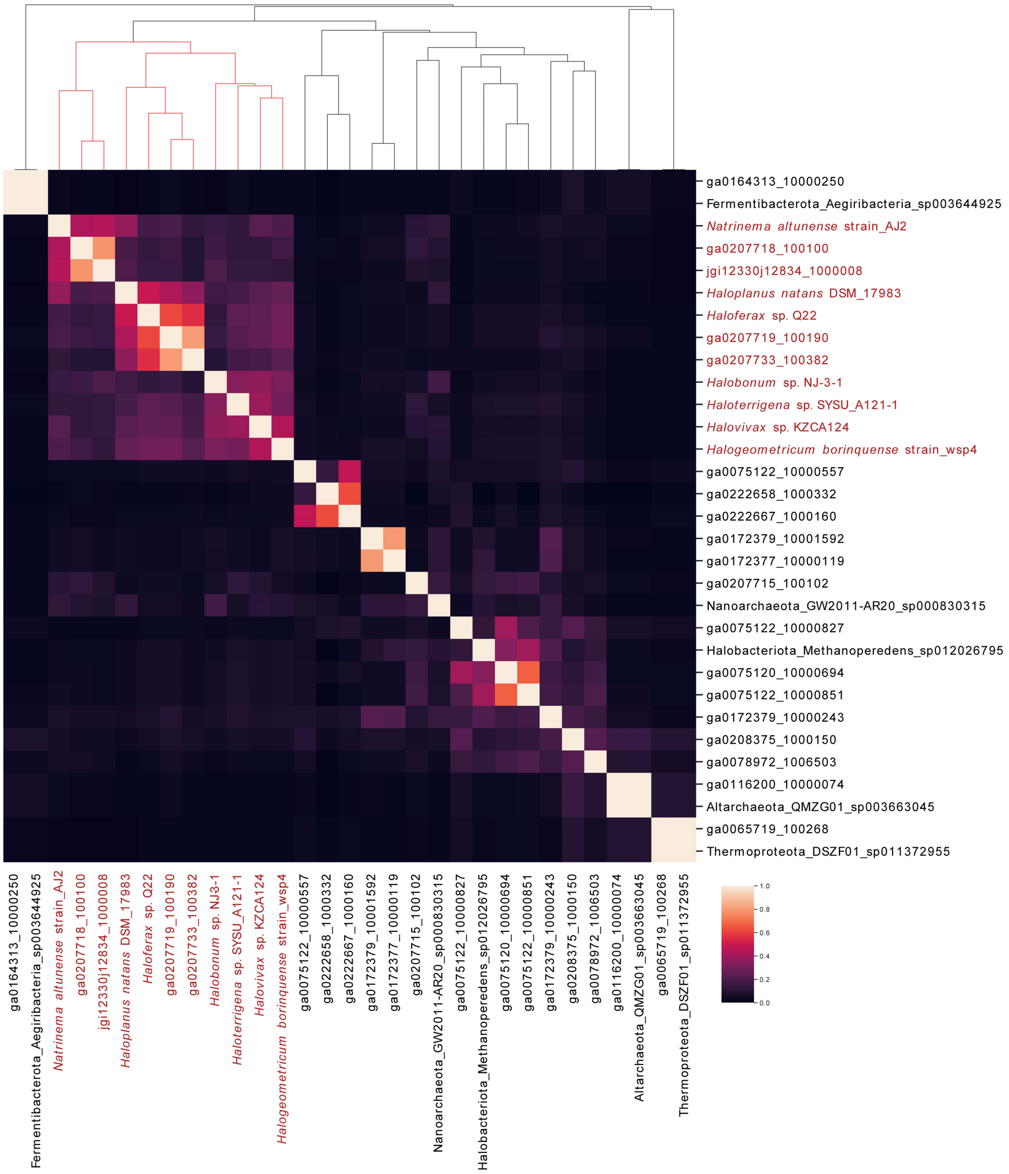

Supplementary Fig. 9 Clustering of IMEs from complete genomes, MAGs and metagenomic contigs based on gene content.

A distance metric based on the sharing of homologous genes between all IMEs was computed (see Methods). Then a pairwise distance matrix was built to perform hierarchical clustering. There is a clear cluster, marked in red, which contains all PCG IMEs and the DNA contig containing the crystallized Fsx1 (jgi12330j12834_1000008). This cluster of 11 IMEs was used for the synteny conservation analysis shown in Fig. 8.

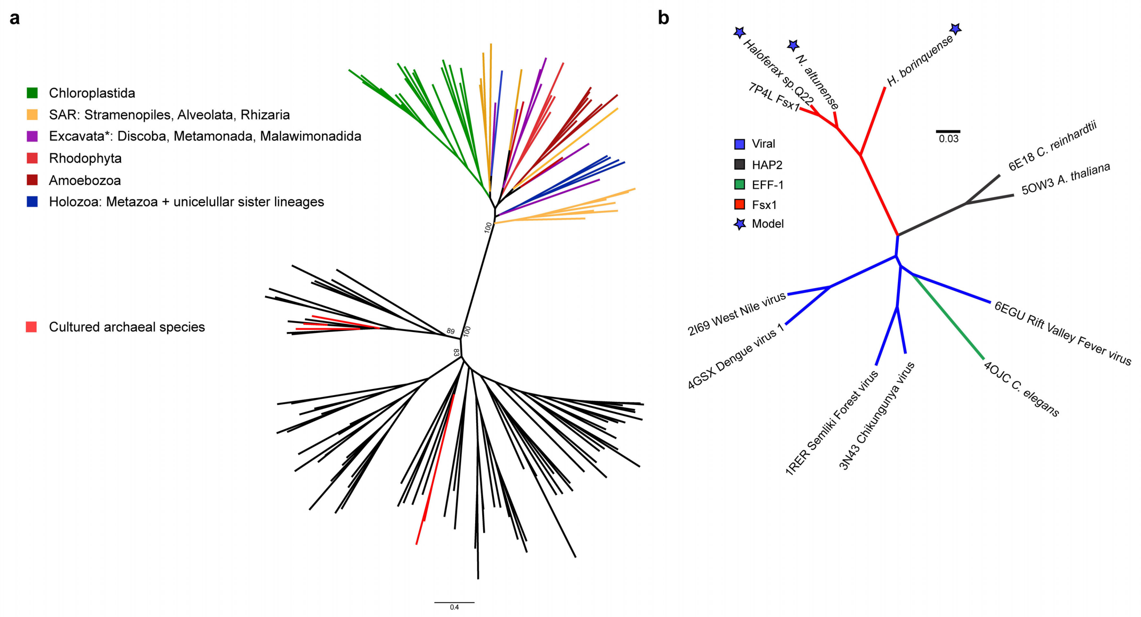

Supplementary Fig. 10 Unrooted phylogenetic tree of Archaea/Eukarya fusexins and structure-based tree of all fusexins.

a Unrooted phylogenetic sequence tree of archaeal and eukaryotic fusexins. Archaeal Fsx1s from cultivated species are shown in red; eukaryotic HAP2 major clades are indicated in colors. Relevant branch support values are indicated, raw tree available in Supplementary Data 5.

b Monomer models based on the $Fsx1_E$ crystal structure and experimental structures of other fusexins were compared using flexible structural alignment to build a minimum evolutionary tree. Scale bar represents distance as 1-TMscore, which supports their homology.

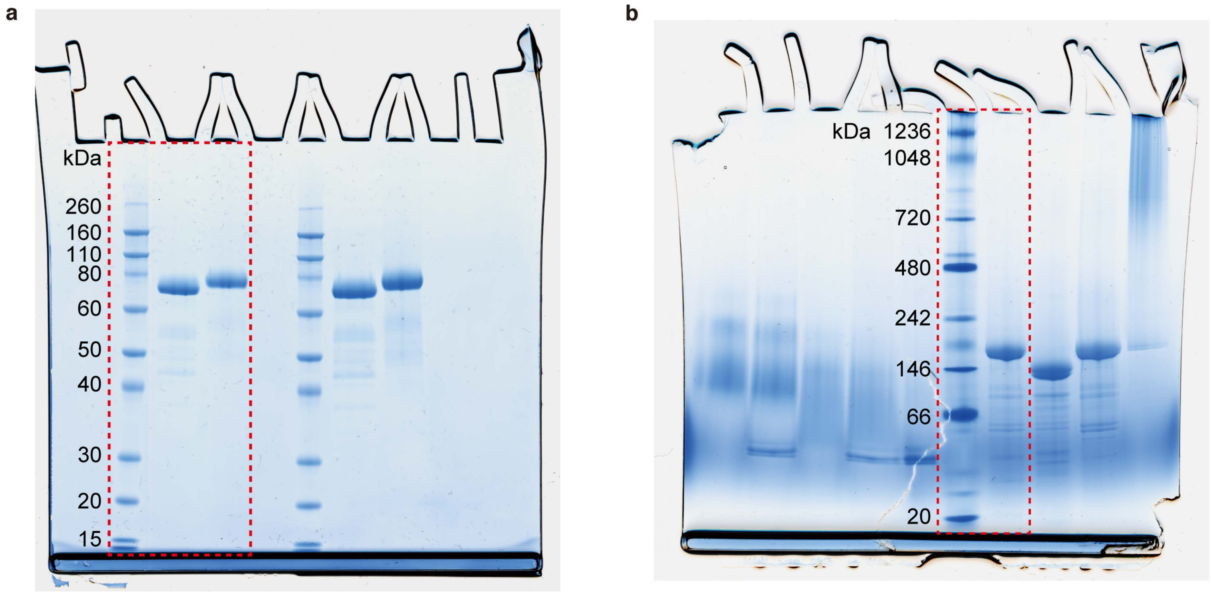

Supplementary Fig. 11 Uncropped gel images from Supplementary Fig. 2.

a SDS-PAGE gel stained with Coomassie G-250. The section indicated by a red dashed square is used in Supplementary Fig. 2a**.** n=2.

b BN-PAGE gel stained with Coomassie G-250. The section indicated by a red dashed square is used in Supplementary Fig. 2b. n=2.

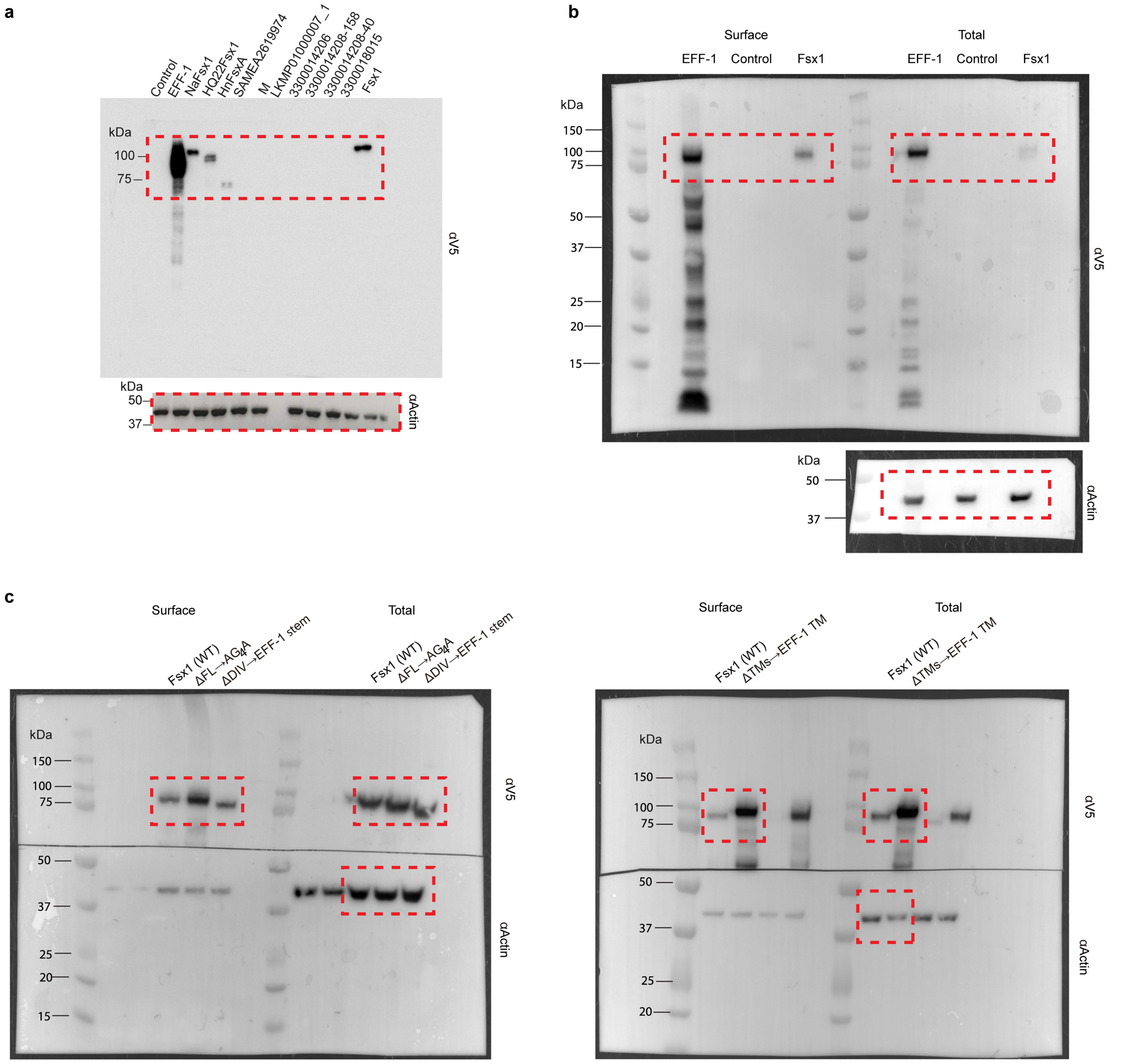

Supplementary Fig. 12 Uncropped Western blot images from Supplementary Fig. 1c and Fig. 5d, e.

a Western blot probed with anti-V5 (upper) and anti-actin (lower) antibodies. The sections indicated by red dashed squares are used in Supplementary Fig. 1c. n=3.

b Western blot probed with anti-V5 (upper) and anti-actin (lower) antibodies. The sections indicated by red dashed squares are used in Fig. 5d. n=3.

c Western blots probed with anti-V5 (upper) and anti-actin (lower) antibodies. The sections indicated by red dashed squares are used in Fig. 5e. n=3.

Supplementary Table 1. Fsx1s in genomes with assigned taxonomy

Fsx1s in Pure Culture Genomes (PCGs)

Sequence ID	Species	Taxonomy
WP_058826362.1	*Haloferax* sp. Q22	Archaea › Euryarchaeota › Stenosarchaea group › Halobacteria › Haloferacales › Haloferacaceae
WP_144240185.1	*Natrinema altunense* (AJ2)	Archaea › Euryarchaeota › Stenosarchaea group › Halobacteria › Natrialbales › Natrialbaceae
ELY83688.1	*Natrinema altunense* JCM12890)	Archaea › Euryarchaeota › Stenosarchaea group › Halobacteria › Natrialbales › Natrialbaceae
WP_157573584.1	*Haloplanus natans* DSM 17983	Archaea › Euryarchaeota › Stenosarchaea group › Halobacteria › Haloferacales › Haloferacaceae
WP_174701778.1	*Haloterrigena* sp. SYSU A121-1	Archaea › Euryarchaeota › Stenosarchaea group › Halobacteria › Natrialbales › Natrialbaceae
WP_179268568.1	*Halobonum* sp. NJ-3-1	Archaea › Euryarchaeota › Stenosarchaea group › Halobacteria › Haloferacales › Halorubraceae
WP_163487151.1	*Halogeometricum borinquense* strain wsp4	Archaea › Euryarchaeota › Stenosarchaea group › Halobacteria › Haloferacales › Haloferacaceae
WP_207587115.1	*Halovivax* sp. KZCA124	Archaea › Euryarchaeota › Stenosarchaea group › Halobacteria › Natrialbales › Natrialbaceae

Fsx1s in Metagenome-Assembled Genomes (MAGs)

Sequence ID	Assigned taxon	Taxonomy
MGYP000598426430	Halobacteriales	Archaea › Euryarchaeota › Stenosarchaea group › Halobacteria › Halobacteriales
LKMP01000007_1	Nanohaloarchaea archaeon B1-Br10_U2g21	Archaea › Euryarchaeota › Stenosarchaea group › Candidatus Nanohaloarchaeota
RLG58774.1	Candidatus Geothermarchaeota B85_G16	Archaea ›TACK group › Candidatus Geothermarchaeota
RLI53188.1	Candidatus Thorarchaeota archaeon	Archaea › Asgard group › Candidatus Thorarchaeota
RKX41251.1	Thermotogae bacterium	Bacteria › Thermotogae
RKZ11204.1	Candidatus Fermentibacteria bacterium	Bacteria › Candidatus Fermentibacteria
RLG94066.1	Candidatus Bathyarchaeota archaeon	Archaea ›TACK group › Candidatus Bathyarchaeota
AJF63093.1	archaeon GW2011_AR20	Archaea ›unclassified
HEX32987.1	Candidatus Aenigmarchaeota archaeon	Archaea › DPANN group › Candidatus Aenigmarchaeota
HDD44259.1	Candidatus Desulfofervidus auxilii	Bacteria › Proteobacteria › Deltaproteobacteria › Candidatus Desulfofervidaceae
HDI72891.1	Candidatus Altiarchaeales archaeon	Archaea › DPANN group › Candidatus Altiarchaeota › Candidatus Altiarchaeales
HHR27186.1	Candidatus Bathyarchaeota archaeon	Archaea ›TACK group › Candidatus Bathyarchaeota
NJD53946.1	Candidatus Methanoperedens sp.	Archaea › Euryarchaeota › Stenosarchaea group › Methanomicrobia › Methanosarcinales › Cand. Methanoperedenaceae
NOZ47386.1	Chlorobi bacterium	Bacteria › Chlorobi
HGF63239.1	Candidatus Micrarchaeota archaeon	Archaea › DPANN group › Candidatus Micrarchaeota
HID09282.1	Candidatus Micrarchaeota archaeon	Archaea › DPANN group › Candidatus Micrarchaeota

Supplementary Table 2. Fsx1$_E$ data collection and refinement statistics

**Data collection**	
Space group	*C*2 (5)
Cell dimensions	
a, *b*, *c* (Å)	262.51, 111.33, 68.51
α, *β*, *γ* (°)	90, 100.709, 90
Wavelength (Å)	1.005
Resolution range (Å)	67.3-2.3 (2.38-2.30)§
Unique reflections	85618 (8536)
Multiplicity	4.2 (4.4)
Completeness (%)	99.5 (99.9)
Mean I / σI	11.4 (1.4)
Wilson B-factor	42.8
R_{merge}	0.100 (1.218)
R_{meas}	0.115 (1.385)
R_{pim}	0.055 (0.651)
$CC_{1/2}$	1.0 (0.58)
CC*	1.0 (0.86)
**Refinement**	
Reflections used in refinement	85574 (6092)**
Reflections used for R_{free}	2012 (149)
R_{work}	0.199 (0.303)
R_{free}	0.243 (0.377)
Number of non-H atoms	11733
macromolecules / ligand / solvent	11051 / 66 / 616
Protein residues	1432
RMS	
bonds (Å)	0.004
angles (°)	0.58
Ramachandran favored / allowed / outliers (%)	98.9 / 1.1 / 0.0
Rotamer outliers (%)	0.2
Clashscore	2.5
Average B-factor	53.4
macromolecules / ligand / solvent	53.5 / 58.8 / 50.0

§ Values in parenthesis are for the highest resolution shell

** The highest resolution shell used in refinement included reflections between 2.36 and 2.30 Å

Supplementary Table 3. Most common arCOGs from 11 IMEs[a]

Collapsed Group Name	IMEs count[b]	arCOG count[c]	arCOG	Category	Annotation
CG_2	11	22	arCOG00280, arCOG00285, arCOG06224	L	HerA helicase
CG_17	10	21	arCOG01241, arCOG01248, arCOG01250	X	XerC XerD/XerC family integrase
41684	10	3	arCOG01680, arCOG02808, arCOG04362	K	Transcriptional regulator containing HTH domain
43214	9	9	arCOG08903	S	Uncharacterized protein
CG_1	8	8	arCOG12186	S	Uncharacterized protein
CG_2	7	7	arCOG04816	U	TraG/TraD/VirD4 family enzyme, ATPase
43797	7	7	arCOG12187	S	Uncharacterized membrane protein
43810	7	7	arCOG10296	S	Uncharacterized membrane protein
43833	7	7	arCOG08907	S	Uncharacterized protein
43868	7	7	arCOG10381	S	Uncharacterized protein
CG_2	6	7	arCOG00467	L	Cdc6-related protein, AAA superfamily ATPase
CG_2	6	6	arCOG07496	U	VirB4, Type IV secretory pathway
42763	6	6	arCOG06216	K	Transcriptional regulator
CG_2	5	5	arCOG01308	O	ATPase of the AAA+ class , CDC48 family
CG_21	3	3	arCOG07871	K	Helicase
CG_2	2	2	arCOG00439	L	ATPase involved in replication control
CG_2	2	2	arCOG03779	V	GTPase subunit of restriction endonuclease
CG_2	2	2	arCOG05935	R	Helicase of FtsK superfamily
CG_21	2	2	arCOG00878	V	restriction-modification related helicase
CG_9	1	1	arCOG03600	E	Transglutaminase-like cysteine protease
CG_21	1	1	arCOG04818	K	Superfamily II DNA/RNA helicase, SNF2 family

a. Collapsed Homology Groups (Supplementary Fig. 8) corresponding to the 11 IME clusters (Supplementary Fig. 9) were analyzed using HMMER against the arCOGs database. Collapsed Homology Groups with zero identified arCOGs are not shown. Full dataset with results is in Supplementary Data 3.

b. Number of IMEs where the indicated arCOGs are present.

c. Total number of ORFs belonging to arCOGs in the next column identified in the set of 11 IMEs.

Supplementary Table 4. IMEs carrying the *fsx1* gene in completely sequenced genomes

Species with *fsx1*	NCBI TaxID	PATRIC ID	sequence ID[a]	ME start k-mer[b]	ME end k-mer[b]	Length ME k-mer	ME start CG[c]	ME end CG[c]	Length ME CG	Species w/o *fsx1*[d]	NCBI TaxID	PATRIC ID	ANI[e]
Haloplanus natans DSM 17983	926690	926690.3	ATYM01000002	1422500	1526500	104001	1422548	1535558	113011	*Haloplanus* sp. CBA1112	1547898	1547898.3	88.0
Natrinema altunense strain AJ2	222984	222984.5	JNCS01000001	496500	593500	97001	497005	591892	94888	*Natrinema altunense* strain 4.1R	222984	222984.10	98.0
Halobonum sp. NJ-3-1	2743089	2743089.3	CP058579	1918000	2078500	160501	1906722	2078630	171909	*Halobonum* sp. Gai3-2	2743090	2743090.3	85.0
Haloferax sp. Q22	1526048	1526048.3	LOEP01000012	1	56511	56511	1	56511	56511	*Haloferax gibbonsii* strain LR2-5	35746	35746.12	94.8
Haloterrigena sp. SYSU A121-1	2496101	2496101.3	JABURA010000001	1761500	1860500	99001	1761690	1860499	98810	*Haloterrigena turkmenica* DSM 5511	543526	543526.13	92.4
Halogeometricum borinquense strain wsp4	60847	60847.21	CP048739	2797000	2827000	30001	2763703	2853374	89672	*Halogeometricum borinquense* strain wsp3	60847	60847.22	99.6
Halovivax sp. KZCA124	2817025	2817025.3	NZ_CP071597	3085000	3179500	94501	--[f]	--	--	--	--	--	--

a. Genomic contig carrying the IME.

b. Sequence coordinates of the start and end of the IME identified by k-mer spectrum.

c. Sequence coordinates of the start and end of the IME identified by comparative genomics (CG).

d. Closest species not carrying the *fsx1* gene with a completely sequenced genome (used for CG analysis).

e. Average nucleotide identity between the complete genomes of compared species.

f. No species with a completely sequenced genome was similar enough to *Halovivax* sp. KZCA124 to compute an accurate estimate of IME's insertion sites with CG.

Supplementary Table 5. Synthesized archaeal fusexin genes for fusogenic tests in mammalian cells

Synthesized plasmids	Accession number/Sequence	Species/Assembly source
pBPT01	WP_007110832	*Natrinema altunense*
pBPT02	WP_058826362	*Haloferax* sp.Q22
pBPT03	WP_049937247	*Haloplanus natans*
pBPT04	SAMEA2619974_10776_4	MAG
pBPT05	LKMP01000007_1	Nanohaloarchaea archaeon B1-Br10_U2g21 LB-BRINE-C121
pBPT06	3300014206-Ga0172377-10000119-870930-129	unassembled metagenome
pBPT07	3300014208-Ga0172379-10000243-871512-158	unassembled metagenome
pBPT08	3300014208-Ga0172379-10001592-871560-40	unassembled metagenome
pBPT10	3300018015-Ga0187866_1000629|915963_9	unassembled metagenome
pBPT11	3300000868-JGI12330J12834-1000008-299010-8, **Fsx1**	unassembled metagenome

Supplementary Table 6. Plasmids used in this study

Plasmid name	Description	Use	Source
pLJFX11B	pLJ6-Fsx1$_E$	For SAXS and SEC-MALS (Supplementary Fig. 2)	this study, amplified from pBPT11
pLJFX11B_T369C	pLJ6-Fsx1$_E$-T369C mutant	For crystallographic study (Figs. 2-3; Supplementary Fig. 2)	this study, amplified from pBPT11
pBPT01	*Natrinema altunense fsx1* synthesized into pGene/V5-His	Inducible expression in mammalian cells (Supplementary Fig.1)	this study
pBPT02	*Haloferax* sp. Q22 *fsx1* synthesized into pGene/V5-His	Inducible expression in mammalian cells (Supplementary Fig.1)	this study
pBPT03	*Haloplanus natans fsx1* synthesized into pGene/V5-His	Inducible expression in mammalian cells (Supplementary Fig. 1)	this study
pBPT04	SAMEA2619974 synthesized into pGene/V5-His	Inducible expression in mammalian cells (Supplementary Fig. 1)	this study
pBPT05	LKMP01000007_1 synthesized into pGene/V5-His	Inducible expression in mammalian cells (Supplementary Fig. 1)	this study
pBPT06	3300014206 synthesized into pGene/V5-His	Inducible expression in mammalian cells (Supplementary Fig. 1)	this study
pBPT07	3300014208-158 synthesized into pGene/V5-His	Inducible expression in mammalian cells (Supplementary Fig. 1)	this study
pBPT08	3300014208-40 synthesized into pGene/V5-His	Inducible expression in mammalian cells (Supplementary Fig. 1)	this study
pBPT10	3300018015 synthesized into pGene/V5-His	Inducible expression in mammalian cells (Supplementary Fig. 1)	this study
pBPT11	*fsx1* synthesized into pGene/V5-His	Inducible expression in mammalian cells (Supplementary Fig. 1)	this study
pGene/V5-His	pGene/V5-His	GeneSwitch™ inducible Mammalian Expression	INVITROGEN
pSwitch	pSwitch	Regulatory vector for Mifepristone induction	INVITROGEN
pOA34	pGene::EFF-1-V5	*C. elegans eff-1* fused to a C-terminal V5 tag (EFF-1-V5) in pGene	Avinoam et al., 2011[14]
pCI H2B-RFP	pCI::H2B-RFP	A CAG promoter (CMV immediate early enhancer and chicken beta actin promoter) and IRES controlled Histone2B-mRFP1 reporter.	Addgene plasmid # 92398[15]
pCI H2B-GFP	pCI::H2B-GFP	A CAG promoter (CMV immediate early enhancer and chicken beta actin promoter) and IRES controlled Histone2B-EGFP reporter.	Addgene plasmid # 92399[15]
pCAGIG	pCAGIG	A CAG promoter (CMV immediate early enhancer and chicken beta actin promoter) and IRES controlled EGFP reporter.	Addgene plasmid # 11159[16]
pNB25	pCAGIGnes	Intermediate construct to create pNB32	this study
pNB32	pCI::GFPnes	Content-mixing, Fig. 4a-c	this study
pRFPnes	DsRed2 with a nuclear export signal	Content-mixing, Fig. 4a-c	Avinoam et al., 2011[14]
pXL27	pCI::Fsx1-V5::H2B-RFP	Content-mixing, Fig. 4a-c	this study
pXL28	pCI::Fsx1-V5::H2B-GFP	Content-mixing, Fig. 4a-c; live imaging of fusion, Fig. 4g	this study
pXL29	pCI::AtHAP2-V5::H2B-RFP	Content-mixing, Fig. 4a-c;<br>Multinucleation assay (Supplementary Fig. 1)	this study
pXL30	pCI::AtHAP2-V5::H2B-GFP	Content-mixing, Fig. 4a-c;<br>Multinucleation assay (Supplementary Fig. 1)	this study
pXL49	pCI::Fsx1-V5::GFPnes	Content-mixing, Fig. 4d-e	this study
pNB34	pCI::EFF-1-V5::GFPnes	Content-mixing, Fig. 4d-e	this study
pXL68	pCI::VSV-G::GFPnes	Content-mixing, Fig. 4d-e	this study
pOA19	pCAGGS::EFF-1-V5	Surface biotinylation of EFF-1 (Fig. 5d)	Avinoam et al., 2011[14]
pXL50	pCAGGS::Fsx1-V5	Surface biotinylation of Fsx1 (Fig. 5d)	this study
myr-mCherry	myr-mCherry	mCherry linked to a myristoylated and palmitoylated peptide, live imaging of fusion (Fig. 4g)	Dunsing et al., Sci. Rep. 2018[17]
myr-EGFP	myr-EGFP	EGFP linked to a myristoylated and palmitoylated peptide (Supplementary Fig. 1)	Dunsing et al., Sci. Rep. 2018[17]
pXL21	pCI::NaFsx1-V5::H2B-RFP	Multinucleation assay (Supplementary Fig. 1)	this study
pXL22	pCI::NaFsx1-V5::H2B-GFP	Multinucleation assay (Supplementary Fig. 1)	this study
pXL23	pCI::HQ22Fsx1-V5::H2B-RFP	Multinucleation assay (Supplementary Fig. 1)	this study

Plasmid name	Description	Use	Source
pXL24	pCI::HQ22Fsx1-V5::H2B-GFP	Multinucleation assay (Supplementary Fig. 1)	this study
pXL25	pCI::HnFsx1-V5::H2B-RFP	Multinucleation assay (Supplementary Fig. 1)	this study, subcloned from pBPT03 with modification of complete signal peptide
pXL26	pCI::HnFsx1-V5::H2B-GFP	Multinucleation assay (Supplementary Fig. 1)	this study, subcloned from pBPT03 with modification of complete signal peptide
pXL57	pCI::Fsx1-ΔFL-AG$_4$A::GFPnes	Content-mixing, Fig. 5a-c	this study
pXL58	pCI::Fsx1-ΔFL-AG$_4$A::H2B-RFP	Content-mixing, Fig. 5a-c	this study
pXL63	pCI::Fsx1-ΔDIV-EFF-1-stem::H2B-RFP	Content-mixing, Fig. 5a-c	this study
pXL64	pCI::Fsx1-ΔDIV-EFF-1-stem::GFPnes	Content-mixing, Fig. 5a-c	this study
pXL108	pCAGGS::Fsx1-ΔDIV-EFF-1-stem	Surface biotinylation of Fsx1-ΔDIV-EFF-1-stem mutant (Fig. 5e)	this study
pXL82	pCI::Fsx1-WT-FLAG-3TMs::H2B-RFP	Surface expression tests, FLAG tag inserted before the first TM segment of Fsx1 (Fig. 5f)	this study
pXL86	pCI::Fsx1-ΔFL-AG$_4$A-FLAG-3TMs::H2B-RFP	Surface expression tests, FLAG tag inserted before the first TM segment of Fsx1-ΔFL-AG$_4$A mutant (Fig. 5f)	this study
pXL92	pCI::Fsx1-ΔDIV-EFF-1-stem-FLAG-3TMs::H2B-RFP	Surface expression tests, FLAG tag inserted before the first TM segment of Fsx1-ΔDIV-EFF-1-stem mutant (Fig. 5f)	this study
pOA20	pCAGGS::AFF-1-FLAG	*C. elegans aff-1* fused to a C-terminal FLAG tag (AFF-1-FLAG) in pCAGGS	Avinoam et al., 2011[14]
pXL100	pCI::AFF-1-FLAG::H2B-RFP	Surface expression tests of AFF-1 (Fig. 5f)	this study
pXL106	pCAGGS-Fsx1-ΔFL-AG$_4$A	Surface biotinylation of Fsx1-ΔFL-AG$_4$A mutant (Fig. 5e)	this study
pXL123	pCAGGS-Fsx1ΔTMs-EFF-1 TM	Surface biotinylation of Fsx1ΔTMs-EFF-1 TM mutant (Fig. 5e)	this study
pXL119	pCI::Fsx1ΔTMs-EFF-1 TM::H2B-RFP	Content-mixing, Fig. 5a-c; Surface expression tests, FLAG tag inserted before the EFF-1 TM region (Fig. 5f)	this study
pXL120	pCI::Fsx1ΔTMs-EFF-1 TM::GFPnes	Content-mixing, Fig. 5a-c	this study
pXL115	pCI::Fsx1ΔTMs-GPI::H2B-RFP	Content-mixing, Fig. 5a-c; Surface expression tests, FLAG tag inserted before the GPI (Fig. 5f)	this study
pXL116	pCI::Fsx1ΔTMs-GPI::GFPnes	Content-mixing, Fig. 5a-c	his study
pXL117	pCI::AtHAP2ΔTM-GPI::H2B-RFP	Content-mixing, Fig. 5a-c; Surface expression tests, FLAG tag inserted before the GPI (Fig. 5f)	this study
pXL118	pCI::AtHAP2ΔTM-GPI::GFPnes	Content-mixing, Fig. 5a-c	this study

Supplementary Table 7. Primers used in this study

Primer name	Sequence	Description
SNFX11_F	CGTAGCTGAAACCGGTGATTCAATCACGTATAACTCTGG	Forward primer for cloning Fsx1$_E$ and T369C mutant into pLJ6 with AgeI
SNFX11_R	GGTGATGGTGCTCGAGGGAACCAGAACCTCCGAA	Reverse primer for cloning Fsx1$_E$ and T369C mutant into pLJ6 with XhoI
SNFX11_T369C_F	ACTGTAtgcGCCACCGTTGAGAATGTC	Forward primer for T369C mutant
SNFX11_T369C_R	GGTGGCgcaTACAGTTCCCTCATCTCCCTC	Reverse primer for T369C mutant
seq_up	GCTGGTTGTTGTGCTGTCTCATC	Sequencing primer for pLJ6
seq_down	CACCAGCCACCACCTTCTGATAG	Sequencing primer for pLJ6
LXH1	GATGGTGCGATTGCGGAT	Sequencing primer for pBPT01
LXH2	CCTACGAGAATGGGCAGA	Sequencing primer for pBPT01
LXH3	TTGCTGGCAGAGAAATGA	Sequencing primer for pBPT02
LXH4	TGATGTACCCCGAGTTCA	Sequencing primer for pBPT02
LXH5	GGATGAAATCTTCAGAAC	Sequencing primer for pBPT03
LXH6	ACTGTCTCGAAGCCGGTT	Sequencing primer for pBPT03
LXH7	CAAAATCACCCTCACATC	Sequencing primer for pBPT04
LXH8	CCTACAATATTAAGTTGTG	Sequencing primer for pBPT04
LXH9	TGAGTCTGAATGGATTAT	Sequencing primer for pBPT05
LXH10	TAGGACTACAGCGAAAAT	Sequencing primer for pBPT05
LXH11	TTGGGGAGGAAATGTAAA	Sequencing primer for pBPT06
LXH12	TAGAAGAATAAATATTCC	Sequencing primer for pBPT06
LXH13	TCCTCTTCCCTCGGAGAA	Sequencing primer for pBPT07
LXH14	CCTACTCAGGTAACGTAA	Sequencing primer for pBPT07
LXH15	CAGTAACAATAAATGGTG	Sequencing primer for pBPT08
LXH16	CAGAAGAATAAACATTCC	Sequencing primer for pBPT08
LXH17	AACAATAGGACAAGCAAA	Sequencing primer for pBPT10
LXH18	ACCAAAAATATTGTCTGC	Sequencing primer for pBPT10
LXH19	AGCATACATAGACAACCC	Sequencing primer for pBPT11, Fsx1
LXH20	ACGTCGATGCCGGAGAAA	Sequencing primer for pBPT11, Fsx1
pGene FW	CTGCTCAACCTTCCTATC	pGene backbone sequencing forward primer
pGene REV	TTAGGAAAGGACAGTGGGAGTG	pGene backbone sequencing reverse primer
PCA-5	GGTTCGGCTTCTGGCGTGTGACC	pCI::H2B-RFP/H2B-GFP/GFPnes backbones sequencing forward primer
IRES-REV	GCATTCCTTTGGCGAGAG	pCI::H2B-RFP/H2B-GFP/GFPnes backbones sequencing reverse primer
pCAGGS FW	GCAACGTGCTGGTTGTTGTGCTGTC	pCAGGS backbone sequencing forward primer
pCAGGS RV	TCCCATATGTCCTTCCGAGTGA	pCAGGS backbone sequencing reverse primer
LXH24	CGGGGTACCATGAGACGTGCAGCATTG	Forward primer for cloning Fsx1 and its mutants into pCAGGS vector with KpnI
LXH42	CTAGCTAGCGGTACCATGAGACGTGCAGCATTGATT	Forward primer for cloning Fsx1 into pCI::H2B-RFP/H2B-GFP/GFPnes vectors with NheI and KpnI
LXH44	CTAGCTAGCGGTACCATGGAACCGCCGTTTGAGTGG	Forward primer for cloning EFF-1 into pCI::GFPnes vector with NheI and KpnI
LXH45	CTAGCTAGCGGTACCATGGTGAACGCGATTTTAATG	Forward primer for cloning AtHAP2 into pCI::H2B-RFP/GFP vectors with NheI and KpnI
LXH79	TCCCCCGGGCTAATGGTGATGGTGATGATGACC	Reverse primer for cloning fusexins into pCI vectors with SmaI which binds to 6xHis tag
LXH81	CTAGCTAGCTCAATGGTGATGGTGATGATGACC	Reverse primer for cloning Fsx1 and its mutants into pCAGGS vector with NheI
LXH111	CTAGCTAGCATGAAGTGCCTTTTGTACTTAG	Forward primer for cloning VSV-G into pCI::GFPnes vector with NheI

Primer name	Sequence	Description
LXH112	TCCCCCGGGTTACTTTCCAAGTCGGTTCATC	Reverse primer for cloning VSV-G into pCI::GFPnes vector with SmaI
LXH39	CTAGCTAGCGGTACCATGCGGGCGGTGTCTGATTTC	Forward primer for cloning NaFsx1 into pCI::H2B-RFP/GFP vectors with NheI and KpnI
LXH40	CTAGCTAGCGGTACCATGAAAAACGGGTTGAAGGCC	Forward primer for cloning HQ22Fsx1 into pCI::H2B-RFP/GFP vectors with NheI and KpnI
LXH134	CTAGCTAGCATGGTGAAACGAGTGGGTAATTGTTGG AAGGCCTCAGTAGCGGCATTCTTCCTTCTCATGTTCACTGCATTT	Forward primer for cloning HnFsx1 into pCI::H2B-RFP/GFP vectors with NheI; containing modification of complete signal peptide
LXH107	GCGGAAGGTACAGCAGGTACGCCGGTGGAGGTGGAGCTGATTACGA GATCTATTGTTT	Forward primer for cloning downstream of Fsx1-ΔFL-AG$_4$A mutant by overlap PCR
LXH108	AAACAATAGATCTCGTAATCAGCTCCACCTCCACCGGCGTACCTGCTG TACCTTCCGC	Reverse primer for cloning upstream of Fsx1-ΔFL-AG$_4$A mutant by overlap PCR
LXH101	ACCGGTATCCAGCAGGAAATCGATCTTGTT	Forward primer 1 for cloning Fsx1-ΔDIV-EFF-1-stem by overlap PCR
LXH102	AACAAGATCGATTTCCTGCTGGATACCGGT	Reverse primer 1 for cloning Fsx1-ΔDIV-EFF-1-stem by overlap PCR
LXH103	ATGATTGCTACGGATCAGGACGATGATTCA	Forward primer 2 for cloning Fsx1-ΔDIV-EFF-1-stem by overlap PCR
LXH104	TGAATCATCGTCCTGATCCGTAGCAATCAT	Reverse primer 2 for cloning Fsx1-ΔDIV-EFF-1-stem by overlap PCR
LXH128	TGTTCGGAGGTTCTGGTTCCGACTACAAGGACGACGATGACAAAGGA GATCTGCTTAC	Forward primer for cloning downstream of Fsx1-WT/mutants-FLAG by overlap PCR
LXH129	GGAACCAGAACCTCCGAACA	Reverse primer for cloning upstream of Fsx1-WT/mutants-FLAG by overlap PCR
LXH135	CTAGCTAGCATGGTACTGTGGCAATGGTCAATAG	Forward primer for cloning *C. elegans* AFF-1-FLAG into pCI::H2B-RFP vector with NheI
LXH136	TCCCCCGGGTTATTTGTCATCGTCGTCCTTGTAGTC	Reverse primer for cloning *C. elegans* AFF-1-FLAG into pCI::H2B-RFP vector with SmaI
LXH140	GACGGGTAGTACCTGAAGTGGTTCCACTTCCTTTATTTGGAGAACCT CCTTTGTCATCGTCGTCCTTGTAGTCGGAACCAGAACCTCCG	Reverse primer 1 for fusing GPI signal from DAF to Fsx1
LXH141	TCCCCCGGGCTAAGTCAGCAAGCCCATGGTTACTAGCGTCCCAAGC AAACCTGTCAACGTGAAACACGTGTGCCCAGATAGAAGACGGGTAG TACCTGAAGTG	Reverse primer 2 for fusing GPI signal from DAF to Fsx1; Reverse primer for cloning downstream of AtHAP2ΔTM-GPI mutant by overlap PCR
LXH142	AATACGTTTGCTTAAGCTGGGGTGGAAGCGACTACAAGGACGACGA T	Forward primer for cloning downstream of AtHAP2ΔTM-GPI mutant by overlap PCR
LXH143	CCAGCTTAAGCAAACGTATT	Reverse primer for cloning upstream of AtHAP2ΔTM-GPI mutant by overlap PCR
LXH144	GGAGGTTCTGGTTCCGACTACAAGGACGACGATGACAAAATTGTTG TGTATCTC	Forward primer for cloning downstream of Fsx1ΔTMs-EFF-1 TM mutant by overlap PCR which contains one FLAG tag

Supplementary References

1. Lu, X. *et al.* Double Lock of a Human Neutralizing and Protective Monoclonal Antibody Targeting the Yellow Fever Virus Envelope. *Cell Rep.* **26**, 438–446.e5 (2019).
2. Voss, J. E. *et al.* Glycoprotein organization of Chikungunya virus particles revealed by X-ray crystallography. *Nature* **468**, 709–712 (2010).
3. Modis, Y., Ogata, S., Clements, D. & Harrison, S. C. A ligand-binding pocket in the dengue virus envelope glycoprotein. *Proc. Natl. Acad. Sci. U. S. A.* **100**, 6986–6991 (2003).
4. Gibbons, D. L. *et al.* Conformational change and protein-protein interactions of the fusion protein of Semliki Forest virus. *Nature* **427**, 320–325 (2004).
5. Rey, F. A., Heinz, F. X., Mandl, C., Kunz, C. & Harrison, S. C. The envelope glycoprotein from tick-borne encephalitis virus at 2 Å resolution. *Nature* **375**, 291–298 (1995).
6. Fedry, J. *et al.* Evolutionary diversification of the HAP2 membrane insertion motifs to drive gamete fusion across eukaryotes. *PLoS Biol.* **16**, e2006357 (2018).
7. DuBois, R. M. *et al.* Functional and evolutionary insight from the crystal structure of rubella virus protein E1. *Nature* **493**, 552–556 (2013).
8. Fédry, J. *et al.* The Ancient Gamete Fusogen HAP2 Is a Eukaryotic Class II Fusion Protein. *Cell* **168**, 904–915.e10 (2017).
9. Guardado-Calvo, P. *et al.* Mechanistic Insight into Bunyavirus-Induced Membrane Fusion from Structure-Function Analyses of the Hantavirus Envelope Glycoprotein Gc. *PLoS Pathog.* **12**, e1005813 (2016).
10. Dessau, M. & Modis, Y. Crystal structure of glycoprotein C from Rift Valley fever virus. *Proc. Natl. Acad. Sci. U. S. A.* **110**, 1696–1701 (2013).
11. Kielian, M. Mechanisms of Virus Membrane Fusion Proteins. *Annu. Rev. Virol.* **1**, 171–189 (2014).
12. Baquero, E., Fedry, J., Legrand, P., Krey, T. & Rey, F. A. Species-Specific Functional Regions of the Green Alga Gamete Fusion Protein HAP2 Revealed by Structural Studies. *Structure* **27**, 113–124.e4 (2019).
13. Söding, J. Protein homology detection by HMM–HMM comparison. *Bioinformatics* **21**, 951–960 (2004).

14. Avinoam, O. *et al.* Conserved eukaryotic fusogens can fuse viral envelopes to cells. *Science* **332**, 589–592 (2011).
15. Williams, R. M. *et al.* Genome and epigenome engineering CRISPR toolkit for in vivo modulation of cis-regulatory interactions and gene expression in the chicken embryo. *Development* **145**, dev160333 (2018).
16. Matsuda, T. & Cepko, C. L. Electroporation and RNA interference in the rodent retina in vivo and in vitro. *Proc. Natl. Acad. Sci. U. S. A.* **101**, 16–22 (2004).
17. Dunsing, V. *et al.* Optimal fluorescent protein tags for quantifying protein oligomerization in living cells. *Sci. Rep.* **8**, 1–12 (2018).
