# Supplementary material for: Discovery of archaeal fusexins homologous to eukaryotic HAP2/GCS1 gamete fusion proteins
Source: Nat Commun. 2022 Jul 6;13:3880. doi: 10.1038/s41467-022-31564-1 (PMC9259645; doi:10.1038/s41467-022-31564-1)
Supplement: Supplementary file 2 — Description of Additional Supplementary Files [file 41467_2022_31564_MOESM2_ESM.pdf]

Title: Supplementary Movie 1
Description: Time-lapse experiment using spinning disk confocal microscopy reveals merging of two cells expressing myr-mCherry and Fsx1. Time in hours:minutes. Merge of the red and DIC channels is shown.

Title: Supplementary Movie 2
Description: Z-series of the binucleated BHK cell from Fig. 4h. Labeled nuclei (blue) and myr-mCherry (white). Each optical section obtained with spinning disc confocal microscopy is 1 µm apart.

Title: Supplementary Data 1
Description: This table contains a complete list of the Fsx1-encoding genes identified in this study with ID, amino acid sequence and associated environmental and genomic data.

Title: Supplementary Data 2
Description: This table contains a complete list of PCGs' IME ORFs annotated with identified Pfam, TIGR and arCOGs domains.

Title: Supplementary Data 3
Description: This table contains a complete list of the 11 IME ORFs (as shown in Fig. 8) annotated with identified arCOGs domains.

Title: Supplementary Data 4.
Description: This file contains the sequences of the ten fsx1s genes synthesized for expression tests and fusogenic activities.

Title: Supplementary Data 5.
Description: This file contains the Fsx1 HAP2 tree in newick format with branch support values.
