# Supplementary material for: Discovery of archaeal fusexins homologous to eukaryotic HAP2/GCS1 gamete fusion proteins
Source: Nat Commun. 2022 Jul 6;13:3880. doi: 10.1038/s41467-022-31564-1 (PMC9259645; doi:10.1038/s41467-022-31564-1)
Supplement: Supplementary file 3 — Reporting Summary [file 41467_2022_31564_MOESM3_ESM.pdf]

nature portfolio

Corresponding author(s): Martin Graña, Héctor Romero, Pablo S. Aguilar, Luca Jovine, Benjamin Podbilewicz

Last updated by author(s): Jun 21, 2022

Reporting Summary

Nature Portfolio wishes to improve the reproducibility of the work that we publish. This form provides structure for consistency and transparency in reporting. For further information on Nature Portfolio policies, see our Editorial Policies and the Editorial Policy Checklist.

Statistics

For all statistical analyses, confirm that the following items are present in the figure legend, table legend, main text, or Methods section.

n/a	Confirmed	
☐	☒	The exact sample size (n) for each experimental group/condition, given as a discrete number and unit of measurement
☐	☒	A statement on whether measurements were taken from distinct samples or whether the same sample was measured repeatedly
☐	☒	The statistical test(s) used AND whether they are one- or two-sided<br>*Only common tests should be described solely by name; describe more complex techniques in the Methods section.*
☒	☐	A description of all covariates tested
☐	☒	A description of any assumptions or corrections, such as tests of normality and adjustment for multiple comparisons
☐	☒	A full description of the statistical parameters including central tendency (e.g. means) or other basic estimates (e.g. regression coefficient) AND variation (e.g. standard deviation) or associated estimates of uncertainty (e.g. confidence intervals)
☐	☒	For null hypothesis testing, the test statistic (e.g. F, t, r) with confidence intervals, effect sizes, degrees of freedom and P value noted<br>*Give P values as exact values whenever suitable.*
☒	☐	For Bayesian analysis, information on the choice of priors and Markov chain Monte Carlo settings
☒	☐	For hierarchical and complex designs, identification of the appropriate level for tests and full reporting of outcomes
☒	☐	Estimates of effect sizes (e.g. Cohen's d, Pearson's r), indicating how they were calculated

Our web collection on statistics for biologists contains articles on many of the points above.

Software and code

Policy information about availability of computer code

Data collection	MetaMorph(v7.8.1.0), Micro-Manager(v1.4.22), MicroMounts(MiTeGen) , MXCuBE3, ZEN.
Data analysis	Adobe Illustrator 2021 , Adobe Photoshop CS6, AlphaFold2(v2.1.1), AlphaFold-Multimer(v2.1.1),ANODE(v2013/1), APBS(v1.5), ASTRA(v7.1.3), BioRender, BLASTP,ChimeraX(v1.2.4), ClustalO(v1.2.2) , ConSurf, Coot(v0.8.9.3-0.9.6), CRYSOL(ATSAS v3.0.3), Dali(v5), DAMMIF(ATSAS v3.0.2) , DSSP(v4.0-67), EMBOSS(v6.5.7), ETE Toolkit, FastME(v2.0), FATCAT(v2.0), GraphPad Prism 9, HHblits(v3.3.0), HH-suite(v3.3.0), HMMER (v3.3.2), I-TASSER(v5.1), ImageJ(v1.53c), IQ-TREE2(v1.6.12) , ISOLDE(v1.1.0), JCVI, kClust, MAFFT (v7.310) , MCscan(v0.8), MetaMorph (v7.8.1.0), ModelFinder, MODELLER(v10.2), MolProbity(v4.5.1), MOLREP(v11.7.03), PDB2PQR(v2.1.2), PDBeFold, PDBsum, Pfam(v33), Phaser (v2.8.3), PHENIX AutoBuild(v1.19.2) , PIC, PISA(v1.52), POLARRFN(v7.1.010), POSA, PRIMUS(ATSAS v3.0.3), PyMOL(v2.4.2), Python, R, trim-AI, TMalign(v20210224) , TMHMM(v2.0), TOPCONS(v2.0), XDS(version Jan 31, 2020 BUILT=20200417) , YASARA2(v.21.7.1). All relevant codes, notebooks and datasets necessary for: HHblits and Hmmer searches and comparisons (Fig. 1a, Supplementary Fig. 1a); Kmer spectra analyses (Fig. 7); IMEs clustering, content and synteny analyses (Fig. 8, Supplementary Figs. 8, 9 and Supplementary Tables 3, 4), protein sequence and structure-based comparisons (Supplementary Fig. 9) are available on GitHub (https://github.com/DessimozLab/Archaeal-Fusexins) and Zenodo (https://doi.org/10.5281/zenodo.6669786).

For manuscripts utilizing custom algorithms or software that are central to the research but not yet described in published literature, software must be made available to editors and reviewers. We strongly encourage code deposition in a community repository (e.g. GitHub). See the Nature Portfolio guidelines for submitting code & software for further information.

Data

Policy information about availability of data

All manuscripts must include a data availability statement. This statement should provide the following information, where applicable:

- Accession codes, unique identifiers, or web links for publicly available datasets
- A description of any restrictions on data availability
- For clinical datasets or third party data, please ensure that the statement adheres to our policy

All relevant data are included in the manuscript and as Supplementary Information files (see Suppl. Inf. Guide). Crystallographic structure factors and atomic coordinates have been deposited in the Protein Data Bank under accession code 7P4L.

Field-specific reporting

Please select the one below that is the best fit for your research. If you are not sure, read the appropriate sections before making your selection.

☒ Life sciences ☐ Behavioural & social sciences ☐ Ecological, evolutionary & environmental sciences

For a reference copy of the document with all sections, see nature.com/documents/nr-reporting-summary-flat.pdf

Life sciences study design

All studies must disclose on these points even when the disclosure is negative.

Sample size	We did not perform calculations to determine sample sizes; however, when we added more data the conclusions of the experiments did not change.
Data exclusions	No data were excluded from the analyses.
Replication	Functional experiments were independently repeated on different days and using independently transfected cells. Interobserver error was estimated for counting of multinucleated cells, cells in contact, and content-mixing experiments: the differences in percentages of multinucleation and content mixing obtained by two observers was <10%.
Randomization	In our study we did not allocate experimental groups; thus, randomization was not necessary.
Blinding	Counting of content mixing and multinucleation was made blind for the experiments included in Figs. 4f and 5b. Other experiments in this study were analyzed using the same methodologies and all data were included in the results reported.

Reporting for specific materials, systems and methods

We require information from authors about some types of materials, experimental systems and methods used in many studies. Here, indicate whether each material, system or method listed is relevant to your study. If you are not sure if a list item applies to your research, read the appropriate section before selecting a response.

Materials & experimental systems

n/a	Involved in the study	
☐	☒	Antibodies
☐	☒	Eukaryotic cell lines
☒	☐	Palaeontology and archaeology
☒	☐	Animals and other organisms
☒	☐	Human research participants
☒	☐	Clinical data
☒	☐	Dual use research of concern

Methods

n/a	Involved in the study	
☒	☐	ChIP-seq
☒	☐	Flow cytometry
☒	☐	MRI-based neuroimaging

Antibodies

Antibodies used	Anti-FLAG M2, catalog number F3165, lot number 065K6236 (Sigma); Anti-V5 mouse monoclonal, catalog number R960-25, lot number 2001339 (Invitrogen/Life Science); Donkey anti-mouse coupled to Alexa Fluor 488, catalog number A21202, lot number 1423052 (Invitrogen); Mouse Anti-Actin monoclonal C4, catalog number 691001, lot number Q1642 (MP Biomedicals); HRP-conjugated goat anti-mouse, catalog number 115-035-003, lot number n/a (Jackson ImmunoResearch Laboratories, Inc.) .
Validation	Negative controls for immunofluorescence and western blots were un-transfected cells, cells transfected with vector alone or cells

transfected with a plasmid that did not contain the V5 or FLAG tags. Secondary antibodies were used on cells that were not incubated with first antibodies to determine the background. Positive controls were tagged proteins that have been used previously in the lab (CeEFF-1-V5, CeAFF-1-FLAG, AtHAP2-V5). Additional information can be found here: https://www.sigmaaldrich.com/IL/en/product/sigma/f3165; https://www.thermofisher.com/antibody/product/V5-Tag-Antibody-Monoclonal/R960-25; https://www.mpbio.com/au/08691001-mouse-anti-actin-monoclonal-clone-c4-cf; https://www.jacksonimmuno.com/catalog/products/115-035-003.

Eukaryotic cell lines

Policy information about cell lines

Cell line source(s)	HEK293T: ATCC catalog number CRL-3216 (PMID 3031469); BHK-21: kindly obtained from Judith White (University of Virginia).
Authentication	None of the cell lines used were authenticated.
Mycoplasma contamination	Cells were routinely tested for mycoplasma and no contamination was detected; in particular, we confirmed that HEK293T were mycoplasma-free by using a PCR Mycoplasma Test Kit II (Applichem cat. no. A8994).
Commonly misidentified lines (See ICLAC register)	Cell lines HEK293T (CVCL_0063) and BHK-21 are not listed in version 8 of the Database of Cross-Contaminated or Misidentified Cell Lines.
