# Supplementary material for: Discovery of archaeal fusexins homologous to eukaryotic HAP2/GCS1 gamete fusion proteins
Source: Nat Commun. 2022 Jul 6;13:3880. doi: 10.1038/s41467-022-31564-1 (PMC9259645; doi:10.1038/s41467-022-31564-1)
Supplement: Supplementary file 8 — Supplementary Data 4 [file 41467_2022_31564_MOESM8_ESM.zip › Supplementary Data 4.docx]

Synthesized plasmid: pBPT01

NZ_JNCS01000001.1:501753-503768 Natrinema altunense strain AJ2 N_altunense_AJ2_contig_1, whole genome shotgun sequence 5’ to 3’

>N.altFusexin1-V56HIS (pBPT01)EDITED

**pBPT01 Edited : Addition of restriction Ez. Sites**

**5’ KpnI, 3’ SacI, SphI, SpeI**

ggtaccATGCGGGCGGTGTCTGATTTCCTGAAGAATAAGTGGGTAGCAGTACCTGCTGTCGCTCTTCTAATCCTCAGTCTCGGTTTTCTGGCGCAGAACTATATTACAGGGTCGTTTGTCAGCGGTGATCAGATAGAGTATACCAGTAATTCGGAGTTCTTCGATGGCGAGGTCTTCGTCATCAACTATATCGGTGACAGGACGACCGACAAGATACACGCGGAGATCCCGGCATCTGAGTTAGCCAGTGCTGCTGACGGAGAAGTCACGAAGGAATTGACGATCGACATCACCTCGCAAGAGACTTACGCCCGGTATAGTACGACATCGACGAGCCTGAGCAGGATTTACGCTTTCAAGGCGATCAATTCGGAGGTGTTTACTGATACGTCTTCTTCGGAGGTTGAGTCTCGGAAGCAGTCTTGGGCGGAGCAGAACTGCTACGATATAGACGGTGATTCGGAGATTGAGCAGGGTGATGATTACACTCAGCGGTTCTGGGTGGATGGTGCGATTGCGGATTACTACAATGCTCAGGTGTACTGTGTTCGGGAAAATGGATACTATGGGAATATCGCGGAGTTGAGTTCGCCTGACAAGGAGTTTGAGGCAGAGGTCGAGGTGAAGGCGGATGGTGAGACACCTCAGTCGACCACTCTAAGTAACTCAGATCTTGGTCAGGGAAGATGCGACAACATCGGGGAGTATGTCAGGGCTTGCTGGACCGGTTTGAATCCGACCGGTGAAGGAGTTCCTGAGCCGTATAATGTCTACGCAATTCACAGCAACACGTTCTCCCCGGAGTGGAGAGTAATCTCAGCGGAGCGGTACTCGTCGTACAACAGCTATGTCGAGAACAATCTGTACAGTCAGATCGAGGCTTGGAAGGAAGGATCTCTGAGTCAGGAAGAGGTTGTCTCGACTGCGAATGATCAGGCGGCTCAAGCAGCTCAAAGAGTTTCCTCTGGCAGCTTTACTGATTACCAGGTGGAGGACTCTTCCTACGAGAATGGGCAGATCCGGTTGAATCCGAGTTATGATATTGCCTGGCCGGAGTTCACGTTCTACGTGGACGGTGCGGACTATATCTCGGTTGAGAAGCCGGTAGGTGAGCCGAGGATAGTGAATGTTGAAGGAGATGAGTTTGGTGAGAAGCAGTCTGGCACTGTTACAGCCGAAGTGGAGAACACGGCGAGCTACGAAGGAAGTTTCTCTGCCAGGGTTTCCAAGTGTAGCGATAGCTTCGGTTATGATTCGTTGCAGGAGACAAAGACTGTGGGTCCCGGTGAGACTGTTTCCTTCGATTTCCGGGTGAGCTTCACTTCGGATAGTTTCGATCAGAAAACGGTCTCTGGTAGCTGTGAGATCGTGGTTTCTGATACCGGTGATTCCTCAAATAGTGATACCGCGGTAGTGGATGTTGAGGCTACTCAGAGTAATGAGTGTACTCCGAACGAGGAGTTCGTCAGGACGATCAACTCAACTCACTCCAGGATCCTCGAGTGTAGCAGTGATGGTTTGACCACGACCGAAGTAGATGTCTGTGGAGACGGTGAGGAAGCAGCCTTCAGAGATAGCTCTTGGACCTGTGTATCTGATGGAACTCTTCCTGGAGGCGGTGACGGATCCGGAGGTTCCGGTGGCGGAGGAGATACGGATGACTGTGTAGTGTTTGCCGTGGGAGATCTGGAGATTGAGGATCCGTTCTGTGCGGATGGTCCGTTGGAAATGCTGTCGAAGATGTTCCACTTGGTGGCAGGGACAGCGGTAGCGTTCTTCACGGGAAGCCTGGGTTACAGGGCTGGTCGTTGGGTTGACGGCGAGTACCAGATTAAGGGAGGTTTCGACCCGTTAAAATCTCGAAGCGTATCCCGCGCTAAGAGAGGAAGATTCCTGATAGGACTGATCGCTGAATTGGTTTCGTTCCTGCTCGGGTTCTATGTGATCCTCTTGGTGCCGATATGGGCACAGTTGATGGTGATCCTGGGATACGTCCTGTTCAAGTATTATACGCCGTTTCTAGAGGGCCCGCGGTTCGAAGGTAAGCCTATCCCTAACCCTCTCCTCGGTCTCGATTCTACGCGTACCGGTCATCATCACCATCACCATTGAgagctcgcatgcactagt

>N.altFusexin1-V56HIS (pBPT01)EDITED

From 1 to 2130.

ggtaccATGCGGGCGGTGTCTGATTTCCTGAAGAATAAGTGGGTAGCAGTACCTGCTGTCGCTCTTCTAATCCTCAGTCTCGGTTTTCTG 90

M R A V S D F L K N K W V A V P A V A L L I L S L G F L 30

GCGCAGAACTATATTACAGGGTCGTTTGTCAGCGGTGATCAGATAGAGTATACCAGTAATTCGGAGTTCTTCGATGGCGAGGTCTTCGTC 180

A Q N Y I T G S F V S G D Q I E Y T S N S E F F D G E V F V 60

ATCAACTATATCGGTGACAGGACGACCGACAAGATACACGCGGAGATCCCGGCATCTGAGTTAGCCAGTGCTGCTGACGGAGAAGTCACG 270

I N Y I G D R T T D K I H A E I P A S E L A S A A D G E V T 90

AAGGAATTGACGATCGACATCACCTCGCAAGAGACTTACGCCCGGTATAGTACGACATCGACGAGCCTGAGCAGGATTTACGCTTTCAAG 360

K E L T I D I T S Q E T Y A R Y S T T S T S L S R I Y A F K 120

GCGATCAATTCGGAGGTGTTTACTGATACGTCTTCTTCGGAGGTTGAGTCTCGGAAGCAGTCTTGGGCGGAGCAGAACTGCTACGATATA 450

A I N S E V F T D T S S S E V E S R K Q S W A E Q N C Y D I 150

GACGGTGATTCGGAGATTGAGCAGGGTGATGATTACACTCAGCGGTTCTGGGTGGATGGTGCGATTGCGGATTACTACAATGCTCAGGTG 540

D G D S E I E Q G D D Y T Q R F W V D G A I A D Y Y N A Q V 180

TACTGTGTTCGGGAAAATGGATACTATGGGAATATCGCGGAGTTGAGTTCGCCTGACAAGGAGTTTGAGGCAGAGGTCGAGGTGAAGGCG 630

Y C V R E N G Y Y G N I A E L S S P D K E F E A E V E V K A 210

GATGGTGAGACACCTCAGTCGACCACTCTAAGTAACTCAGATCTTGGTCAGGGAAGATGCGACAACATCGGGGAGTATGTCAGGGCTTGC 720

D G E T P Q S T T L S N S D L G Q G R C D N I G E Y V R A C 240

TGGACCGGTTTGAATCCGACCGGTGAAGGAGTTCCTGAGCCGTATAATGTCTACGCAATTCACAGCAACACGTTCTCCCCGGAGTGGAGA 810

W T G L N P T G E G V P E P Y N V Y A I H S N T F S P E W R 270

GTAATCTCAGCGGAGCGGTACTCGTCGTACAACAGCTATGTCGAGAACAATCTGTACAGTCAGATCGAGGCTTGGAAGGAAGGATCTCTG 900

V I S A E R Y S S Y N S Y V E N N L Y S Q I E A W K E G S L 300

AGTCAGGAAGAGGTTGTCTCGACTGCGAATGATCAGGCGGCTCAAGCAGCTCAAAGAGTTTCCTCTGGCAGCTTTACTGATTACCAGGTG 990

S Q E E V V S T A N D Q A A Q A A Q R V S S G S F T D Y Q V 330

GAGGACTCTTCCTACGAGAATGGGCAGATCCGGTTGAATCCGAGTTATGATATTGCCTGGCCGGAGTTCACGTTCTACGTGGACGGTGCG 1080

E D S S Y E N G Q I R L N P S Y D I A W P E F T F Y V D G A 360

GACTATATCTCGGTTGAGAAGCCGGTAGGTGAGCCGAGGATAGTGAATGTTGAAGGAGATGAGTTTGGTGAGAAGCAGTCTGGCACTGTT 1170

D Y I S V E K P V G E P R I V N V E G D E F G E K Q S G T V 390

ACAGCCGAAGTGGAGAACACGGCGAGCTACGAAGGAAGTTTCTCTGCCAGGGTTTCCAAGTGTAGCGATAGCTTCGGTTATGATTCGTTG 1260

T A E V E N T A S Y E G S F S A R V S K C S D S F G Y D S L 420

CAGGAGACAAAGACTGTGGGTCCCGGTGAGACTGTTTCCTTCGATTTCCGGGTGAGCTTCACTTCGGATAGTTTCGATCAGAAAACGGTC 1350

Q E T K T V G P G E T V S F D F R V S F T S D S F D Q K T V 450

TCTGGTAGCTGTGAGATCGTGGTTTCTGATACCGGTGATTCCTCAAATAGTGATACCGCGGTAGTGGATGTTGAGGCTACTCAGAGTAAT 1440

S G S C E I V V S D T G D S S N S D T A V V D V E A T Q S N 480

GAGTGTACTCCGAACGAGGAGTTCGTCAGGACGATCAACTCAACTCACTCCAGGATCCTCGAGTGTAGCAGTGATGGTTTGACCACGACC 1530

E C T P N E E F V R T I N S T H S R I L E C S S D G L T T T 510

GAAGTAGATGTCTGTGGAGACGGTGAGGAAGCAGCCTTCAGAGATAGCTCTTGGACCTGTGTATCTGATGGAACTCTTCCTGGAGGCGGT 1620

E V D V C G D G E E A A F R D S S W T C V S D G T L P G G G 540

GACGGATCCGGAGGTTCCGGTGGCGGAGGAGATACGGATGACTGTGTAGTGTTTGCCGTGGGAGATCTGGAGATTGAGGATCCGTTCTGT 1710

D G S G G S G G G G D T D D C V V F A V G D L E I E D P F C 570

GCGGATGGTCCGTTGGAAATGCTGTCGAAGATGTTCCACTTGGTGGCAGGGACAGCGGTAGCGTTCTTCACGGGAAGCCTGGGTTACAGG 1800

A D G P L E M L S K M F H L V A G T A V A F F T G S L G Y R 600

GCTGGTCGTTGGGTTGACGGCGAGTACCAGATTAAGGGAGGTTTCGACCCGTTAAAATCTCGAAGCGTATCCCGCGCTAAGAGAGGAAGA 1890

A G R W V D G E Y Q I K G G F D P L K S R S V S R A K R G R 630

TTCCTGATAGGACTGATCGCTGAATTGGTTTCGTTCCTGCTCGGGTTCTATGTGATCCTCTTGGTGCCGATATGGGCACAGTTGATGGTG 1980

F L I G L I A E L V S F L L G F Y V I L L V P I W A Q L M V 660

ATCCTGGGATACGTCCTGTTCAAGTATTATACGCCGTTTCTAGAGGGCCCGCGGTTCGAAGGTAAGCCTATCCCTAACCCTCTCCTCGGT 2070

I L G Y V L F K Y Y T P F L E G P R F E G K P I P N P L L G 690

CTCGATTCTACGCGTACCGGTCATCATCACCATCACCATTGAgagctcgcatgcactagt 2130

L D S T R T G H H H H H H * 710

Synthesized plasmid: pBPT02

>HaloferaxQ22Fusexin1-V5-6HIS pBPT02-edited

**Addition of restriction Ez. Sites**

**5’ KpnI, 3’ SacI, SphI, SpeI**

KpnI edited GGTACC to GGTATC both encode for Tyr

ggtaccATGAAAAACGGGTTGAAGGCCTCGGTAGTAGCACTGTTTTTCCTATTAGCCGCTTCTTCTGCATCAGCAGCGACGAACTCTGTTGACACAGTCACCTACGAGAGCAACAGTGATTTCTTCGACGGACAGGTTCTCCAAGTAGGTTATACCTCTAATTTTGCTACGGACAAGATCAACGTATATCTCTCTGAAAGCAAGATCGAGAGTGAGACTGGAGCGGTAGCGGATGAACCGATTACGTTCGATGTTTCTCACCAGAATACGTACGCCAAGTACCCAACTCAGGATACAGGTTTAGAGTCGATAACTGGTTGGGACGCAGTCAAGAAGACTGTCAGCTCTAAGCAGGAGCTGTGGGACTGGACTACCACGAACTGTGTCGATACGAACGATGCTGGCACGTTCACAGTCGATGGTTACGGGACGACTGATGTAGATGCTCGAGCGAACAGATGGTTCGATACCTGGACAGGGCAGTACAAGTATACAATCTATTGCTGGCAGAGAAATGATTACTACGGTGCTGTAGCTGACATCGGTTCTCCTGACGAAGTCTTCCGGACAGAGTGGAGGCTTCAGGCCGGAGATAAGAATCCTCAGACAGCTGTGATCACTAACGGCGACGGCGGAACAGGAGTGACCTCTAACCTCGGCAGATATGCCAAGGTAAGCTGGGAGGGAAGTCTTTCAACCGGTGAGAATCCTCCGCTCGTCGATGATGAATATGCTCTCCACAGTAATGACTACGAAGGCGGTTGGAGAGTCATAAGCGAATCACGTTACTCCTCGTACCAGAGTTTCGTCCAGAACAATGCTGACGACCTTCTTGGAGAATGGGGTGCAGGCCTAACCACTGAGAGCCACATCGAGAGCGAGATGAACGGAAAAGCGGAAGAAGCTGCTTCGGAGTTTACGGAGTCTCCGCTTTCTGACTCCGAGATCTTGGACTCGAGCTTCCAGAATGGTGCGTTCAAACTGGATATGGAGCGTAGCCTGATGTACCCCGAGTTCAGTGTCTACGTTGATGCGGGTGAGAACGGTTATGCCACGATATCTAAGCCTGTAGGTAAGCCAGAGATCGTCTCTACAAGCGGTGCAGAGTTCGGTGAATTGGGTCAGGGAACGATATCAGTGGAAGCAGAAAATGTCGGTGATAGCGAAGGCAGTTTCTCGGCTCGTACCGATTCCTGCGGCGAGTACTTCAGCGGTAACAGCTTGCAGGATACTCAAAGGGTTTCTCCTGGCGGTACTGCATCGTTTGATTTCCGGGTGACGTTCACTTCGACGAGTATGACGCAGAGTGAGTTCTCTGACCAGTGTGAAGTTGTTGTTGAGGATACTGGGAGTGGGAACGAGGTCTCTGCCAGTGTCTCTGTTACTGCTACTCAGGAAGATGAGTGTACAGAGGGAGATGAGACGAAGAAGGAGAAACAGGTAAACGGTGAAACGGTCGACGTCATCATGTCCTGTACTAATGGCCTGAAACTGGAGGAGGACGAAGTCTGCAGTGCTGATGAGGAAGCTCGATACATTGACGACGATGTCCAGTATGAATGCCGTGACAAGGACTCGCCTCCCGGTGGAGGCGGCGGTGATCCGTGGACTATTCCAGGCCTCGGATGGCAACTAGATACTCCATTTGGCGGTATGGAGAAGGCCTTATCCGGTAACGCTGGTGCATTGACCTGGGCTCAATTGTTGCTGTCGTTCATCGGATTCCTCGCTGGATTCGCCTTAGTAGGGGTTAAGCTCGGTAAGATGGTTGACGGCCTTGCCACGGAGTTCATCCCATTGAGTGATGCCGTCGTGAGACTGGGTATAGGACTTGTAGGAGGCGGGATGGCGTTCATGGCTGTGTATCAGTTAGTGACGAATCCGTTAGGGTTCTTGCTGACTGTTGTCGGCCTGCTTCTAACAGGGTAtCTGTATTTGAAGGGTACTACTCCGGACATTAACTTGCTAGAGGGCCCGCGGTTCGAAGGTAAGCCTATCCCTAACCCTCTCCTCGGTCTCGATTCTACGCGTACCGGTCATCATCACCATCACCATTGAgagctcgcatgcactagt

>HaloferaxQ22Fusexin1-V5-6HIS pBPT02-edited

From 1 to 2076.

ggtaccATGAAAAACGGGTTGAAGGCCTCGGTAGTAGCACTGTTTTTCCTATTAGCCGCTTCTTCTGCATCAGCAGCGACGAACTCTGTT 90

M K N G L K A S V V A L F F L L A A S S A S A A T N S V 30

GACACAGTCACCTACGAGAGCAACAGTGATTTCTTCGACGGACAGGTTCTCCAAGTAGGTTATACCTCTAATTTTGCTACGGACAAGATC 180

D T V T Y E S N S D F F D G Q V L Q V G Y T S N F A T D K I 60

AACGTATATCTCTCTGAAAGCAAGATCGAGAGTGAGACTGGAGCGGTAGCGGATGAACCGATTACGTTCGATGTTTCTCACCAGAATACG 270

N V Y L S E S K I E S E T G A V A D E P I T F D V S H Q N T 90

TACGCCAAGTACCCAACTCAGGATACAGGTTTAGAGTCGATAACTGGTTGGGACGCAGTCAAGAAGACTGTCAGCTCTAAGCAGGAGCTG 360

Y A K Y P T Q D T G L E S I T G W D A V K K T V S S K Q E L 120

TGGGACTGGACTACCACGAACTGTGTCGATACGAACGATGCTGGCACGTTCACAGTCGATGGTTACGGGACGACTGATGTAGATGCTCGA 450

W D W T T T N C V D T N D A G T F T V D G Y G T T D V D A R 150

GCGAACAGATGGTTCGATACCTGGACAGGGCAGTACAAGTATACAATCTATTGCTGGCAGAGAAATGATTACTACGGTGCTGTAGCTGAC 540

A N R W F D T W T G Q Y K Y T I Y C W Q R N D Y Y G A V A D 180

ATCGGTTCTCCTGACGAAGTCTTCCGGACAGAGTGGAGGCTTCAGGCCGGAGATAAGAATCCTCAGACAGCTGTGATCACTAACGGCGAC 630

I G S P D E V F R T E W R L Q A G D K N P Q T A V I T N G D 210

GGCGGAACAGGAGTGACCTCTAACCTCGGCAGATATGCCAAGGTAAGCTGGGAGGGAAGTCTTTCAACCGGTGAGAATCCTCCGCTCGTC 720

G G T G V T S N L G R Y A K V S W E G S L S T G E N P P L V 240

GATGATGAATATGCTCTCCACAGTAATGACTACGAAGGCGGTTGGAGAGTCATAAGCGAATCACGTTACTCCTCGTACCAGAGTTTCGTC 810

D D E Y A L H S N D Y E G G W R V I S E S R Y S S Y Q S F V 270

CAGAACAATGCTGACGACCTTCTTGGAGAATGGGGTGCAGGCCTAACCACTGAGAGCCACATCGAGAGCGAGATGAACGGAAAAGCGGAA 900

Q N N A D D L L G E W G A G L T T E S H I E S E M N G K A E 300

GAAGCTGCTTCGGAGTTTACGGAGTCTCCGCTTTCTGACTCCGAGATCTTGGACTCGAGCTTCCAGAATGGTGCGTTCAAACTGGATATG 990

E A A S E F T E S P L S D S E I L D S S F Q N G A F K L D M 330

GAGCGTAGCCTGATGTACCCCGAGTTCAGTGTCTACGTTGATGCGGGTGAGAACGGTTATGCCACGATATCTAAGCCTGTAGGTAAGCCA 1080

E R S L M Y P E F S V Y V D A G E N G Y A T I S K P V G K P 360

GAGATCGTCTCTACAAGCGGTGCAGAGTTCGGTGAATTGGGTCAGGGAACGATATCAGTGGAAGCAGAAAATGTCGGTGATAGCGAAGGC 1170

E I V S T S G A E F G E L G Q G T I S V E A E N V G D S E G 390

AGTTTCTCGGCTCGTACCGATTCCTGCGGCGAGTACTTCAGCGGTAACAGCTTGCAGGATACTCAAAGGGTTTCTCCTGGCGGTACTGCA 1260

S F S A R T D S C G E Y F S G N S L Q D T Q R V S P G G T A 420

TCGTTTGATTTCCGGGTGACGTTCACTTCGACGAGTATGACGCAGAGTGAGTTCTCTGACCAGTGTGAAGTTGTTGTTGAGGATACTGGG 1350

S F D F R V T F T S T S M T Q S E F S D Q C E V V V E D T G 450

AGTGGGAACGAGGTCTCTGCCAGTGTCTCTGTTACTGCTACTCAGGAAGATGAGTGTACAGAGGGAGATGAGACGAAGAAGGAGAAACAG 1440

S G N E V S A S V S V T A T Q E D E C T E G D E T K K E K Q 480

GTAAACGGTGAAACGGTCGACGTCATCATGTCCTGTACTAATGGCCTGAAACTGGAGGAGGACGAAGTCTGCAGTGCTGATGAGGAAGCT 1530

V N G E T V D V I M S C T N G L K L E E D E V C S A D E E A 510

CGATACATTGACGACGATGTCCAGTATGAATGCCGTGACAAGGACTCGCCTCCCGGTGGAGGCGGCGGTGATCCGTGGACTATTCCAGGC 1620

R Y I D D D V Q Y E C R D K D S P P G G G G G D P W T I P G 540

CTCGGATGGCAACTAGATACTCCATTTGGCGGTATGGAGAAGGCCTTATCCGGTAACGCTGGTGCATTGACCTGGGCTCAATTGTTGCTG 1710

L G W Q L D T P F G G M E K A L S G N A G A L T W A Q L L L 570

TCGTTCATCGGATTCCTCGCTGGATTCGCCTTAGTAGGGGTTAAGCTCGGTAAGATGGTTGACGGCCTTGCCACGGAGTTCATCCCATTG 1800

S F I G F L A G F A L V G V K L G K M V D G L A T E F I P L 600

AGTGATGCCGTCGTGAGACTGGGTATAGGACTTGTAGGAGGCGGGATGGCGTTCATGGCTGTGTATCAGTTAGTGACGAATCCGTTAGGG 1890

S D A V V R L G I G L V G G G M A F M A V Y Q L V T N P L G 630

TTCTTGCTGACTGTTGTCGGCCTGCTTCTAACAGGGTAtCTGTATTTGAAGGGTACTACTCCGGACATTAACTTGCTAGAGGGCCCGCGG 1980

F L L T V V G L L L T G Y L Y L K G T T P D I N L L E G P R 660

TTCGAAGGTAAGCCTATCCCTAACCCTCTCCTCGGTCTCGATTCTACGCGTACCGGTCATCATCACCATCACCATTGAgagctcgcatgc 2070

F E G K P I P N P L L G L D S T R T G H H H H H H * 690

actagt 2076

692

Synthesized plasmid: pBPT03

NZ_KE386573.1:2594348-2596255 Haloplanus natans DSM 17983 HALNADRAFT_scaffold1.1, whole genome shotgun sequence

>H.natansFusexin1-V5-6His (pBPT03)EDITED (SacI-KpnI)

**Addition of restriction Ez. Sites**

**5’ KpnI, 3’ SacI, SphI, SpeI**

KpnI: edited GGTACC to GGTACT both encoding for Thr

SacI: GAGCTC to GAGCTT both encoding for Leu

ggtaccATGTTCACTGCATTTGCGGCAGCAGATACTACTTCGGTGGATACTGTCTCGTATAAGAGCAACAGCGAGTTCTTCGACGGAGAAGTTCTTCAGGCTGGTTATGTCTCGAACTTTGCGACTGACAAGATTAACGTCCATCTCGGTGCCAGTGAGATTGAGAGCAAGACCGGAGCTACTGCGGAAGAAGACTTGACTTTGAGTGTTTCTCACCAGAACACCTACGCTCGCTACCCGCTCCAGGAGACCGGTTTGAAGAAGATCTACGGCTGGGAGGGGATGAAGAAGACATTCGATACGAAAGACCAGCTATGGAACTGGGTTACCTCAAACTGTGCTGACTTCACTGAGGGCGGAGTAGCGATCGGAGATAGAACCAGTAAGGTCGAGGCCGCTGCCAAGAGCTGGTACGATTACTGGACAGGAAGTTACAATTACCAGGCCTTCTGCCTGCGGAAGAACGGGTACTACGGAGATGTAGCTGATATCGGCTCCCCGGATGAAATCTTCAGAACCGAGTGGAGGCTGCAAGCAGGCGATAAGAATCCGCAGACTGCTATAATAACAAATGGGGATGGTGGATCAGGTGTTGTCTCCAATCTTGGCCGGTATGCCAAGGTGAAATGGCAGGGTAACTTGGATACAGGTCAGAATCCTCCGCTTGTCGACGATGAACTTGCCATCCACGGTAACAACTATGAGGGCGGTTGGAGAGTGATCAGTGAGCAACGGTACGATAACTACTGGAACTACATCAAGAACGACGGGAACAGGCTTCTCGATAAGTGGAAGTCCGGAGACTATTCTGAAAGCTATATTGAAGGCCTGTTGAACGGAAAGGCCGAGAACGCTCAGAAGAGGTACAGTGAGTCTCCGTTGGCGAATGCTGAGGTCTTGGATTCCAGCTTCCAGAGTGGAGCATTGAAGGCCGATATGGATAGTCGATTGGCTTACCCAGAGTTCAATGTCTATGTTGATGCCGGTGAGAACGGGTACATCACTGTCTCGAAGCCGGTTGGGAAGCCGAAGATTGTTTCATCGAGTGGTGCCAGTTTCGGTGAGCT**t**AGTAGCGGCAGGATCTCTGTCGACGTGAAGAACGTGGGCGGGGCGGAAGGCAGTTTCTCAGCCCGTGCTGGCAGCTGTAGTCAGTACTTCCAGGCGGATGCTCTTCAGAATACGAAAAGGGTTGCTCCCGGTGAGACTGCGTCCTACAGTTTCCGGGTTACGTTCACTTCGACGAGTATGCAGCAGGCCTCGTATACAGGTCGATGCAGTATCACTGTAGAGGATACGGGGAGTGGTCGAGAGGTTTCGACCAGTGTCTCGGTGGAGGCTACGCAACAGTCTGAATGTACTCAGGGCAAGGAGATTGTGAAGCAGAAGAACGGGAACGACGTCATCTACAGTTGTCCTGACGGTCTCAAGATTCAGAAGCAGGATACGTGTACGGGAGAGTTGAAGGCGGTTTTCGTCAACAACGATATCCAATACGATTGTAGGGAGGAAGGTAC**t**GGTGGTGGATCTGGTGGAGGCCTGTTCGGTAAACGGTTCACTCTCCCAATCACCGGTACTCAACTGAGTAATCCTCTGAATGCTTTCGAGAATATCTGGAGCGGTGACGCGAATGCGTTGAACTGGTTGCAGGTGTTTGTAACGTTCATCGCGTTCCTCGGCGGGTTCGCCTTGGTCGGTGTCAAGCTCGGTAAAATTGTAGACGGACTTGCGACAGAGTTCATCCCGGTAAAGGATTCCCACGTTAGACTGGTGATAGGCCTTCTCGGTGGAGGAATGATCGCAACAGCGGTCTATCAATTGGTCACAGATCCGTTAGGCCTCTTGGTCACGGTACTGGGCTTGGTTGTGATGGGTTATCTGTATCTCAGTGCGAGTGCTCCTGAAATCAACCTCCTAGAGGGCCCGCGGTTCGAAGGTAAGCCTATCCCTAACCCTCTCCTCGGTCTCGATTCTACGCGTACCGGTCATCATCACCATCACCATTGAgagctcgcatgcactagt

>H.natansFusexin1-V5-6His (pBPT03)EDITED

From 1 to 2022.

ggtaccATGTTCACTGCATTTGCGGCAGCAGATACTACTTCGGTGGATACTGTCTCGTATAAGAGCAACAGCGAGTTCTTCGACGGAGAA 90

M F T A F A A A D T T S V D T V S Y K S N S E F F D G E 30

GTTCTTCAGGCTGGTTATGTCTCGAACTTTGCGACTGACAAGATTAACGTCCATCTCGGTGCCAGTGAGATTGAGAGCAAGACCGGAGCT 180

V L Q A G Y V S N F A T D K I N V H L G A S E I E S K T G A 60

ACTGCGGAAGAAGACTTGACTTTGAGTGTTTCTCACCAGAACACCTACGCTCGCTACCCGCTCCAGGAGACCGGTTTGAAGAAGATCTAC 270

T A E E D L T L S V S H Q N T Y A R Y P L Q E T G L K K I Y 90

GGCTGGGAGGGGATGAAGAAGACATTCGATACGAAAGACCAGCTATGGAACTGGGTTACCTCAAACTGTGCTGACTTCACTGAGGGCGGA 360

G W E G M K K T F D T K D Q L W N W V T S N C A D F T E G G 120

GTAGCGATCGGAGATAGAACCAGTAAGGTCGAGGCCGCTGCCAAGAGCTGGTACGATTACTGGACAGGAAGTTACAATTACCAGGCCTTC 450

V A I G D R T S K V E A A A K S W Y D Y W T G S Y N Y Q A F 150

TGCCTGCGGAAGAACGGGTACTACGGAGATGTAGCTGATATCGGCTCCCCGGATGAAATCTTCAGAACCGAGTGGAGGCTGCAAGCAGGC 540

C L R K N G Y Y G D V A D I G S P D E I F R T E W R L Q A G 180

GATAAGAATCCGCAGACTGCTATAATAACAAATGGGGATGGTGGATCAGGTGTTGTCTCCAATCTTGGCCGGTATGCCAAGGTGAAATGG 630

D K N P Q T A I I T N G D G G S G V V S N L G R Y A K V K W 210

CAGGGTAACTTGGATACAGGTCAGAATCCTCCGCTTGTCGACGATGAACTTGCCATCCACGGTAACAACTATGAGGGCGGTTGGAGAGTG 720

Q G N L D T G Q N P P L V D D E L A I H G N N Y E G G W R V 240

ATCAGTGAGCAACGGTACGATAACTACTGGAACTACATCAAGAACGACGGGAACAGGCTTCTCGATAAGTGGAAGTCCGGAGACTATTCT 810

I S E Q R Y D N Y W N Y I K N D G N R L L D K W K S G D Y S 270

GAAAGCTATATTGAAGGCCTGTTGAACGGAAAGGCCGAGAACGCTCAGAAGAGGTACAGTGAGTCTCCGTTGGCGAATGCTGAGGTCTTG 900

E S Y I E G L L N G K A E N A Q K R Y S E S P L A N A E V L 300

GATTCCAGCTTCCAGAGTGGAGCATTGAAGGCCGATATGGATAGTCGATTGGCTTACCCAGAGTTCAATGTCTATGTTGATGCCGGTGAG 990

D S S F Q S G A L K A D M D S R L A Y P E F N V Y V D A G E 330

AACGGGTACATCACTGTCTCGAAGCCGGTTGGGAAGCCGAAGATTGTTTCATCGAGTGGTGCCAGTTTCGGTGAGCTtAGTAGCGGCAGG 1080

N G Y I T V S K P V G K P K I V S S S G A S F G E L S S G R 360

ATCTCTGTCGACGTGAAGAACGTGGGCGGGGCGGAAGGCAGTTTCTCAGCCCGTGCTGGCAGCTGTAGTCAGTACTTCCAGGCGGATGCT 1170

I S V D V K N V G G A E G S F S A R A G S C S Q Y F Q A D A 390

CTTCAGAATACGAAAAGGGTTGCTCCCGGTGAGACTGCGTCCTACAGTTTCCGGGTTACGTTCACTTCGACGAGTATGCAGCAGGCCTCG 1260

L Q N T K R V A P G E T A S Y S F R V T F T S T S M Q Q A S 420

TATACAGGTCGATGCAGTATCACTGTAGAGGATACGGGGAGTGGTCGAGAGGTTTCGACCAGTGTCTCGGTGGAGGCTACGCAACAGTCT 1350

Y T G R C S I T V E D T G S G R E V S T S V S V E A T Q Q S 450

GAATGTACTCAGGGCAAGGAGATTGTGAAGCAGAAGAACGGGAACGACGTCATCTACAGTTGTCCTGACGGTCTCAAGATTCAGAAGCAG 1440

E C T Q G K E I V K Q K N G N D V I Y S C P D G L K I Q K Q 480

GATACGTGTACGGGAGAGTTGAAGGCGGTTTTCGTCAACAACGATATCCAATACGATTGTAGGGAGGAAGGTACtGGTGGTGGATCTGGT 1530

D T C T G E L K A V F V N N D I Q Y D C R E E G T G G G S G 510

GGAGGCCTGTTCGGTAAACGGTTCACTCTCCCAATCACCGGTACTCAACTGAGTAATCCTCTGAATGCTTTCGAGAATATCTGGAGCGGT 1620

G G L F G K R F T L P I T G T Q L S N P L N A F E N I W S G 540

GACGCGAATGCGTTGAACTGGTTGCAGGTGTTTGTAACGTTCATCGCGTTCCTCGGCGGGTTCGCCTTGGTCGGTGTCAAGCTCGGTAAA 1710

D A N A L N W L Q V F V T F I A F L G G F A L V G V K L G K 570

ATTGTAGACGGACTTGCGACAGAGTTCATCCCGGTAAAGGATTCCCACGTTAGACTGGTGATAGGCCTTCTCGGTGGAGGAATGATCGCA 1800

I V D G L A T E F I P V K D S H V R L V I G L L G G G M I A 600

ACAGCGGTCTATCAATTGGTCACAGATCCGTTAGGCCTCTTGGTCACGGTACTGGGCTTGGTTGTGATGGGTTATCTGTATCTCAGTGCG 1890

T A V Y Q L V T D P L G L L V T V L G L V V M G Y L Y L S A 630

AGTGCTCCTGAAATCAACCTCCTAGAGGGCCCGCGGTTCGAAGGTAAGCCTATCCCTAACCCTCTCCTCGGTCTCGATTCTACGCGTACC 1980

S A P E I N L L E G P R F E G K P I P N P L L G L D S T R T 660

GGTCATCATCACCATCACCATTGAgagctcgcatgcactagt 2022

G H H H H H H * 674

*Haloplanus natans* Fsx1 modified with **complete signal peptide**

**ATGGTGAAACGAGTGGGTAATTGTTGGAAGGCCTCAGTAGCGGCATTCTTCCTTCTC**ATGTTCACTGCATTTGCGGCAGCAGATACTACTTCGGTGGATACTGTCTCGTATAAGAGCAACAGCGAGTTCTTCGACGGAGAAGTTCTTCAGGCTGGTTATGTCTCGAACTTTGCGACTGACAAGATTAACGTCCATCTCGGTGCCAGTGAGATTGAGAGCAAGACCGGAGCTACTGCGGAAGAAGACTTGACTTTGAGTGTTTCTCACCAGAACACCTACGCTCGCTACCCGCTCCAGGAGACCGGTTTGAAGAAGATCTACGGCTGGGAGGGGATGAAGAAGACATTCGATACGAAAGACCAGCTATGGAACTGGGTTACCTCAAACTGTGCTGACTTCACTGAGGGCGGAGTAGCGATCGGAGATAGAACCAGTAAGGTCGAGGCCGCTGCCAAGAGCTGGTACGATTACTGGACAGGAAGTTACAATTACCAGGCCTTCTGCCTGCGGAAGAACGGGTACTACGGAGATGTAGCTGATATCGGCTCCCCGGATGAAATCTTCAGAACCGAGTGGAGGCTGCAAGCAGGCGATAAGAATCCGCAGACTGCTATAATAACAAATGGGGATGGTGGATCAGGTGTTGTCTCCAATCTTGGCCGGTATGCCAAGGTGAAATGGCAGGGTAACTTGGATACAGGTCAGAATCCTCCGCTTGTCGACGATGAACTTGCCATCCACGGTAACAACTATGAGGGCGGTTGGAGAGTGATCAGTGAGCAACGGTACGATAACTACTGGAACTACATCAAGAACGACGGGAACAGGCTTCTCGATAAGTGGAAGTCCGGAGACTATTCTGAAAGCTATATTGAAGGCCTGTTGAACGGAAAGGCCGAGAACGCTCAGAAGAGGTACAGTGAGTCTCCGTTGGCGAATGCTGAGGTCTTGGATTCCAGCTTCCAGAGTGGAGCATTGAAGGCCGATATGGATAGTCGATTGGCTTACCCAGAGTTCAATGTCTATGTTGATGCCGGTGAGAACGGGTACATCACTGTCTCGAAGCCGGTTGGGAAGCCGAAGATTGTTTCATCGAGTGGTGCCAGTTTCGGTGAGCTtAGTAGCGGCAGGATCTCTGTCGACGTGAAGAACGTGGGCGGGGCGGAAGGCAGTTTCTCAGCCCGTGCTGGCAGCTGTAGTCAGTACTTCCAGGCGGATGCTCTTCAGAATACGAAAAGGGTTGCTCCCGGTGAGACTGCGTCCTACAGTTTCCGGGTTACGTTCACTTCGACGAGTATGCAGCAGGCCTCGTATACAGGTCGATGCAGTATCACTGTAGAGGATACGGGGAGTGGTCGAGAGGTTTCGACCAGTGTCTCGGTGGAGGCTACGCAACAGTCTGAATGTACTCAGGGCAAGGAGATTGTGAAGCAGAAGAACGGGAACGACGTCATCTACAGTTGTCCTGACGGTCTCAAGATTCAGAAGCAGGATACGTGTACGGGAGAGTTGAAGGCGGTTTTCGTCAACAACGATATCCAATACGATTGTAGGGAGGAAGGTACtGGTGGTGGATCTGGTGGAGGCCTGTTCGGTAAACGGTTCACTCTCCCAATCACCGGTACTCAACTGAGTAATCCTCTGAATGCTTTCGAGAATATCTGGAGCGGTGACGCGAATGCGTTGAACTGGTTGCAGGTGTTTGTAACGTTCATCGCGTTCCTCGGCGGGTTCGCCTTGGTCGGTGTCAAGCTCGGTAAAATTGTAGACGGACTTGCGACAGAGTTCATCCCGGTAAAGGATTCCCACGTTAGACTGGTGATAGGCCTTCTCGGTGGAGGAATGATCGCAACAGCGGTCTATCAATTGGTCACAGATCCGTTAGGCCTCTTGGTCACGGTACTGGGCTTGGTTGTGATGGGTTATCTGTATCTCAGTGCGAGTGCTCCTGAAATCAACCTCCTAGAGGGCCCGCGGTTCGAAGGTAAGCCTATCCCTAACCCTCTCCTCGGTCTCGATTCTACGCGTACCGGTCATCATCACCATCACCATTAG

Synthesized plasmid: pBPT04

SAMEA2619974_10776_4

SAMEA.TARA-Fusexin1-V5-6His (pBPT04) EDITED

**Addition of restriction Ez. Sites**

**5’ KpnI, 3’ SacI, SphI, SpeI**

KpnI edited GGTACC to GGTTCC both encoding for Val

ggtaccATGTATAAAAGGAAGATAATCATACCAATATTATTATTTGTAATATTATCCCTAATAGTTAGTGCTTTCTCAACACTATCACTTGATAGGGTAGATTTTTCTACCAGGGGAGATGAGTTTGACCAACAGTGGTTATTATTAATTAGTGAAGATGGTAGAGCTGATAAAGCAGTGGTAACTAAAAAAGCAGAAGAAATTAAAGATGATAAAATCCACGCTAAAAATGATTTAACTATCAAAACTGAGATAGATAAAAATAGTTGTGTGTATACAATCCAAAATATAAACCAACCTATTAGTAGGTTAGATTATACAAAAAAGACTGTATGGAAATTCTGGGATATACCTAATGAGATAAAGAGTTGTGAGCAAAGAGAAGGGTATTATTGGGCTTTTAACCTTGGATTTGATTTTAATGTTTATTGTTTCTTCACTCCAGCTGGTAAAGATTCGTTTGTTGGTAGGATTTCCGATAAAAAGTATGATTTTAATACCAAAATCACCCTCACATCAGGTGGTGAAAGTAAATCAGTAGATATATCAAACTCAAATAGAGTAAGTAATTCAGTTCCAGATTATTTTGTAGCAAAATGGCACGGTAGTCTGTCAACTGGAGCAGAATGTCCAGATATCACTTCCCTATCTCCCATATATGATAGAGATAGTGGTGAATGGAAGTTGGGAGAAAAATCATCAATTTCTAACTATCAATCTTTTCATTCTTCAGGTATGAAAGAATGTCTTAACAGTTATATAATTGCCAGGACTACTGGAGGACTTATAGTAGAAACACCAGATAGTTGTAAGAAAAGGTATAATGATTTAGTAGAAAAAGCACAGTCACCAGAAGTATTTAATATTAAAGGTTCTACTGGTAGTATGACAACACAATCATCAGAGCTTGGTGAGTTAGTGGTTGAAGATGTTCAACCTTTCCAATTCCCAGTAATAAGTTTGAAAATCAATGCAGATTGGATTGGAATTGTCCAACCTATCACAAAACCTACAATATTAAGTTGTGAAAGTAGTAAGTTTAAACAAGGAGAGATAGGTGAGATAGATGTTGAGGTTAAAAATAAGGGAGAGAGTGGTTCAGTTTCCATATCAACTGTTTGTTTATCTCCCTTTAAATCAGTTGGAACAACACCCATAATAAGGTTAAAGAAGGGTGAAAGTAAATCAATTACTGTTCCAATCACCGTTAGTACAAAAGAAGATGTAAGTAAAACATGTAGTGTAACAGTTCAAGATGAATCAGACCCAAGTGTGAGAGTTAGTAAAAGATGTGATGTTAGTGCAAGTGGAGTTGTATTGTGTGAAGCAGGAAAGAAAAGGTGTAGTGGTAGATTTATAGAACAATGTAAAAGTAGTGGTAGTGAATATGGGTTAATTGAAGAGTGTGAATTAGATTGTAAGTTGGATAAGTACGGACAACCATTTTGTCCAGAAGTTACACCCCCACCTCCACCTCCACCAAACGGAAATGGAGATAAATGTGAACCAATATGGGCAATAGCAAAGATAACTATCATACCAGATTTTATTTGTGAGATGGAGAAGGTtCCTTACCTAAAAGAGGGTATTAGTGGTTTGGTTGGATTTGTGATGTTTATAATATTAATATTTATGTTGAAGAATTTAATCGGATTTGAGAACATACCACAAAGGTTAATGGTATTAGGTTTCTCATTGATAATTGCAATATTATTGGGTTTTTTGTTTTATTATTTGTTTTGGTTTGGAGTTGCATTAATAGTGGCGTTAGTTGTGATGTTTTTTGTAATTAAAATAATCCTCGGAAAGGTGGGGTTGCTAGAGGGCCCGCGGTTCGAAGGTAAGCCTATCCCTAACCCTCTCCTCGGTCTCGATTCTACGCGTACCGGTCATCATCACCATCACCATTGAgagctcgcatgcactagt

SAMEA.TARA-Fusexin1-V5-6His (pBPT04) EDITED

From 1 to 1929.

ggtaccATGTATAAAAGGAAGATAATCATACCAATATTATTATTTGTAATATTATCCCTAATAGTTAGTGCTTTCTCAACACTATCACTT 90

M Y K R K I I I P I L L F V I L S L I V S A F S T L S L 30

GATAGGGTAGATTTTTCTACCAGGGGAGATGAGTTTGACCAACAGTGGTTATTATTAATTAGTGAAGATGGTAGAGCTGATAAAGCAGTG 180

D R V D F S T R G D E F D Q Q W L L L I S E D G R A D K A V 60

GTAACTAAAAAAGCAGAAGAAATTAAAGATGATAAAATCCACGCTAAAAATGATTTAACTATCAAAACTGAGATAGATAAAAATAGTTGT 270

V T K K A E E I K D D K I H A K N D L T I K T E I D K N S C 90

GTGTATACAATCCAAAATATAAACCAACCTATTAGTAGGTTAGATTATACAAAAAAGACTGTATGGAAATTCTGGGATATACCTAATGAG 360

V Y T I Q N I N Q P I S R L D Y T K K T V W K F W D I P N E 120

ATAAAGAGTTGTGAGCAAAGAGAAGGGTATTATTGGGCTTTTAACCTTGGATTTGATTTTAATGTTTATTGTTTCTTCACTCCAGCTGGT 450

I K S C E Q R E G Y Y W A F N L G F D F N V Y C F F T P A G 150

AAAGATTCGTTTGTTGGTAGGATTTCCGATAAAAAGTATGATTTTAATACCAAAATCACCCTCACATCAGGTGGTGAAAGTAAATCAGTA 540

K D S F V G R I S D K K Y D F N T K I T L T S G G E S K S V 180

GATATATCAAACTCAAATAGAGTAAGTAATTCAGTTCCAGATTATTTTGTAGCAAAATGGCACGGTAGTCTGTCAACTGGAGCAGAATGT 630

D I S N S N R V S N S V P D Y F V A K W H G S L S T G A E C 210

CCAGATATCACTTCCCTATCTCCCATATATGATAGAGATAGTGGTGAATGGAAGTTGGGAGAAAAATCATCAATTTCTAACTATCAATCT 720

P D I T S L S P I Y D R D S G E W K L G E K S S I S N Y Q S 240

TTTCATTCTTCAGGTATGAAAGAATGTCTTAACAGTTATATAATTGCCAGGACTACTGGAGGACTTATAGTAGAAACACCAGATAGTTGT 810

F H S S G M K E C L N S Y I I A R T T G G L I V E T P D S C 270

AAGAAAAGGTATAATGATTTAGTAGAAAAAGCACAGTCACCAGAAGTATTTAATATTAAAGGTTCTACTGGTAGTATGACAACACAATCA 900

K K R Y N D L V E K A Q S P E V F N I K G S T G S M T T Q S 300

TCAGAGCTTGGTGAGTTAGTGGTTGAAGATGTTCAACCTTTCCAATTCCCAGTAATAAGTTTGAAAATCAATGCAGATTGGATTGGAATT 990

S E L G E L V V E D V Q P F Q F P V I S L K I N A D W I G I 330

GTCCAACCTATCACAAAACCTACAATATTAAGTTGTGAAAGTAGTAAGTTTAAACAAGGAGAGATAGGTGAGATAGATGTTGAGGTTAAA 1080

V Q P I T K P T I L S C E S S K F K Q G E I G E I D V E V K 360

AATAAGGGAGAGAGTGGTTCAGTTTCCATATCAACTGTTTGTTTATCTCCCTTTAAATCAGTTGGAACAACACCCATAATAAGGTTAAAG 1170

N K G E S G S V S I S T V C L S P F K S V G T T P I I R L K 390

AAGGGTGAAAGTAAATCAATTACTGTTCCAATCACCGTTAGTACAAAAGAAGATGTAAGTAAAACATGTAGTGTAACAGTTCAAGATGAA 1260

K G E S K S I T V P I T V S T K E D V S K T C S V T V Q D E 420

TCAGACCCAAGTGTGAGAGTTAGTAAAAGATGTGATGTTAGTGCAAGTGGAGTTGTATTGTGTGAAGCAGGAAAGAAAAGGTGTAGTGGT 1350

S D P S V R V S K R C D V S A S G V V L C E A G K K R C S G 450

AGATTTATAGAACAATGTAAAAGTAGTGGTAGTGAATATGGGTTAATTGAAGAGTGTGAATTAGATTGTAAGTTGGATAAGTACGGACAA 1440

R F I E Q C K S S G S E Y G L I E E C E L D C K L D K Y G Q 480

CCATTTTGTCCAGAAGTTACACCCCCACCTCCACCTCCACCAAACGGAAATGGAGATAAATGTGAACCAATATGGGCAATAGCAAAGATA 1530

P F C P E V T P P P P P P P N G N G D K C E P I W A I A K I 510

ACTATCATACCAGATTTTATTTGTGAGATGGAGAAGGTtCCTTACCTAAAAGAGGGTATTAGTGGTTTGGTTGGATTTGTGATGTTTATA 1620

T I I P D F I C E M E K V P Y L K E G I S G L V G F V M F I 540

ATATTAATATTTATGTTGAAGAATTTAATCGGATTTGAGAACATACCACAAAGGTTAATGGTATTAGGTTTCTCATTGATAATTGCAATA 1710

I L I F M L K N L I G F E N I P Q R L M V L G F S L I I A I 570

TTATTGGGTTTTTTGTTTTATTATTTGTTTTGGTTTGGAGTTGCATTAATAGTGGCGTTAGTTGTGATGTTTTTTGTAATTAAAATAATC 1800

L L G F L F Y Y L F W F G V A L I V A L V V M F F V I K I I 600

CTCGGAAAGGTGGGGTTGCTAGAGGGCCCGCGGTTCGAAGGTAAGCCTATCCCTAACCCTCTCCTCGGTCTCGATTCTACGCGTACCGGT 1890

L G K V G L L E G P R F E G K P I P N P L L G L D S T R T G 630

CATCATCACCATCACCATTGAgagctcgcatgcactagt 1929

H H H H H H * 643

Synthesized plasmid: pBPT05

LKMP01000007_1 (Nanohaloarchaea B1-Br10_U2g21 LB-BRINE-C121)

>NanohaloarchFusexin1-V5-6His (pBPT05)EDITED

**Addition of restriction Ez. Sites**

**5’ KpnI, 3’ SacI, SphI, SpeI**

ggtaccATGTCTACGTTTTTGATAGTTCCAGCTTCTGTATCTTATGTATCGGAAGTAGACTATATTGAAACAACTTCAGAGTTTTTTGACCAGGGCCACGTAGTTATCGAATTTGTTATTGATGGCGCTGATGAGAAAATACATGCAGATCTAGAACCTAGCCAGTTATCAGATTTATCTGATCAAAATACGAATCAAAGTGTTGAGATTAGCGCGAAAGTATCCGATGTCTATGCCGAGTATGAAGTAGAAGATAGAGGTCTCAGGCCTTTAAAACCTATAGAATACACGAGAACTACTCATTCCACAAAAGAAAAAGCTCAGATTTGGGCCGATAATAACTGCCTTGATCTTGATAATAGCGGAGCACCCAACTATAATACTTATAGAAGAGGATTGGTTAACCCCTGGGGAGACTGGATTGTGAATTGTGCGATACCTAATATGAGCCAATCTACTTTTAACGTTGGTTTCTTACAGAGTCCTCCAGATACGTTATTTGAGTCTGAATGGATTATAGCTGTTGAAGGTGAATCTGCCGAAACAGTTCGATTATCTAATAGTGGCGCAGAACATGGAAGTTTGGAAAGAGTGAATGATAATGTTCAACTCGAGTTTGTTTCATTGAAAGATACCGGGAGTAATGCTCCTAACCCTCAAAATACTCTAGCTGCTTTTTCAAGCACAGATGGCTGGAGATTAATTGAAAAAGAAGAATTTGATAGCTACATAACTTATATTGAAAATGAATCTCCTAAAAAAGTTGAAGAGATGCGAGAACAAGATAATTTCAATATAGAAGACTGGACTGATAAGAAAAACAAAGAAGCCAGAGATAACTTTTTGAGCGCATCAAGAGAATACGAAGAATCTCCTATTCAAGGGTCAAACGTATCTGATGGCAAAAACAAATTTCAAAATGGGATTTTTCAATATAGGCCTGAATCAGAGTTCGAATGGTTTATCTCAGAGTTTAACGTTAGATTAAATGGTGATTTTGTAGGACTACAGCGAAAATTTGCAGATTTAGAGATTATAAATATAGAAGATACCAAAGTTAATGGTCTTTCAAGCGGTTCTGTGGAAGTAAAAGTTGCAAATAATGGCAATGGAACCGGCGCTTTTGAAGTTTCAGCTTCCTGTGATAAATTTGATACAGTTGGAGTAAGTAGTCGCACATCATTAAAATCTGGAGAATCTGACACAGTATTTCTAAATTTGGATACTGACGGTTCTGAAGAGTTCTATGAAACTTGTAATCTCAATATTAAGGACCTTGAAACTACTTCAATCGTTTCTAGCTCATTTACTGCATCTTACACTCCTGAAAAAGAATGTACACCAGGAAGACAGTCAGTCAGAATAGAGAACGGACAAGAAGCAATTTACGAATGTGCTGAAGACGGAATTTCCATATTTGAGGTTGATGCTTGCGAATTGGACGAAAGAGCTAAACCTTACGGTGAAGGATATGAGTGTCAAAAAGTTGAAAGTCTTCGGGAAATTGAAGCACCTCAAGACTGCGAAATTTCCATACTTCCTAGCTTCCTGAATAACTTATTCGCTAATGACTATACCTTTACAAATCCAATTTGCGCCACGGAAAACAGGTTAAATGATTTTAATAATAGTATTGAATCTGCAAAAAACATGTTTACTATGCTATTCATTATAACAGGCGGATTAATAGCGGCAATACTAAGTTGGAAAATCTCTAAGACCTTTACTAAACACTACCTTAGCTTACTTAAACAGCAAAAACTAGAGGGCCCGCGGTTCGAAGGTAAGCCTATCCCTAACCCTCTCCTCGGTCTCGATTCTACGCGTACCGGTCATCATCACCATCACCATTGAgagctcgcatgcactagt

>NanohaloarchFusexin1-V5-6His (pBPT05)EDITED

From 1 to 1872.

ggtaccATGTCTACGTTTTTGATAGTTCCAGCTTCTGTATCTTATGTATCGGAAGTAGACTATATTGAAACAACTTCAGAGTTTTTTGAC 90

M S T F L I V P A S V S Y V S E V D Y I E T T S E F F D 30

CAGGGCCACGTAGTTATCGAATTTGTTATTGATGGCGCTGATGAGAAAATACATGCAGATCTAGAACCTAGCCAGTTATCAGATTTATCT 180

Q G H V V I E F V I D G A D E K I H A D L E P S Q L S D L S 60

GATCAAAATACGAATCAAAGTGTTGAGATTAGCGCGAAAGTATCCGATGTCTATGCCGAGTATGAAGTAGAAGATAGAGGTCTCAGGCCT 270

D Q N T N Q S V E I S A K V S D V Y A E Y E V E D R G L R P 90

TTAAAACCTATAGAATACACGAGAACTACTCATTCCACAAAAGAAAAAGCTCAGATTTGGGCCGATAATAACTGCCTTGATCTTGATAAT 360

L K P I E Y T R T T H S T K E K A Q I W A D N N C L D L D N 120

AGCGGAGCACCCAACTATAATACTTATAGAAGAGGATTGGTTAACCCCTGGGGAGACTGGATTGTGAATTGTGCGATACCTAATATGAGC 450

S G A P N Y N T Y R R G L V N P W G D W I V N C A I P N M S 150

CAATCTACTTTTAACGTTGGTTTCTTACAGAGTCCTCCAGATACGTTATTTGAGTCTGAATGGATTATAGCTGTTGAAGGTGAATCTGCC 540

Q S T F N V G F L Q S P P D T L F E S E W I I A V E G E S A 180

GAAACAGTTCGATTATCTAATAGTGGCGCAGAACATGGAAGTTTGGAAAGAGTGAATGATAATGTTCAACTCGAGTTTGTTTCATTGAAA 630

E T V R L S N S G A E H G S L E R V N D N V Q L E F V S L K 210

GATACCGGGAGTAATGCTCCTAACCCTCAAAATACTCTAGCTGCTTTTTCAAGCACAGATGGCTGGAGATTAATTGAAAAAGAAGAATTT 720

D T G S N A P N P Q N T L A A F S S T D G W R L I E K E E F 240

GATAGCTACATAACTTATATTGAAAATGAATCTCCTAAAAAAGTTGAAGAGATGCGAGAACAAGATAATTTCAATATAGAAGACTGGACT 810

D S Y I T Y I E N E S P K K V E E M R E Q D N F N I E D W T 270

GATAAGAAAAACAAAGAAGCCAGAGATAACTTTTTGAGCGCATCAAGAGAATACGAAGAATCTCCTATTCAAGGGTCAAACGTATCTGAT 900

D K K N K E A R D N F L S A S R E Y E E S P I Q G S N V S D 300

GGCAAAAACAAATTTCAAAATGGGATTTTTCAATATAGGCCTGAATCAGAGTTCGAATGGTTTATCTCAGAGTTTAACGTTAGATTAAAT 990

G K N K F Q N G I F Q Y R P E S E F E W F I S E F N V R L N 330

GGTGATTTTGTAGGACTACAGCGAAAATTTGCAGATTTAGAGATTATAAATATAGAAGATACCAAAGTTAATGGTCTTTCAAGCGGTTCT 1080

G D F V G L Q R K F A D L E I I N I E D T K V N G L S S G S 360

GTGGAAGTAAAAGTTGCAAATAATGGCAATGGAACCGGCGCTTTTGAAGTTTCAGCTTCCTGTGATAAATTTGATACAGTTGGAGTAAGT 1170

V E V K V A N N G N G T G A F E V S A S C D K F D T V G V S 390

AGTCGCACATCATTAAAATCTGGAGAATCTGACACAGTATTTCTAAATTTGGATACTGACGGTTCTGAAGAGTTCTATGAAACTTGTAAT 1260

S R T S L K S G E S D T V F L N L D T D G S E E F Y E T C N 420

CTCAATATTAAGGACCTTGAAACTACTTCAATCGTTTCTAGCTCATTTACTGCATCTTACACTCCTGAAAAAGAATGTACACCAGGAAGA 1350

L N I K D L E T T S I V S S S F T A S Y T P E K E C T P G R 450

CAGTCAGTCAGAATAGAGAACGGACAAGAAGCAATTTACGAATGTGCTGAAGACGGAATTTCCATATTTGAGGTTGATGCTTGCGAATTG 1440

Q S V R I E N G Q E A I Y E C A E D G I S I F E V D A C E L 480

GACGAAAGAGCTAAACCTTACGGTGAAGGATATGAGTGTCAAAAAGTTGAAAGTCTTCGGGAAATTGAAGCACCTCAAGACTGCGAAATT 1530

D E R A K P Y G E G Y E C Q K V E S L R E I E A P Q D C E I 510

TCCATACTTCCTAGCTTCCTGAATAACTTATTCGCTAATGACTATACCTTTACAAATCCAATTTGCGCCACGGAAAACAGGTTAAATGAT 1620

S I L P S F L N N L F A N D Y T F T N P I C A T E N R L N D 540

TTTAATAATAGTATTGAATCTGCAAAAAACATGTTTACTATGCTATTCATTATAACAGGCGGATTAATAGCGGCAATACTAAGTTGGAAA 1710

F N N S I E S A K N M F T M L F I I T G G L I A A I L S W K 570

ATCTCTAAGACCTTTACTAAACACTACCTTAGCTTACTTAAACAGCAAAAACTAGAGGGCCCGCGGTTCGAAGGTAAGCCTATCCCTAAC 1800

I S K T F T K H Y L S L L K Q Q K L E G P R F E G K P I P N 600

CCTCTCCTCGGTCTCGATTCTACGCGTACCGGTCATCATCACCATCACCATTGAgagctcgcatgcactagt 1872

P L L G L D S T R T G H H H H H H * 624

Synthesized plasmid: pBPT06

3300014206-Ga0172377-10000119-870930-129 (Methanomicrobia)

3300014206Sub.Fusexin1-V5-6His (pBPT06)EDITED

**Addition of restriction Ez. Sites**

**5’ KpnI, 3’ SacI, SphI, SpeI**

ggtaccATGAACAAAATGAGAAGTACGATAAAAATATCAATATTTTTTTTATTGATATTCGCACTAACAATACCAATACAAAATGTTAGTGCAGAAGTAGATGGATTTACATTGAAAGCTGCATCTCTGTCACATCCAGTATCAAACAATAAAGATTTAGAAGATGCAAACTTGACAATTCTTGTTGGAACTGGAGGAGGGCAATCACTACATGGAAAATTTGGAAATGAACAACTAACTCAATGGATTCCAAATTTATTATTCAAGTATCCACTGATATTTGAAATGAGTCCTATAATAGAAACTGCAGACTATCCAATAAGAAATCAAGCACATAGCGAATTAAAAAGACTTGAATATGAAATAAAAGGAAACTGGTTATTTTCAGAAAAATGTAATTCATCAATGTTATATTGTTTCGATGTCGGATCTGGAAAAATCATTGAAATAAAAGAAGTCGATGTTGGAAGATATGGATCAATAGATAATCCAAATTTATTTTGGGGAGGAAATGTAAAAGTAACAATAAATGGGATTCCATACTCAAAAGATATTTCAAGTAACATAGAAAGTGTACAATTTAATTCTATAACAGGAGATTTTGTTGCACTAATAAGATGGGTTGGTTCTACTGTAACAGGACAAGGACTTCCTGATCAACACAATAATGTTCCTATATATAATAAAGACAACGAAAAATGGTCAATATCAAATATTGCAACTTTCAGAGATTACGAACGATCATTACTAACAACTGAAATAGAGTTGAATGCTAAACAAGAACAAAAGAAGGCATTTGATAATAAATTCTATGAATATGCAGCAGAAGAATATTTTGATAAGTATTCGATATGTAAAGATGATCAATGTTCACCGATTGTAAGTATTATAAATACACACAATGCAAAGCTAACAATATTGTTAAATGAACGAAAGAAAATTGGTTATGAATCAGACATATCATCTAATCAACTAACTACAAAAGATGGAAATGTTTTACTTGTATTAAATAGAAGAATAAATATTCCTGAATTTACAATAATAACAACTGCTAAGAGTGTAGGAGCATACATACCAACTGGAGAATTCCAGATAATAGATATAACATCACCAACTTTTAGTTCTGGAGATAGTAATGGAAAAGTTGATATAAAAATAAGAAATAATGGAGATTATAAAGGAACTGCTACTGCAACTTTAATAGATGAAACTGATACATTTAAACAAATATCGAATTCAAAATCGTTAGAAACGACACTCGATCCTGGTAAAGAAGGAACTATGACAATGTACATATCAAGTGGTACAATATCGAGTGATATTACCAAAACTGCTAAGATAGATGTATATGATATAAATAGTCCAAACAATGTAACATCAAAAGAATTCACTGCATCTGTAACAACACCAAAAATGTGTCAACCAGATTCTCAAAGATTAGACAATAAAATTGTATACACATGTAATACAGATGGAACTGCTGAATATGTAACATTAGATTGTTCAAATGGATTACCAAACTATGAAAATAATACATATACATGCACTCAAATCGAATATAATAAAGGTTTGGTGATGACAAATGTCAAAAATCCAATCAAAGTAGTCGAGAAACAAAACAAAACTAAAAAAGAAGATAAAAAAGATGAGTCATCAACTGCATACAATTTTTTAAAATGGATGACAGGTTTGGTGTTATTATACTTTGCAATCGCCTTTATAAGAAAACATAATAACAAAGATCAAATAATCACAAAAGAAAACACAAGATTATCGATAATACAATCGTCCTTAGTGTTAGGAATATTTTATATATATTTACAATTTAAGTTTCTAACAGAAACACTTGGTTCGACATTCTGGATTATAGTTATAATATCTGGAATATTGGTTGGACTTGTTGACATGTATCTAAAAATGACAATACATATGAATATTCCAAAAATATATGTGCTAACAGCAGGTATAATATTATTTATAATTCTATCTCAAATAGCTGTTGGAATAAAAGAAACTGCTTGTAATAGTTGGACAACAAAATGGTTAGCAAATTGGATGTTTGACACATGCAAGGAATGGAGTATAAATGACTATCTACCAGAATGGCTGAAACTAGAGGGCCCGCGGTTCGAAGGTAAGCCTATCCCTAACCCTCTCCTCGGTCTCGATTCTACGCGTACCGGTCATCATCACCATCACCATTGAgagctcgcatgcactagt

3300014206Sub.Fusexin1-V5-6His (pBPT06)EDITED

From 1 to 2223.

ggtaccATGAACAAAATGAGAAGTACGATAAAAATATCAATATTTTTTTTATTGATATTCGCACTAACAATACCAATACAAAATGTTAGT 90

M N K M R S T I K I S I F F L L I F A L T I P I Q N V S 30

GCAGAAGTAGATGGATTTACATTGAAAGCTGCATCTCTGTCACATCCAGTATCAAACAATAAAGATTTAGAAGATGCAAACTTGACAATT 180

A E V D G F T L K A A S L S H P V S N N K D L E D A N L T I 60

CTTGTTGGAACTGGAGGAGGGCAATCACTACATGGAAAATTTGGAAATGAACAACTAACTCAATGGATTCCAAATTTATTATTCAAGTAT 270

L V G T G G G Q S L H G K F G N E Q L T Q W I P N L L F K Y 90

CCACTGATATTTGAAATGAGTCCTATAATAGAAACTGCAGACTATCCAATAAGAAATCAAGCACATAGCGAATTAAAAAGACTTGAATAT 360

P L I F E M S P I I E T A D Y P I R N Q A H S E L K R L E Y 120

GAAATAAAAGGAAACTGGTTATTTTCAGAAAAATGTAATTCATCAATGTTATATTGTTTCGATGTCGGATCTGGAAAAATCATTGAAATA 450

E I K G N W L F S E K C N S S M L Y C F D V G S G K I I E I 150

AAAGAAGTCGATGTTGGAAGATATGGATCAATAGATAATCCAAATTTATTTTGGGGAGGAAATGTAAAAGTAACAATAAATGGGATTCCA 540

K E V D V G R Y G S I D N P N L F W G G N V K V T I N G I P 180

TACTCAAAAGATATTTCAAGTAACATAGAAAGTGTACAATTTAATTCTATAACAGGAGATTTTGTTGCACTAATAAGATGGGTTGGTTCT 630

Y S K D I S S N I E S V Q F N S I T G D F V A L I R W V G S 210

ACTGTAACAGGACAAGGACTTCCTGATCAACACAATAATGTTCCTATATATAATAAAGACAACGAAAAATGGTCAATATCAAATATTGCA 720

T V T G Q G L P D Q H N N V P I Y N K D N E K W S I S N I A 240

ACTTTCAGAGATTACGAACGATCATTACTAACAACTGAAATAGAGTTGAATGCTAAACAAGAACAAAAGAAGGCATTTGATAATAAATTC 810

T F R D Y E R S L L T T E I E L N A K Q E Q K K A F D N K F 270

TATGAATATGCAGCAGAAGAATATTTTGATAAGTATTCGATATGTAAAGATGATCAATGTTCACCGATTGTAAGTATTATAAATACACAC 900

Y E Y A A E E Y F D K Y S I C K D D Q C S P I V S I I N T H 300

AATGCAAAGCTAACAATATTGTTAAATGAACGAAAGAAAATTGGTTATGAATCAGACATATCATCTAATCAACTAACTACAAAAGATGGA 990

N A K L T I L L N E R K K I G Y E S D I S S N Q L T T K D G 330

AATGTTTTACTTGTATTAAATAGAAGAATAAATATTCCTGAATTTACAATAATAACAACTGCTAAGAGTGTAGGAGCATACATACCAACT 1080

N V L L V L N R R I N I P E F T I I T T A K S V G A Y I P T 360

GGAGAATTCCAGATAATAGATATAACATCACCAACTTTTAGTTCTGGAGATAGTAATGGAAAAGTTGATATAAAAATAAGAAATAATGGA 1170

G E F Q I I D I T S P T F S S G D S N G K V D I K I R N N G 390

GATTATAAAGGAACTGCTACTGCAACTTTAATAGATGAAACTGATACATTTAAACAAATATCGAATTCAAAATCGTTAGAAACGACACTC 1260

D Y K G T A T A T L I D E T D T F K Q I S N S K S L E T T L 420

GATCCTGGTAAAGAAGGAACTATGACAATGTACATATCAAGTGGTACAATATCGAGTGATATTACCAAAACTGCTAAGATAGATGTATAT 1350

D P G K E G T M T M Y I S S G T I S S D I T K T A K I D V Y 450

GATATAAATAGTCCAAACAATGTAACATCAAAAGAATTCACTGCATCTGTAACAACACCAAAAATGTGTCAACCAGATTCTCAAAGATTA 1440

D I N S P N N V T S K E F T A S V T T P K M C Q P D S Q R L 480

GACAATAAAATTGTATACACATGTAATACAGATGGAACTGCTGAATATGTAACATTAGATTGTTCAAATGGATTACCAAACTATGAAAAT 1530

D N K I V Y T C N T D G T A E Y V T L D C S N G L P N Y E N 510

AATACATATACATGCACTCAAATCGAATATAATAAAGGTTTGGTGATGACAAATGTCAAAAATCCAATCAAAGTAGTCGAGAAACAAAAC 1620

N T Y T C T Q I E Y N K G L V M T N V K N P I K V V E K Q N 540

AAAACTAAAAAAGAAGATAAAAAAGATGAGTCATCAACTGCATACAATTTTTTAAAATGGATGACAGGTTTGGTGTTATTATACTTTGCA 1710

K T K K E D K K D E S S T A Y N F L K W M T G L V L L Y F A 570

ATCGCCTTTATAAGAAAACATAATAACAAAGATCAAATAATCACAAAAGAAAACACAAGATTATCGATAATACAATCGTCCTTAGTGTTA 1800

I A F I R K H N N K D Q I I T K E N T R L S I I Q S S L V L 600

GGAATATTTTATATATATTTACAATTTAAGTTTCTAACAGAAACACTTGGTTCGACATTCTGGATTATAGTTATAATATCTGGAATATTG 1890

G I F Y I Y L Q F K F L T E T L G S T F W I I V I I S G I L 630

GTTGGACTTGTTGACATGTATCTAAAAATGACAATACATATGAATATTCCAAAAATATATGTGCTAACAGCAGGTATAATATTATTTATA 1980

V G L V D M Y L K M T I H M N I P K I Y V L T A G I I L F I 660

ATTCTATCTCAAATAGCTGTTGGAATAAAAGAAACTGCTTGTAATAGTTGGACAACAAAATGGTTAGCAAATTGGATGTTTGACACATGC 2070

I L S Q I A V G I K E T A C N S W T T K W L A N W M F D T C 690

AAGGAATGGAGTATAAATGACTATCTACCAGAATGGCTGAAACTAGAGGGCCCGCGGTTCGAAGGTAAGCCTATCCCTAACCCTCTCCTC 2160

K E W S I N D Y L P E W L K L E G P R F E G K P I P N P L L 720

GGTCTCGATTCTACGCGTACCGGTCATCATCACCATCACCATTGAgagctcgcatgcactagt 2223

G L D S T R T G H H H H H H * 741

Synthesized plasmid: pBPT07

3300014208-Ga0172379-10000243-871512-158(Methanomicrobia?)

>3300014208Sub.Fusexin1-V5-6His (pBPT07)EDITED

**Addition of restriction Ez. Sites**

**5’ KpnI, 3’ SacI, SphI, SpeI**

ggtaccATGAACCCAATAAAACCCATATTTCTATCAATACTCATAATCGCTATCTTAATTCCCTCCTCTGTCGCCTCCACTACAGCAGGATCAGGATTTACTGTAATGTCAATATCCCCATCTAAAATAATTTCCAACGATGAAGACTTAAAAACTGCCAATTTCATCGTAACAGTCGTAGCAAACGGAAATTCTCAATCTATCATCGGAGAACTCCTCACCGCCAATGACATCAATGCTAAACTCTCCGAATCAGGCTCCTCAGTTCGAGTTTCCTCCCCCTTTAAAATTGAAATGGGTTCAGTCAAAGAAACCCTAACTTACCCCATCCGTAACACAGGCGCAATTCGTAAATACGAATATGCATATTCATCTGAAGTTAATTTTGTTTCGGAAGCAACATGTCTATCAGGCTATTCATATTGTTATACAGTCGAGGCAGGTTCCACATGGTGGGGAAAAACATACAGAAGTCTCAATATTCGCCAAGCAAACAGCGCCGTCTCCTCTTCCCTCGGAGAACTCGATAATCCAAACATTAATTGGCAGGGCGATATAGTTCTAACAGCAGGACTAACTCGTGTATCAAAAACAATAGGTTCAATCGAATCCCTTGGAGCAGCAGATTTCTATAACAGTAACGAGTGGATAGCTCAAGCTACATGGACAGGTTCTTTACTCACAGGGCAAGCAACCCCCAATCCTTCTCTCTATAAAGCAACCTTTACTCCCGCCTTTAATCAATGGAGAATCTCCTCAAATTCCTACTATAATGACTATGCATCCTCCCTATCTGCAAAACAAGCATACTTCCCCTCAATGTTAAGCCAATATACTAAACAAGTGTGTCTTGATTCATACGGTTGTCCATCAGAAACTCCCCTAACAAACGCAATCAGCAATCATAATAGCATTGTAGACATCCTCTTATCTCAAAACACTAAAGTAACATACACCTCCTCTACAGGAGAAACTCTAGCCCAATCAACATCAGGAACTTCCTACTCAGGTAACGTAATCGAGACAACCAATCGTCGTATCTCCAATCCTGTAATCGTGTTCAAAGTCAAAGCATTATCCTTATCTCCCTACATTCCCGTCGGAAAACCACGCATCGAATCAATAACAACACCCGAATTCTCTAGCGGTGACAACAATGGAGTAGCAACAGTTCAATTCAAAAATATCGGTACTGAATCAGCAACATTCTCAGCAACATTCTTTGATCCCTCAGGAACTTTCCACCCCTCCTCTAATCAAGAATCCTCAAAAACTACATGCCCTGCCCTTTCCCAATGCATCATAAACGTCTTTATCTCCAGTGACTCCCCTACCAATGATCGTATCAGTAAAACTGCTACAATGACAGTCTATGACATAAACAAACCTTCCAATTCTGATACAAAAACCTTCGACATAATAATGTCACCTCCTAAAACCTGTATCCCTAGTTCCACTCGCACCTCAGGAAAACTCATCTATACCTGTAACGCCGACGGTTCAGGGGAAACAGTAATAGAATGCACAGGAACAATCTCCCTTCAAAACAGCAAATACATTTGCATAGGTGGTTCAGTAACTCCCAAACCCGGAACAACACCAGTACCGAAAACAATATTCGCAACCCTATCTGACTTCTGGAGTTCACCCCAGCTAATACTAACTGTTCTAGTCCTTCTAATACTATCGTCTCTAATCTACATCAAATACAAAAAACTAGAGGGCCCGCGGTTCGAAGGTAAGCCTATCCCTAACCCTCTCCTCGGTCTCGATTCTACGCGTACCGGTCATCATCACCATCACCATTGAgagctcgcatgcactagt

>3300014208Sub.Fusexin1-V5-6His (pBPT07)EDITED

From 1 to 1827.

ggtaccATGAACCCAATAAAACCCATATTTCTATCAATACTCATAATCGCTATCTTAATTCCCTCCTCTGTCGCCTCCACTACAGCAGGA 90

M N P I K P I F L S I L I I A I L I P S S V A S T T A G 30

TCAGGATTTACTGTAATGTCAATATCCCCATCTAAAATAATTTCCAACGATGAAGACTTAAAAACTGCCAATTTCATCGTAACAGTCGTA 180

S G F T V M S I S P S K I I S N D E D L K T A N F I V T V V 60

GCAAACGGAAATTCTCAATCTATCATCGGAGAACTCCTCACCGCCAATGACATCAATGCTAAACTCTCCGAATCAGGCTCCTCAGTTCGA 270

A N G N S Q S I I G E L L T A N D I N A K L S E S G S S V R 90

GTTTCCTCCCCCTTTAAAATTGAAATGGGTTCAGTCAAAGAAACCCTAACTTACCCCATCCGTAACACAGGCGCAATTCGTAAATACGAA 360

V S S P F K I E M G S V K E T L T Y P I R N T G A I R K Y E 120

TATGCATATTCATCTGAAGTTAATTTTGTTTCGGAAGCAACATGTCTATCAGGCTATTCATATTGTTATACAGTCGAGGCAGGTTCCACA 450

Y A Y S S E V N F V S E A T C L S G Y S Y C Y T V E A G S T 150

TGGTGGGGAAAAACATACAGAAGTCTCAATATTCGCCAAGCAAACAGCGCCGTCTCCTCTTCCCTCGGAGAACTCGATAATCCAAACATT 540

W W G K T Y R S L N I R Q A N S A V S S S L G E L D N P N I 180

AATTGGCAGGGCGATATAGTTCTAACAGCAGGACTAACTCGTGTATCAAAAACAATAGGTTCAATCGAATCCCTTGGAGCAGCAGATTTC 630

N W Q G D I V L T A G L T R V S K T I G S I E S L G A A D F 210

TATAACAGTAACGAGTGGATAGCTCAAGCTACATGGACAGGTTCTTTACTCACAGGGCAAGCAACCCCCAATCCTTCTCTCTATAAAGCA 720

Y N S N E W I A Q A T W T G S L L T G Q A T P N P S L Y K A 240

ACCTTTACTCCCGCCTTTAATCAATGGAGAATCTCCTCAAATTCCTACTATAATGACTATGCATCCTCCCTATCTGCAAAACAAGCATAC 810

T F T P A F N Q W R I S S N S Y Y N D Y A S S L S A K Q A Y 270

TTCCCCTCAATGTTAAGCCAATATACTAAACAAGTGTGTCTTGATTCATACGGTTGTCCATCAGAAACTCCCCTAACAAACGCAATCAGC 900

F P S M L S Q Y T K Q V C L D S Y G C P S E T P L T N A I S 300

AATCATAATAGCATTGTAGACATCCTCTTATCTCAAAACACTAAAGTAACATACACCTCCTCTACAGGAGAAACTCTAGCCCAATCAACA 990

N H N S I V D I L L S Q N T K V T Y T S S T G E T L A Q S T 330

TCAGGAACTTCCTACTCAGGTAACGTAATCGAGACAACCAATCGTCGTATCTCCAATCCTGTAATCGTGTTCAAAGTCAAAGCATTATCC 1080

S G T S Y S G N V I E T T N R R I S N P V I V F K V K A L S 360

TTATCTCCCTACATTCCCGTCGGAAAACCACGCATCGAATCAATAACAACACCCGAATTCTCTAGCGGTGACAACAATGGAGTAGCAACA 1170

L S P Y I P V G K P R I E S I T T P E F S S G D N N G V A T 390

GTTCAATTCAAAAATATCGGTACTGAATCAGCAACATTCTCAGCAACATTCTTTGATCCCTCAGGAACTTTCCACCCCTCCTCTAATCAA 1260

V Q F K N I G T E S A T F S A T F F D P S G T F H P S S N Q 420

GAATCCTCAAAAACTACATGCCCTGCCCTTTCCCAATGCATCATAAACGTCTTTATCTCCAGTGACTCCCCTACCAATGATCGTATCAGT 1350

E S S K T T C P A L S Q C I I N V F I S S D S P T N D R I S 450

AAAACTGCTACAATGACAGTCTATGACATAAACAAACCTTCCAATTCTGATACAAAAACCTTCGACATAATAATGTCACCTCCTAAAACC 1440

K T A T M T V Y D I N K P S N S D T K T F D I I M S P P K T 480

TGTATCCCTAGTTCCACTCGCACCTCAGGAAAACTCATCTATACCTGTAACGCCGACGGTTCAGGGGAAACAGTAATAGAATGCACAGGA 1530

C I P S S T R T S G K L I Y T C N A D G S G E T V I E C T G 510

ACAATCTCCCTTCAAAACAGCAAATACATTTGCATAGGTGGTTCAGTAACTCCCAAACCCGGAACAACACCAGTACCGAAAACAATATTC 1620

T I S L Q N S K Y I C I G G S V T P K P G T T P V P K T I F 540

GCAACCCTATCTGACTTCTGGAGTTCACCCCAGCTAATACTAACTGTTCTAGTCCTTCTAATACTATCGTCTCTAATCTACATCAAATAC 1710

A T L S D F W S S P Q L I L T V L V L L I L S S L I Y I K Y 570

AAAAAACTAGAGGGCCCGCGGTTCGAAGGTAAGCCTATCCCTAACCCTCTCCTCGGTCTCGATTCTACGCGTACCGGTCATCATCACCAT 1800

K K L E G P R F E G K P I P N P L L G L D S T R T G H H H H 600

CACCATTGAgagctcgcatgcactagt 1827

H H * 609

Synthesized plasmid: pBPT08

3300014208-Ga0172379-10001592-871560-40 (Methanomicrobia)

>3300014208Sub.Fusexin1-V5-6His (pBPT08)EDITED

**Addition of restriction Ez. Sites**

**5’ KpnI, 3’ SacI, SphI, SpeI**

ggtaccATGATGAACAAAATGAGAAGCACGATAAAAATATCAATATTTTTTTTATTGATATTCACACTAACAATACCAACTGTTAGTGCAGAAACAGACGGATTTACATTAAAAGCTGCAACAATATCACATCCAGTATCAAACAATAAAGATTTAGAAGATGCAAACATGACACTTCTTGTTGGATCTGGCGGAGGACAATCTTTATATGGAAAATTTGGAAATGAACAATTTACACAATGGATTCCAAATCTATTGTTTAAATATCCACTTGTGTTTGAACTTGATTCGATAAAAGAAACTGCAGATTATCAAATCAGAAATCAAGGAGACTTAAAGAAACTTGAATATACTATAAGAGGAAACTGGTTCTATTCTGCTAAATGCAATCCAGATATACCATATTGTTTTGATGTCGGTTCTGGAAAAATAATTGAAGTAAGATACGTTGACGTTGGAAGATATGGTTCACTCGACACGCCAAATTTAAATTTTAAAGGAAAAGCAACAGTAACAATAAATGGTGTTCCTTATACAAAAGAAATTTCAAATATAATAAGTAGCATTCAGTTCAATTCAATAACAGGAGAATTTGTTGCTTTAATAAAATGGGATGGTTCTTCTGTAACAGGGCAAGGATTACCAGATCAACATAATTACGTTCCAGTGAATAAAAAAGATAGCGGAGAATGGACTCTGTCAACATTATCTTTATATAAAGAATATGATGATTCACTTCTTAAAACAGAAATAGAATTAAACAACAAAAAAGAACAAAAGAGAAAGTTTGATAATAAATTCTATGAATATGCTGCAGAAAAATATGTAGATAAATACTCTATATGTAAAGATAACGAATGTTCACCAATTGTAAACATTGTAAATGAACATAATGCAAAATTAACAAAATTGTTAGATTCAAGAGAAAAAATTGGTTATGAATCATCTATATCATCCGTACAAAATTCATATAAAGATGGAAATGTTTTAGTTGTCTTAAACAGAAGAATAAACATTCCTGAATTTACAATAATAACAACTGCTAAAAGTGTAGGAGCATACATCCCTGTCGGTGAATTTCAAATAATCGATATAACATCACCAACATTCAGTTCAGGAGATAATAATGGAAAGGTTGATGTAAAAGTAAGAAACATTGGAAACTATAGAGGAACATTCTCATTAACTTTAATAGATGAGACTAACACATTTAAACAAATATCGAATTCAAAATCGCTCGAAACATCACTCGAACCTGGAAAAGAAACAATAATCACAATGTATATATCAAGTGGTATAATATCAAGCGATATTACAAAAACTGCAAAGATAGAAGTTTATGACATAAATAATCCGAATAATTTAACATCGAAAGACTTTACTGTATCAGTAACAACACCAAAAATATGTCAACCTGATTCACAAAGATTAGATAACAAAAAGGTGTATACATGTTCTAATGATGGTATGAGAGAATATCTAACATTAGATTGTTCAAATGGAATTATAGACTATAATAACAATACTTATGATTGTACACAAATAGAATACAATAAAGATTTGGTTATAACAAATGCTGAAACACCAATCAAAATAGTCGAAGTACAAAACAAAACAAAACCAGAAGAACCTAAAGAAGAAACACCGAGTTTAATATACTGGCTAGTTGGATTTGTTATACTTAATATATATATAGCATATATAAGAAAATACAAAAATAAAGATGAAACGATAACTTCTGAAAATATACAAAAATCAGTAATTCTATCTGGACTAATAATAGGAACACTCTATTTGTTAATACAATTTACTTATCTGATAGAAACACTAGGTAGTATAATCTGGTTGATATTTATAATGGCTGTAGTAATAATAGGACTTATTAATGTATATTTAAAAGTAATATATCACATGAACATACCAAAAATATATATTATAACAGCAAGTATAATATTGTTTATAATCCTATCAAATATAGCAATTGGAATAAAAGAAACCGCTTGTGATAGTTGGTCAACGAGATGGTTAGCAAGTAAACTATTTGATACTTGTAAAGAATGGACTATAACAGATTACCTACCAGAATGGTTGAAACTAGAGGGCCCGCGGTTCGAAGGTAAGCCTATCCCTAACCCTCTCCTCGGTCTCGATTCTACGCGTACCGGTCATCATCACCATCACCATTGAgagctcgcatgcactagt

>3300014208Sub.Fusexin1-V5-6His (pBPT08)EDITED

From 1 to 2205.

ggtaccATGATGAACAAAATGAGAAGCACGATAAAAATATCAATATTTTTTTTATTGATATTCACACTAACAATACCAACTGTTAGTGCA 90

M M N K M R S T I K I S I F F L L I F T L T I P T V S A 30

GAAACAGACGGATTTACATTAAAAGCTGCAACAATATCACATCCAGTATCAAACAATAAAGATTTAGAAGATGCAAACATGACACTTCTT 180

E T D G F T L K A A T I S H P V S N N K D L E D A N M T L L 60

GTTGGATCTGGCGGAGGACAATCTTTATATGGAAAATTTGGAAATGAACAATTTACACAATGGATTCCAAATCTATTGTTTAAATATCCA 270

V G S G G G Q S L Y G K F G N E Q F T Q W I P N L L F K Y P 90

CTTGTGTTTGAACTTGATTCGATAAAAGAAACTGCAGATTATCAAATCAGAAATCAAGGAGACTTAAAGAAACTTGAATATACTATAAGA 360

L V F E L D S I K E T A D Y Q I R N Q G D L K K L E Y T I R 120

GGAAACTGGTTCTATTCTGCTAAATGCAATCCAGATATACCATATTGTTTTGATGTCGGTTCTGGAAAAATAATTGAAGTAAGATACGTT 450

G N W F Y S A K C N P D I P Y C F D V G S G K I I E V R Y V 150

GACGTTGGAAGATATGGTTCACTCGACACGCCAAATTTAAATTTTAAAGGAAAAGCAACAGTAACAATAAATGGTGTTCCTTATACAAAA 540

D V G R Y G S L D T P N L N F K G K A T V T I N G V P Y T K 180

GAAATTTCAAATATAATAAGTAGCATTCAGTTCAATTCAATAACAGGAGAATTTGTTGCTTTAATAAAATGGGATGGTTCTTCTGTAACA 630

E I S N I I S S I Q F N S I T G E F V A L I K W D G S S V T 210

GGGCAAGGATTACCAGATCAACATAATTACGTTCCAGTGAATAAAAAAGATAGCGGAGAATGGACTCTGTCAACATTATCTTTATATAAA 720

G Q G L P D Q H N Y V P V N K K D S G E W T L S T L S L Y K 240

GAATATGATGATTCACTTCTTAAAACAGAAATAGAATTAAACAACAAAAAAGAACAAAAGAGAAAGTTTGATAATAAATTCTATGAATAT 810

E Y D D S L L K T E I E L N N K K E Q K R K F D N K F Y E Y 270

GCTGCAGAAAAATATGTAGATAAATACTCTATATGTAAAGATAACGAATGTTCACCAATTGTAAACATTGTAAATGAACATAATGCAAAA 900

A A E K Y V D K Y S I C K D N E C S P I V N I V N E H N A K 300

TTAACAAAATTGTTAGATTCAAGAGAAAAAATTGGTTATGAATCATCTATATCATCCGTACAAAATTCATATAAAGATGGAAATGTTTTA 990

L T K L L D S R E K I G Y E S S I S S V Q N S Y K D G N V L 330

GTTGTCTTAAACAGAAGAATAAACATTCCTGAATTTACAATAATAACAACTGCTAAAAGTGTAGGAGCATACATCCCTGTCGGTGAATTT 1080

V V L N R R I N I P E F T I I T T A K S V G A Y I P V G E F 360

CAAATAATCGATATAACATCACCAACATTCAGTTCAGGAGATAATAATGGAAAGGTTGATGTAAAAGTAAGAAACATTGGAAACTATAGA 1170

Q I I D I T S P T F S S G D N N G K V D V K V R N I G N Y R 390

GGAACATTCTCATTAACTTTAATAGATGAGACTAACACATTTAAACAAATATCGAATTCAAAATCGCTCGAAACATCACTCGAACCTGGA 1260

G T F S L T L I D E T N T F K Q I S N S K S L E T S L E P G 420

AAAGAAACAATAATCACAATGTATATATCAAGTGGTATAATATCAAGCGATATTACAAAAACTGCAAAGATAGAAGTTTATGACATAAAT 1350

K E T I I T M Y I S S G I I S S D I T K T A K I E V Y D I N 450

AATCCGAATAATTTAACATCGAAAGACTTTACTGTATCAGTAACAACACCAAAAATATGTCAACCTGATTCACAAAGATTAGATAACAAA 1440

N P N N L T S K D F T V S V T T P K I C Q P D S Q R L D N K 480

AAGGTGTATACATGTTCTAATGATGGTATGAGAGAATATCTAACATTAGATTGTTCAAATGGAATTATAGACTATAATAACAATACTTAT 1530

K V Y T C S N D G M R E Y L T L D C S N G I I D Y N N N T Y 510

GATTGTACACAAATAGAATACAATAAAGATTTGGTTATAACAAATGCTGAAACACCAATCAAAATAGTCGAAGTACAAAACAAAACAAAA 1620

D C T Q I E Y N K D L V I T N A E T P I K I V E V Q N K T K 540

CCAGAAGAACCTAAAGAAGAAACACCGAGTTTAATATACTGGCTAGTTGGATTTGTTATACTTAATATATATATAGCATATATAAGAAAA 1710

P E E P K E E T P S L I Y W L V G F V I L N I Y I A Y I R K 570

TACAAAAATAAAGATGAAACGATAACTTCTGAAAATATACAAAAATCAGTAATTCTATCTGGACTAATAATAGGAACACTCTATTTGTTA 1800

Y K N K D E T I T S E N I Q K S V I L S G L I I G T L Y L L 600

ATACAATTTACTTATCTGATAGAAACACTAGGTAGTATAATCTGGTTGATATTTATAATGGCTGTAGTAATAATAGGACTTATTAATGTA 1890

I Q F T Y L I E T L G S I I W L I F I M A V V I I G L I N V 630

TATTTAAAAGTAATATATCACATGAACATACCAAAAATATATATTATAACAGCAAGTATAATATTGTTTATAATCCTATCAAATATAGCA 1980

Y L K V I Y H M N I P K I Y I I T A S I I L F I I L S N I A 660

ATTGGAATAAAAGAAACCGCTTGTGATAGTTGGTCAACGAGATGGTTAGCAAGTAAACTATTTGATACTTGTAAAGAATGGACTATAACA 2070

I G I K E T A C D S W S T R W L A S K L F D T C K E W T I T 690

GATTACCTACCAGAATGGTTGAAACTAGAGGGCCCGCGGTTCGAAGGTAAGCCTATCCCTAACCCTCTCCTCGGTCTCGATTCTACGCGT 2160

D Y L P E W L K L E G P R F E G K P I P N P L L G L D S T R 720

ACCGGTCATCATCACCATCACCATTGAgagctcgcatgcactagt 2205

T G H H H H H H * 735

Synthesized plasmid: pBPT10

3300018015-Ga0187866_1000629|915963_9

3300018015Sub.Fusexin1-V5-6His (pBPT10) EDITED

**Addition of restriction Ez. Sites**

**5’ KpnI, 3’ SacI, SphI, SpeI**

SpeI edited ATACTAGT to ATTCTAGT both encoding for Ileu

ggtaccATGAGAAAAAAGAATAGGAACATAATAGCAGGAGTTATtCTAGTTGTTGCTATCTTGATTATTCTTGTTTCCTTCGGAGCATTCAAGCTTCCTTTTTCTGTAATCAATTCTGGATTTTCAACATTATCATTAAGCAGTATAAATGTAAATTCAAATGACCCTGTCCTCGGAGGGCAGACTTGGTTTCTTACAGTTTCTCAAAATGGAGCTTCACAATCAGCGTATGTTGATAATTCTGCTGTCTTAAAAGATGACACTACAGGTGCAACATCAAATCCAATGACAATTTCATTAACCCTTAATAAGAATTATGCTTCTTACAATATATTCAACCAAGGAGTTCCTATAAGGCATCTGGAATACACCACAACATCTTACACTGCAATAAATCTTTTGACTGGAACAAGCGGATGCAACACTGCAGTCTGGAACAATTATCAAATATTTCAGCAAGTATCTTATTGCTTTAATTATGTGACAGATGGATATTATGGAACAATAGGACAAGCAAATCAAGTATTTCAGTCAACAGTAAATGTTCAAGGTTCAGGTGGCTCTGATTCCTGCACAATTTCAAACACAGGAGCAAATACATGCTATTCCCAAGCTGGAAATTTATATGCTTCATGGGTTGGAAGCCTTGTTTCTGGGGCATCTGCTCCTGACCCAAGTTCTCAAGGAATAGTTGCAGTTTATAATACTGCTACAGGAGGATGGAAGACAGCAAATCAAAACTCATATCAGACATATTCACAGCAGAACATAAATTCCTGCATAACTTCTGGAGGAACTAATTGCTTTAATCAGTATAATAATTATGAAAGTGCATTTATGAATGGTTATTCTTTCACATCCACTGGAGGAAATGTTGCATACACGCAAGGAACACAAAATAGTGGGCAGGTTATTTTAAATCTTCCACAGCAATTCAACTTCCCTGTAATTACAATGGGGATAAAGGCAAGTATGGTTGGAATAAATATTCTTTCTGGAAAACCAAAAATATTGTCTGCAACCCCGCAAGTATTCCAGACAGGACAAACAGGAACAATTCAAGCCACTGTTGAAAATGTTGGAACAGCAACAGGAAGCTTTAATGTGGGAGCAACATGCACAAATGGATTTTCTCAGACAGGAAATTCATTGACAATTTCTTCTCTTGCCCCAAATTCCCAGAACACAGTTTATATTCCTATAACCGCAAATGTTGTAAGCGGAACAAATGAAGGAACATGCACAGTAACAGCTACTGACATAAGCAATTCATTGAATTCAGACACTAAGACAGTTTCAGTAAGTGCAAATGCAATAATAATCTGCACAAACGGTCAAATATCAACTAATGGCAATTTGATACAGCAATGCACAAACAATGTTTGGGTAACTATCAAATCATGTAATCCAGCAAATGAAACTGCTCAATTGACAGGCAGTGGAGCAAGTTGTGTTCCTAAAAACTCACCAGGAACTTGCGGATTCTTAGGAGTAGGATGCTTGTGGAATTCAATATTTGGAAGCGGAGGATTCTTTGATAACTTATTTTCAGGAATAGGAAATTTCTTCAGCATAGTAAAATTAATTGCAAGTATTTTGGCAGGTATATTTGCATTCGTCTTTAGCTTAGATTTGTTTAACAAGATAAGTTTTGTAAAAAATCATAAATGGTTGCTTTGGATTATATCATTGATAATTGGATTGCTTTTCTTCTGGCTTGTAACAACAGTATTTTGGATAGGAGTAATTTTATTTGTAGTATATCTTATATTTAAATCAATTATTGGAGCTACTCCAATTGGACAAGCATTTAAATTATTAAAAAAACTAGAGGGCCCGCGGTTCGAAGGTAAGCCTATCCCTAACCCTCTCCTCGGTCTCGATTCTACGCGTACCGGTCATCATCACCATCACCATTGAgagctcgcatgcactagt

3300018015Sub.Fusexin1-V5-6His (pBPT10) EDITED

From 1 to 1938.

ggtaccATGAGAAAAAAGAATAGGAACATAATAGCAGGAGTTATtCTAGTTGTTGCTATCTTGATTATTCTTGTTTCCTTCGGAGCATTC 90

M R K K N R N I I A G V I L V V A I L I I L V S F G A F 30

AAGCTTCCTTTTTCTGTAATCAATTCTGGATTTTCAACATTATCATTAAGCAGTATAAATGTAAATTCAAATGACCCTGTCCTCGGAGGG 180

K L P F S V I N S G F S T L S L S S I N V N S N D P V L G G 60

CAGACTTGGTTTCTTACAGTTTCTCAAAATGGAGCTTCACAATCAGCGTATGTTGATAATTCTGCTGTCTTAAAAGATGACACTACAGGT 270

Q T W F L T V S Q N G A S Q S A Y V D N S A V L K D D T T G 90

GCAACATCAAATCCAATGACAATTTCATTAACCCTTAATAAGAATTATGCTTCTTACAATATATTCAACCAAGGAGTTCCTATAAGGCAT 360

A T S N P M T I S L T L N K N Y A S Y N I F N Q G V P I R H 120

CTGGAATACACCACAACATCTTACACTGCAATAAATCTTTTGACTGGAACAAGCGGATGCAACACTGCAGTCTGGAACAATTATCAAATA 450

L E Y T T T S Y T A I N L L T G T S G C N T A V W N N Y Q I 150

TTTCAGCAAGTATCTTATTGCTTTAATTATGTGACAGATGGATATTATGGAACAATAGGACAAGCAAATCAAGTATTTCAGTCAACAGTA 540

F Q Q V S Y C F N Y V T D G Y Y G T I G Q A N Q V F Q S T V 180

AATGTTCAAGGTTCAGGTGGCTCTGATTCCTGCACAATTTCAAACACAGGAGCAAATACATGCTATTCCCAAGCTGGAAATTTATATGCT 630

N V Q G S G G S D S C T I S N T G A N T C Y S Q A G N L Y A 210

TCATGGGTTGGAAGCCTTGTTTCTGGGGCATCTGCTCCTGACCCAAGTTCTCAAGGAATAGTTGCAGTTTATAATACTGCTACAGGAGGA 720

S W V G S L V S G A S A P D P S S Q G I V A V Y N T A T G G 240

TGGAAGACAGCAAATCAAAACTCATATCAGACATATTCACAGCAGAACATAAATTCCTGCATAACTTCTGGAGGAACTAATTGCTTTAAT 810

W K T A N Q N S Y Q T Y S Q Q N I N S C I T S G G T N C F N 270

CAGTATAATAATTATGAAAGTGCATTTATGAATGGTTATTCTTTCACATCCACTGGAGGAAATGTTGCATACACGCAAGGAACACAAAAT 900

Q Y N N Y E S A F M N G Y S F T S T G G N V A Y T Q G T Q N 300

AGTGGGCAGGTTATTTTAAATCTTCCACAGCAATTCAACTTCCCTGTAATTACAATGGGGATAAAGGCAAGTATGGTTGGAATAAATATT 990

S G Q V I L N L P Q Q F N F P V I T M G I K A S M V G I N I 330

CTTTCTGGAAAACCAAAAATATTGTCTGCAACCCCGCAAGTATTCCAGACAGGACAAACAGGAACAATTCAAGCCACTGTTGAAAATGTT 1080

L S G K P K I L S A T P Q V F Q T G Q T G T I Q A T V E N V 360

GGAACAGCAACAGGAAGCTTTAATGTGGGAGCAACATGCACAAATGGATTTTCTCAGACAGGAAATTCATTGACAATTTCTTCTCTTGCC 1170

G T A T G S F N V G A T C T N G F S Q T G N S L T I S S L A 390

CCAAATTCCCAGAACACAGTTTATATTCCTATAACCGCAAATGTTGTAAGCGGAACAAATGAAGGAACATGCACAGTAACAGCTACTGAC 1260

P N S Q N T V Y I P I T A N V V S G T N E G T C T V T A T D 420

ATAAGCAATTCATTGAATTCAGACACTAAGACAGTTTCAGTAAGTGCAAATGCAATAATAATCTGCACAAACGGTCAAATATCAACTAAT 1350

I S N S L N S D T K T V S V S A N A I I I C T N G Q I S T N 450

GGCAATTTGATACAGCAATGCACAAACAATGTTTGGGTAACTATCAAATCATGTAATCCAGCAAATGAAACTGCTCAATTGACAGGCAGT 1440

G N L I Q Q C T N N V W V T I K S C N P A N E T A Q L T G S 480

GGAGCAAGTTGTGTTCCTAAAAACTCACCAGGAACTTGCGGATTCTTAGGAGTAGGATGCTTGTGGAATTCAATATTTGGAAGCGGAGGA 1530

G A S C V P K N S P G T C G F L G V G C L W N S I F G S G G 510

TTCTTTGATAACTTATTTTCAGGAATAGGAAATTTCTTCAGCATAGTAAAATTAATTGCAAGTATTTTGGCAGGTATATTTGCATTCGTC 1620

F F D N L F S G I G N F F S I V K L I A S I L A G I F A F V 540

TTTAGCTTAGATTTGTTTAACAAGATAAGTTTTGTAAAAAATCATAAATGGTTGCTTTGGATTATATCATTGATAATTGGATTGCTTTTC 1710

F S L D L F N K I S F V K N H K W L L W I I S L I I G L L F 570

TTCTGGCTTGTAACAACAGTATTTTGGATAGGAGTAATTTTATTTGTAGTATATCTTATATTTAAATCAATTATTGGAGCTACTCCAATT 1800

F W L V T T V F W I G V I L F V V Y L I F K S I I G A T P I 600

GGACAAGCATTTAAATTATTAAAAAAACTAGAGGGCCCGCGGTTCGAAGGTAAGCCTATCCCTAACCCTCTCCTCGGTCTCGATTCTACG 1890

G Q A F K L L K K L E G P R F E G K P I P N P L L G L D S T 630

CGTACCGGTCATCATCACCATCACCATTGAgagctcgcatgcactagt 1938

R T G H H H H H H * 646

Synthesized plasmid: pBPT11

3300000868-JGI12330J12834-1000008-299010-8 (Halobacteria), **(Fsx1 characterized)**

3300000868- Sub.Fusexin1-V5-6His (pBPT11)EDITED

**Addition of restriction Ez. Sites**

**5’ KpnI, 3’ SacI, SphI, SpeI**

SacI GAGCTC to GAGCCC both encoding for Ala

Start codon GTG to ATG

ggtaccATGAGACGTGCAGCATTGATTCTGGCTTTCGTACTGTTCATCGGTCTTTCCTCGGCTACTGTTACCTCGGCTGATTCAATCACGTATAACTCTGGAACCAGCGAGTTCTTCGACGGAGATGTCTTTGCAATAGAGGTAACCGCTGACCAGTCGACTGATGAGATAGATATCTACCTCGGTGCTTCTGAGCTTTCGGAAAAGACGGACGGCGAGGTCAACCAGGACCTCAGTATTGAGTTCACGCACCAGGATTCCAAGCTGAAGTATTCTACTTCGACTTCTGACGAGCTACGGGACATTGTCACTCTTACAACTTACTACGAGGACGGGTTTGACACGGAGCAGGATGCTATAGACGCCATCAAATCAGACTGTTACGATCTGAATCAGAACGGGAATGGAAGCGGAAGGTACAGCAGGTACTACTCAGTTACATCTCCTGTCTATGATTACGAGATCTATTGTTTCCAGAAGAACGAGAAACTGGCTACTCCAGCATACATAGACAACCCGGATGAGATCTTCACTGCAAAGGCTGAGCTACAGGCCGGTGACAAGACGATTCAGTCAGCTACATTGTCTAACGGTGACGCCGGAGACGGAACAGTCACTGACCTCGGTGACTCAAAGATTAGCTGGAACGGGAACCTGGATTTAGGAGCTTCTGAACCGGAGAATAGCCGAGTCATTGCTTTGTACTCGAATGATTTTGAGAACGGGTGGAGGATCGGCAATAAGCAGTCGTACGAGGACTACAAGACGTTCATCGGTGGCGGGGATGCCTACGATCTCCTGATTGACTGGCAGGACGGGACTTACACCGCCTCTGAAGTAGAGGATGAGCTGGTGAATACTGATGCGAACCAGGCTGTTGAGGAGGCCAGTTCTAGCACGACAGACTTGGTGAACGCGAAGGTTAAGGACTCGTCTCTTGACACCGGTTCGTTCGTGTATGATACCCCTGAGCTGCTTTCCTACCCGTCCTTCACAGTCTACGTCGATGCCGGAGAAAACGGATACATCGAGGTAACGAAGCCGACTGGTGACCCCGATATCATCTCCACTTCATCTACAGAAATTAAGGAGGGAGATGAGGGAACTGTAACCGCCACCGTTGAGAATGTCGGAGATGGTGAGGGCGAGTTCTCTGGAAGGCTTTCCAGCTGCGGTGAAGGCTTCAGTATTGTCGACGACCAGAATACGAAGAATGTGGGTGCCGGAGAATCGGTGACATACAGTTTTGATGTCGCTTTCAGTTCTGTCAGCAGTGAGTCGAAGGAGATCTCGGGCAGCTGCACGTTCGAGGTGAACGGCGTCGAGTCCAGTGATTCCACCAGTGTTTCTGTGACCGGTATCCAGCAGAGTGAGTGTAATCCAGGCGATCAGAGACGGGAAAAGAATGAGAATGACCGCTGGGAGATCTACACCTGCCAGGACAACGGCCTGACCTACGAATATGATGTAACTTGTGCAGAGGACGAGAAGGCTGTAGCTCAGGGAGATAACCAGTTTTCCTGTGAGAAACAGGACGATGATTCAGGTGGCGGAGACAACACTGGATCTGACTCCGGACTGTTCTCGAATCTGTTCGGAGGTTCTGGTTCCGGAGATCTGCTTACACAGGTTCACACAGCTCTGTCTATCCTCGCGGGGCTTGTTGCAGGCTTCTTCGGTTATAGAGGAGC**c**CGATGGATTCACGGCGAGACTGATATAAAAGGAGGCTTCAAGCTGGAGTCCCGGAACGTAAGCCGTGTTAAGAGAGGTAGTCCTGTAGCCGGTATTGTCGGAGCTGTACTCGGGTTTGTCGTAGGATACGGTGTTGCCTCAGTCTTCCATCCAGTAGTTCAGATTATAGTGGTTCTGGGAATTGCTGTTGGCTTGTACTATTTCAGGCTAGAGGGCCCGCGGTTCGAAGGTAAGCCTATCCCTAACCCTCTCCTCGGTCTCGATTCTACGCGTACCGGTCATCATCACCATCACCATTGAgagctcgcatgcactagt

3300000868- Sub.Fusexin1-V5-6His (pBPT11)EDITED

From 1 to 2010.

ggtaccaTGAGACGTGCAGCATTGATTCTGGCTTTCGTACTGTTCATCGGTCTTTCCTCGGCTACTGTTACCTCGGCTGATTCAATCACG 90

M R R A A L I L A F V L F I G L S S A T V T S A D S I T 30

TATAACTCTGGAACCAGCGAGTTCTTCGACGGAGATGTCTTTGCAATAGAGGTAACCGCTGACCAGTCGACTGATGAGATAGATATCTAC 180

Y N S G T S E F F D G D V F A I E V T A D Q S T D E I D I Y 60

CTCGGTGCTTCTGAGCTTTCGGAAAAGACGGACGGCGAGGTCAACCAGGACCTCAGTATTGAGTTCACGCACCAGGATTCCAAGCTGAAG 270

L G A S E L S E K T D G E V N Q D L S I E F T H Q D S K L K 90

TATTCTACTTCGACTTCTGACGAGCTACGGGACATTGTCACTCTTACAACTTACTACGAGGACGGGTTTGACACGGAGCAGGATGCTATA 360

Y S T S T S D E L R D I V T L T T Y Y E D G F D T E Q D A I 120

GACGCCATCAAATCAGACTGTTACGATCTGAATCAGAACGGGAATGGAAGCGGAAGGTACAGCAGGTACTACTCAGTTACATCTCCTGTC 450

D A I K S D C Y D L N Q N G N G S G R Y S R Y Y S V T S P V 150

TATGATTACGAGATCTATTGTTTCCAGAAGAACGAGAAACTGGCTACTCCAGCATACATAGACAACCCGGATGAGATCTTCACTGCAAAG 540

Y D Y E I Y C F Q K N E K L A T P A Y I D N P D E I F T A K 180

GCTGAGCTACAGGCCGGTGACAAGACGATTCAGTCAGCTACATTGTCTAACGGTGACGCCGGAGACGGAACAGTCACTGACCTCGGTGAC 630

A E L Q A G D K T I Q S A T L S N G D A G D G T V T D L G D 210

TCAAAGATTAGCTGGAACGGGAACCTGGATTTAGGAGCTTCTGAACCGGAGAATAGCCGAGTCATTGCTTTGTACTCGAATGATTTTGAG 720

S K I S W N G N L D L G A S E P E N S R V I A L Y S N D F E 240

AACGGGTGGAGGATCGGCAATAAGCAGTCGTACGAGGACTACAAGACGTTCATCGGTGGCGGGGATGCCTACGATCTCCTGATTGACTGG 810

N G W R I G N K Q S Y E D Y K T F I G G G D A Y D L L I D W 270

CAGGACGGGACTTACACCGCCTCTGAAGTAGAGGATGAGCTGGTGAATACTGATGCGAACCAGGCTGTTGAGGAGGCCAGTTCTAGCACG 900

Q D G T Y T A S E V E D E L V N T D A N Q A V E E A S S S T 300

ACAGACTTGGTGAACGCGAAGGTTAAGGACTCGTCTCTTGACACCGGTTCGTTCGTGTATGATACCCCTGAGCTGCTTTCCTACCCGTCC 990

T D L V N A K V K D S S L D T G S F V Y D T P E L L S Y P S 330

TTCACAGTCTACGTCGATGCCGGAGAAAACGGATACATCGAGGTAACGAAGCCGACTGGTGACCCCGATATCATCTCCACTTCATCTACA 1080

F T V Y V D A G E N G Y I E V T K P T G D P D I I S T S S T 360

GAAATTAAGGAGGGAGATGAGGGAACTGTAACCGCCACCGTTGAGAATGTCGGAGATGGTGAGGGCGAGTTCTCTGGAAGGCTTTCCAGC 1170

E I K E G D E G T V T A T V E N V G D G E G E F S G R L S S 390

TGCGGTGAAGGCTTCAGTATTGTCGACGACCAGAATACGAAGAATGTGGGTGCCGGAGAATCGGTGACATACAGTTTTGATGTCGCTTTC 1260

C G E G F S I V D D Q N T K N V G A G E S V T Y S F D V A F 420

AGTTCTGTCAGCAGTGAGTCGAAGGAGATCTCGGGCAGCTGCACGTTCGAGGTGAACGGCGTCGAGTCCAGTGATTCCACCAGTGTTTCT 1350

S S V S S E S K E I S G S C T F E V N G V E S S D S T S V S 450

GTGACCGGTATCCAGCAGAGTGAGTGTAATCCAGGCGATCAGAGACGGGAAAAGAATGAGAATGACCGCTGGGAGATCTACACCTGCCAG 1440

V T G I Q Q S E C N P G D Q R R E K N E N D R W E I Y T C Q 480

GACAACGGCCTGACCTACGAATATGATGTAACTTGTGCAGAGGACGAGAAGGCTGTAGCTCAGGGAGATAACCAGTTTTCCTGTGAGAAA 1530

D N G L T Y E Y D V T C A E D E K A V A Q G D N Q F S C E K 510

CAGGACGATGATTCAGGTGGCGGAGACAACACTGGATCTGACTCCGGACTGTTCTCGAATCTGTTCGGAGGTTCTGGTTCCGGAGATCTG 1620

Q D D D S G G G D N T G S D S G L F S N L F G G S G S G D L 540

CTTACACAGGTTCACACAGCTCTGTCTATCCTCGCGGGGCTTGTTGCAGGCTTCTTCGGTTATAGAGGAGCcCGATGGATTCACGGCGAG 1710

L T Q V H T A L S I L A G L V A G F F G Y R G A R W I H G E 570

ACTGATATAAAAGGAGGCTTCAAGCTGGAGTCCCGGAACGTAAGCCGTGTTAAGAGAGGTAGTCCTGTAGCCGGTATTGTCGGAGCTGTA 1800

T D I K G G F K L E S R N V S R V K R G S P V A G I V G A V 600

CTCGGGTTTGTCGTAGGATACGGTGTTGCCTCAGTCTTCCATCCAGTAGTTCAGATTATAGTGGTTCTGGGAATTGCTGTTGGCTTGTAC 1890

L G F V V G Y G V A S V F H P V V Q I I V V L G I A V G L Y 630

TATTTCAGGCTAGAGGGCCCGCGGTTCGAAGGTAAGCCTATCCCTAACCCTCTCCTCGGTCTCGATTCTACGCGTACCGGTCATCATCAC 1980

Y F R L E G P R F E G K P I P N P L L G L D S T R T G H H H 660

CATCACCATTGAgagctcgcatgcactagt 2010

H H H * 670
